# When ferns go BAHD: characterization of acyltransferases responsible for shaping the phenolic landscape of the fern *Neoblechnum brasiliense*

**DOI:** 10.1007/s00425-026-05206-5

**Published:** 2026-10-27

**Authors:** Maximilian Ufland, Francy J. Pérez-Llanos, Sebastian Triesch, Jochen Hecht, Maike Petersen

**Affiliations:** 1 Institut für Pharmazeutische Biologie und Biotechnologie, Philipps-Universität Marburg, Robert-Koch-Str. 4, 35037 Marburg, Germany; 2 West German Genome Center, Biological and Medical Research Center (BMFZ), Heinrich-Heine-University, Medical Faculty and University Hospital Düsseldorf, Universitätsstr. 1, 40225 Düsseldorf, Germany

**Keywords:** BAHD hydroxycinnamoyltransferase, ω-Hydroxypalmitate, (Hydroxy)phenyllactate, Quinate, Shikimate, Tryptophan

## Abstract

**Main conclusion:**

47 BAHD acyltransferase sequences were retrieved in a transcriptome of the rosmarinic acid-accumulating fern *Neoblechnum brasiliense*. One sequence encoded a hydroxycinnamoyltransferase accepting (hydroxy)phenyllactates leading to the formation of rosmarinic acid.

**Abstract:**

The fern *Neoblechnum brasiliense* (Blechnaceae) is known for its accumulation of rosmarinic acid and related phenolic compounds. Previous studies failed to identify the enzyme catalyzing the transfer of a hydroxycinnamoyl moiety onto a phenyllactic acid—the key step in rosmarinic acid biosynthesis. A long-read transcriptome of young and mature fronds of *Neoblechnum brasiliense* was established, yielding 47 transcribed BAHD acyltransferase sequences. The encoded enzymes were tested for catalytic activity using *p*-coumaroyl-CoA and 70 putative acceptor substrates. NbHCT10/11/12/13 primarily accepted shikimic and quinic acids and grouped into BAHD clade V, alongside other enzymes utilizing the same acceptors. NbHCT19/20 acted as ω-hydroxypalmitic acid hydroxycinnamoyltransferases, clustering in clade VI alongside similar enzymes. NbHCT21/22 were also members of clade VI but displayed different substrate acceptance profiles. NbHCT22 acylates tryptophan, while NbHCT21 utilizes phenyllactic, 4-hydroxyphenyllactic and 3,4-dihydroxyphenyllactic acid as acyl acceptors, qualifying it as an enzyme able to form rosmarinic acid. The biochemical characterization of NbHCT21 showed a high affinity for *p*-coumaroyl- and caffeoyl-CoA (K_m_ 9.2 and 4.4 µM, respectively), but rather low affinity for 4-hydroxyphenyllactic acid (K_m_ 3.2 mM). The phylogenetic placement of NbHCT21 indicates a convergent evolution of enzymes capable of synthesizing rosmarinic acid. Experiments on NbCYP98 as the *meta*-hydroxylating enzyme revealed that *p*-coumaroyltryptamine was preferentially hydroxylated along three other *p*-coumaroyl amides. However, not the *p*-coumaroyl but the tryptamine moiety was hydroxylated, presumably forming *p*-coumaroyl-*N*-serotonin. Our transcriptome analysis provides novel results regarding the phenolic metabolism in the fern *Neoblechnum brasiliense*, which has an elaborated spectrum of phenolic compounds, and a basis for future research.

**Supplementary Information:**

The online version contains supplementary material available at https://doi.org/10.1007/s00425-026-05206-5.

## Introduction

The fern *Neoblechnum brasiliense* (Desv.) Gasper and V.A.O. Dittrich, the only species in the genus *Neoblechnum*, belongs to the family Blechnaceae, the suborder Aspleniinae and the order Polypodiales. It is a leptosporangiate fern, that is only found in the Neotropis (Gasper et al. 2016). Ferns in this family are used in traditional medicine to treat various skin ailments and pulmonary afflictions. This is due to their wealth of specialized metabolites, including steroids, terpenoids, flavonoids, lignans, and other phenolic compounds (Waswa et al. 2022). *N. brasiliense* (formerly *Blechnum brasiliense*) accumulates derivatives of caffeic acid such as rosmarinic acid (RA, caffeoyl-(*R*)-3’,4’-dihydroxyphenyllactic acid), caffeoyl-(*R*)-4’-hydroxyphenyllactic acid, caffeoyl-5-*O*-quinic acid (chlorogenic acid) or blechnic acid B (Harborne 1966; Bohm 1968; Andrade et al. 2016; Ufland and Petersen 2026b). While chlorogenic acid is widely present in the plant kingdom, including members of the Blechnaceae (Petersen et al. 2009; Ufland and Petersen 2026b), rosmarinic acid is less abundant and is primarily found in the family Boraginaceae and in the subfamily Nepetoideae of the Lamiaceae, but also sporadically throughout the plant kingdom starting from hornworts (for an overview see Petersen et al. 2009). In ferns besides the Blechnaceae, *Adiantum capillus-veneris* (Pteridaceae; Zeb and Ullah 2017), *Azolla filiculoides* (Salvinaceae; Carballo-Sanchez et al. 2022) and *Diplazium esculentum* (Athyriaceae; Kongsung et al. 2024) have been reported to contain RA.

Since several interesting phenolic compounds in *N. brasiliense* are derived from RA, we became interested in its biosynthesis in this species. The biosynthesis of RA has been established for *Coleus scutellarioides* (syn. *Coleus blumei*) by Petersen et al. (1993). The acyl donor *p*-coumaroyl-CoA is formed by the general phenylpropanoid pathway: elimination of ammonia from l-phenylalanine by phenylalanine ammonia-lyase (PAL; EC 4.3.1.24) followed by a *para*-hydroxylation by cinnamate 4-hydroxylase (C4H; EC 1.14.14.91) and activation with coenzyme A by 4-coumarate coenzyme A-ligase (4CL; EC 6.2.1.12). The acyl acceptor (*R*)-4-hydroxyphenyllactic acid is derived from l-tyrosine through transamination catalyzed by tyrosine aminotransferase (TAT; EC 2.6.1.5) to 4-hydroxyphenylpyruvic acid which is then stereoselectively reduced to (*R*)-4-hydroxyphenyllactic acid by hydroxyphenylpyruvate reductase (HPPR; EC 1.1.1.237) (De-Eknamkul and Ellis 1987; Petersen and Alfermann 1988). Rosmarinic acid synthase (RAS; hydroxycinnamoyl-CoA:hydroxyphenyllactic acid hydroxycinnamoyltransferase), a member of the BAHD acyltransferase superfamily, then transfers the acyl moiety from hydroxycinnamoyl-CoA to the aliphatic OH-group of a hydroxyphenyllactic acid derivative (Petersen and Alfermann 1988; Berger et al. 2006). 3- and 3’-Hydroxylation by cytochrome P450(s) finally leads to RA (Petersen 1997; Eberle et al. 2009; Levsh et al. 2019).

Hydroxycinnamoyltransferases of the BAHD superfamily are generally considered responsible for forming hydroxycinnamic acid esters (e.g., RA, caffeoylshikimic acid and chlorogenic acid and their less hydroxylated precursors) (Moghe et al. 2023). However, the enzymes from other classes, such as the SCPL and GDSL families (Bontpart et al. 2015; Petersen 2016), have also been shown to catalyze these reactions. BAHD acyltransferases are present in plants and fungi. Among other enzymes, chloramphenicol acyltransferases are discussed to be a potential origin of BAHDs (Moghe et al. 2023). The number of BAHD copies increased from one to five in algal species to 50–200 in flowering plants during evolution. Recent research mapped the 164 biochemically characterized BAHDs into 44 ortholog groups. A similar approach with putative BAHD sequences from 52 sequenced plant genomes were mapped into 765 ortholog groups, highlighting the need for further research (Kruse et al. 2022). Ferns are of particular interest here, as despite possessing a complex vascular system, they still reproduce by spores. Improving our understanding of the situation in ferns might help us to understand the evolutionary processes that took place between mosses and seed plants and the burst in BAHD copies.

BAHDs catalyze acyltransferase reactions by transferring an acid (the acyl donor), activated as CoA thioester, onto an alcohol or amine (the acyl acceptor) (D'Auria 2006). The conserved HxxxDG motif plays a direct role in this process, in which the catalytic histidine is responsible for the transacylation, while the second conserved motif, DFGWG, plays a role in substrate binding and positioning (St. Pierre and De Luca 2000). To date, BAHDs are classified into eight clades (clade 0 to VII) that differ in their preferred substrates and their occurrence throughout the plant kingdom (Moghe et al. 2023). Clade 0 is supposed to be only present in algae. Clades IV, V, and VI are found in species ranging from liverworts to angiosperms. Clades I and VII are present in species ranging from mosses to angiosperms, while clades II and III are exclusive to angiosperms. The predominant acyl acceptors range from flavonoids/anthocyanins/phenolic glucosides (clade I), sugar derivatives (clade III), alkaloids (clade III, IV and VI), terpenoids (clade VI), aliphatic amines (clade III, IV, V and VI) to aliphatic alcohols (III, V and VI) and aromatic alcohols (mostly V and VI) (Kruse et al. 2022) showing the broad substrate diversity of BAHD acyltransferases. RASs that have already been characterized are sorted into clade Va [e.g., from *Coleus scutellarioides*, Lamiaceae (Berger et al. 2006) or *Lavandula angustifolia*, Lamiaceae (Landmann et al. 2011)] and Vc [*Phacelia campanularia*, Boraginaceae (Levsh et al. 2019) or *Sarcandra glabra*, Chloranthaceae (Bömeke and Petersen 2025)] indicating a convergent evolution of these enzymes in angiosperms. Previous attempts to obtain BAHDs from *N. brasiliense* were based on homologous sequence search by 5’/3’-RACE PCRs in the transcriptome of *Struthiopteris spicant* (formerly *Blechnum spicant*) and resulted in nine BAHDs (NbHCT1 to 9) but no RAS could be attained (Ufland and Petersen 2026a). The present study presents a novel approach using transcriptome sequencing to gain insight into the spectrum of BAHD acyltransferases and rosmarinic biosynthesis in *N. brasiliense*.

## Material and methods

### Acquisition of plant material

Plant material was collected from the Botanical Garden, Marburg University from young and adult fern fronds of *Neoblechnum brasiliense* (Desv.) Gasper and V.A.O. Dittrich (accession number: 1977/388). The pinnae were frozen in liquid nitrogen and stored at −80 °C.

### Full length total RNA extraction

Total RNA was extracted from *N. brasiliense* samples: B.bra_A_young (1140–3), B.bra_B_young2 (1140–7), B.bra_A_adult (1140–9), and B.bra_B_adult (1140–14) using the Quick-RNA™ Plant Miniprep Kit (Zymo Research; www.zymoresearch.de) with ~15 mg frozen material per 900 µL lysis buffer and intermediate cooling steps on ice every minute in step 2 of the manufacturer’s protocol. RNA quality and quantity were assessed using a NanoDrop spectrophotometer (Thermo Fisher Scientific), the Qubit RNA High Sensitivity (HS) Assay Kit (Thermo Fisher Scientific), and the DNF-471-3-SS Total RNA (15 nt) HS RNA kit (Agilent Technologies) on the Fragment Analyzer system. Fragment profiles, 25S/18S rRNA ratios, and RNA Integrity Numbers (RINs) were analyzed using the PROSize Data Analysis Software version 3.0.1.6 (Agilent Technologies).

### Kinnex full-length RNA library preparation

Kinnex full-length total RNA libraries were prepared according to the PacBio protocol “*Preparing Kinnex™ Libraries Using the Kinnex Full-Length RNA Kit*” (Version 04, March 2024; www.pacb.com/wp-content/uploads/Procedure-checklist-Preparing-Kinnex-libraries-using-the-Kinnex-full-length-RNA-kit.pdf). Four samples were included in the library preparation. For each sample, ~200–300 ng total RNA with an RNA integrity number between 7.0 and 9.8 was used for cDNA synthesis. Barcoding was performed using the Iso-Seq Express 2.0 kit (PacBio), assigning a unique primer barcode to each sample.

The barcoded cDNA samples were amplified and pooled in equal amounts, yielding a total pooled mass of 56 ng. Then, Kinnex PCR and pooling steps were carried out, followed by array formation, nuclease treatment, and clean up using the Kinnex™ Full-Length RNA Kit Bundle (PacBio; www.pacb.com). All steps were performed according to the manufacturer’s instructions.

To evaluate fragment size distribution, a 1:5 dilution of the Kinnex library was analyzed using the Femto Pulse system (Agilent Technologies) with the FP-1002 protocol (165 kb). Smear analysis was performed using the PROSize Data Analysis Software, yielding an average fragment size of approximately 13 kb. The final library concentration was determined using the Qubit dsDNA HS Assay Kit (Thermo Fisher Scientific) and was found to be 52.5 ng/µL.

### Single molecule real-time sequencing

Sequencing of the Kinnex full-length SMRTbell pool library was conducted following PacBio’s standard sample preparation and sequencing guidelines (SMRT Link software version 25.2.0.266456). Sequencing primers and polymerase were bound to each library pool using the Revio polymerase kit (PacBio). Polymerase-bound SMRTbell complexes were purified, achieving a recovery rate of ≥75%, as confirmed using the Qubit dsDNA HS assay (Thermo Fisher Scientific). The library pool, including the internal control, was sequenced on a SMRT Cell 25 M with an on-plate loading concentration on plate of 150 pM. Run conditions included a 1800-min movie time and base kinetics mode. Sequencing was performed on the Revio system.

### Read segmentation and reference-free iso-seq analysis

Raw subreads were processed directly on-instrument using DeepConsensus-enhanced circular consensus sequencing (CCS), generating high-accuracy HiFi reads in real time without the need for external polishing. On average, each sample yielded approximately 8.4 million HiFi reads, totaling 101.78 Gb per genome. These reads exhibited an average base quality of 94.78%, a read quality of Q35, an N50 length of 12.12 kb, and an average of 12 sequencing passes per molecule.

HiFi reads were then subjected to read segmentation using the PacBio Iso-Seq pipeline with MAS-Seq barcoding strategy. A total of 66,576,229 segmented subreads were generated and subsequently demultiplexed based on sample barcodes. Segmented reads were processed through a combination of tools including *Skera*, *Lima*, and *Iso-Seq Refine*. During this stage, cDNA primers and polyadenylation [poly(A)] tails were trimmed and only reads oriented in the 5′ to 3′ direction that contained both primer sequences and a poly(A) tail were retained. This process yielded an average of 16,340,602 full-length, non-concatemer (FLNC) reads per sample, with a primer detection accuracy of 99.6%.

Next, the FLNC reads were clustered de novo at the isoform level using the *Iso-Seq Cluster2* tool, producing an average of 664,380 high-quality consensus isoforms per sample. The barcoded samples were clustered separately to preserve individual transcript profiles. Throughout the pipeline, strict filtering criteria were applied: a minimum CCS read quality of Q20 was required for downstream processing, and poly(A) tails were mandatory for transcript inclusion.

### Search for BAHD acyltransferases

TransDecoder, Fasta Translate and CD-HIT were used on the Galaxy server (https://usegalaxy.eu) (The Galaxy Community 2024). TransDecoder was used on the total transcriptome (sample 1 (1140–3), 2 (1140–7), 3 (1140–9), 4 (1140–14)) to search for all ORFs longer than 300 amino acids starting with a stop codon and ending with the next stop codon (*LongOrfs*). The resulting CDS file was translated into peptide sequences using Fasta Translate. A HMM profile was created from the BAHD sequences published by Kruse et al. (2022) and additional curated sequences (Table S1). This profile was then used for an HMM search with standard settings (http://www.hmmer.org, version 3.4). A second tool, MMseqs2 (www.mmseqs.com), with the *easy-search* function and standard settings, was used to create a second dataset. This dataset was then merged with the first. CD-HIT with standard settings was used to dereplicate the results. Putative BAHD sequences were manually checked by comparing conserved motifs such as HXXXDG and DFGWG. The start codon was determined by multiple sequence alignments with Clustal Omega (www.theclustal.com) of already known BAHD acyltransferases from *N. brasiliense* (Ufland and Petersen 2026a). If multiple start codons were possible, each candidate was evaluated for the presence of a possible Kozak sequence (Hernández et al. 2019).

### Primers, sequencing and gene synthesis

The primers were synthesized commercially by Eurofins Genomics (www.eurofins.com). Sanger sequencing was carried out by Microsynth Seqlab (www.microsynth.com), and gene synthesis was provided by BioCat (www.biocat.com). All commercial kits were used according to the manufacturers’ instructions.

### Full-length PCR and vector construction

Full-length PCR primers were designed to have an overhang of 19 base pairs with the expression vector pET-15b (Novagen) ending in the vectors corresponding restriction site (NdeI forward primer, XhoI reverse primer) and parts of the coding sequence (Table S2). The melting temperature was calculated for the binding part of the primer and was designed to be appr. 68 °C. cDNA was synthesized with the RevertAid First Strand Kit (Thermo Fisher Scientific). Full-length PCR reactions were conducted with the AccuPrime™ *Taq* DNA-Polymerase Kit, High fidelity (Invitrogen) with standard reaction composition and the following cycling program: 8 cycles: 1 min 98 °C, 1 min 76 °C (− 1 °C/step), 2 min 68 °C; 40 cycles: 1 min 98 °C, 3 min 68 °C; final elongation: + 3 min; hold at 4 °C. For some sequences, the annealing temperature had to be lowered to produce an amplicon. PCR products were separated on agarose gels and amplicons of matching size were cut out and extracted with the NucleoSpin Gel and PCR Clean-up Kit (Macherey–Nagel; www.mn-net.com). pET-15b was digested with NdeI and XhoI (both Thermo Fisher Scientific), dephosphorylated with calf intestine alkaline phosphatase (Fermentas) and separated and extracted as mentioned above. Approximately 200 ng linearized vector and 400 ng PCR amplicon were mixed and incubated with *E. coli* EZ for homologous recombination following standard heat-shock protocols, plated onto LB medium containing 0.1 mg/mL ampicillin and cultivated at 37 °C overnight. Single colonies were picked the next day into 4 mL LB medium with 0.1 mg/mL ampicillin and cultivated at 37 °C overnight. The plasmids were isolated with the QIAprep Spin Miniprep Kit (Qiagen) and sequenced. Plasmids with correct sequence were transferred into *E. coli* SoluBL21 and glycerol stocks were made and stored at − 80 °C.

### Heterologous expression and purification of acyltransferases

Erlenmeyer flasks containing 100 mL TB medium with phosphate buffer (Tartoff and Hobbs 1987) and 0.1 mg/mL ampicillin were inoculated with an overnight culture. The flasks were then incubated at 220 rpm and 37 °C until the OD_600_ reached 0.4 to 0.6. Then, 1 mM isopropyl-β-d-thiogalactopyranoside was added, after which the flasks were incubated for 16 h at 180 rpm and 25 °C. Cell cultures were centrifuged for 10 min at 4000 g and 4 °C and the cell pellet frozen in liquid nitrogen. For every gram of pellet 4 mL 50 mM KP_i_ pH 8.0 was added and the cells were left on ice for 1 h. Next, the cells were resuspended and a spatula tip lysozyme was added, mixed and left on ice for another 30 min. The suspension was ultrasonicated four times for 30 s at 0.5 cycles and 100% amplitude on ice with 30 s cooling steps in between. The suspension was then centrifuged for 10 min at 10,000 g*.* The supernatant was adjusted to 10 mM imidazole, 300 mM NaCl, 50 mM KP_i_ pH 8.0 and filled into columns with 1 mL equilibrated ROTI^®^Garose-His/Ni Beads (Carl Roth; www.carlroth.com). The columns were closed and left bobbing on ice for 1 h. The flow-through was discarded and the beads were washed three times with 2 mL washing buffer (20 mM imidazole, 300 mM NaCl, 50 mM KP_i_ pH 8.0). Then, the protein was eluted with three times 1 mL elution buffer (250 mM imidazole, 300 mM NaCl, 50 mM KP_i_ pH 8.0). The elution fractions were pooled, and 2.5 mL was loaded onto a pre-packed PD10 column (Cytiva) equilibrated with 100 mM KP_i_ pH 7.0. The purified, desalted protein was then eluted with 3.5 mL storage buffer (100 mM KP_i_ pH 7.0). The protein concentration was measured according to Bradford (1976) using bovine serum albumin (1 mg/mL) as reference. The recombinant proteins were stored at −20 °C. An empty vector control was treated the same way.

### SDS-PAGE and Western-blot analysis of recombinant BAHD acyltransferases

SDS-PAGE was performed essentially as described by Laemmli (1970), using 20 µL and/or 20 µL 10× concentrated (trichloroacetic acid precipitation) protein solution per lane. Western-blot analysis followed the method of Mahmood and Yang (2012) using a discontinuous buffer system and an Immobilon-P membrane, as outlined in the manufacturer’s instructions (https://www.sigmaaldrich.com/deepweb/assets/sigmaaldrich/product/documents/159/578/ipvh85rug-mk). Mouse 6x-His-Tag monoclonal antibody (Invitrogen, Life Technologies, Darmstadt, Germany; MA1-21,315) was used as the primary antibody, followed by goat anti-mouse IgG Fc, alkaline phosphatase conjugate (Life Technologies; A16087) as secondary antibody. Alkaline phosphatase activity was visualized using nitro blue tetrazolium chloride/5-bromo-4-chloro-3-indolyl-phosphate according to standard protocols (https://www.sysy.com/protocols/blot.php). Fig. S1 shows all successful blots, along with the empty vector control.

### Substrate search for BAHD acyltransferases from *Neoblechnum brasiliense*

A total of 70 putative BAHD acyl acceptors and seven BAHD acyl donors were tested (Table S3). First, the acyl acceptors were tested with *p*-coumaroyl-CoA as the acyl donor. After determining the acyl donors for all BAHDs, the best acyl acceptor was tested with each acyl donor. Pre-testing was necessary to optimize the protein concentration. Similar BAHD acyltransferases were pooled and tested with two to seven acyl acceptors simultaneously. If product hits were identified, one-on-one testing was conducted to verify product formation with a commercial reference substance, if applicable. All tests were conducted alongside purified empty vector controls. An assay contains 20 µL acyl acceptor, 5 µL acyl donor, 100 µL purified NbHCT or empty vector control. For concentrations refer to Table S3. The assays were incubated for 3 h at 30 °C and were stopped with 25 µL 6 N HCl. Depending on the acyl acceptor, two sample preparation methods were used: For method A, the assay was extracted with 500 µL ethyl acetate. The organic phase was evaporated and the residue redissolved in 100 µL 10% acetonitrile, 0.1% formic acid. For method B, 150 µL methanol was added and mixed. The samples were centrifuged at 16,000* g* for 20 min and the supernatant (10 µL for negative ionization, 5 µL for positive ionization) was analyzed by LC/ESI–MS/MS (HPLC: Agilent 1260 series, column: Multospher 120 RP 18–5 µm 250 × 2 mm; solvent A: 0.1% formic acid in water, solvent B: 0.1% formic acid in acetonitrile; 0–10 min: 5% B → 100% B, 10–15 min: 100% B, 15–15.1 min: 100% B → 5% B, 15.1–20 min → 5% B; flow rate: 0.5 mL/min; mass-spectrometer: micrOTOF-Q III with ESI-source, Bruker Daltonics, calibration with 5 mM sodium formate).

### Biochemical characterization of NbHCT21

pH and temperature optima were recorded for NbHCT21 with *p*-coumaroyl-CoA and (*R*)-3-phenyllactic acid as substrates. Substrate-saturation curves were recorded for variable concentrations of (*R*)-3-phenyllactic acid or (*R*)-4-hydroxyphenyllactic acid with a constant *p*-coumaroyl-CoA concentration and variable amounts of *p*-coumaroyl-CoA or caffeoyl-CoA with constant (*R*)-3-phenyllactic acid concentration. Before testing, linear enzyme activity over the whole reaction time and a suitable concentration range for the variable substrate were tested. The constant substrate was adjusted, if possible, to a concentration exceeding five times the corresponding K_m_, to prevent the constant substrate having a negative effect on the velocity. Reaction conditions, assay composition and detection parameters are listed in Tables S4 and S5. The volume of the assays was 125 µL. After stopping the assay with 20 µL 6 N HCl, the assays were extracted twice with 500 µL ethyl acetate each. The pooled ethyl acetate fractions were evaporated and redissolved in 100 µL 35% (v/v) methanol, 0.01% (v/v) *o*-phosphoric acid and quantified as described in Tables S4 and S5 against 0.5 nmol of an external reference substance. As no *p*-coumaroyl-(*R*)-3’-phenyllactic acid and caffeoyl-(*R*)-3’-phenyllactic acid were available, the product formation was quantified by using *p*-coumaroyl-(*R*)-4’-hydroxyphenyllactic acid and caffeoyl-(*R*)-4’-hydroxyphenyllactic acid as reference substances. Kinetic parameters were determined with GraphPad Prism 10 for each triplicate. The molecular masses of recombinant proteins were calculated with Expasy ProtParam (https://web.expasy.org/protparam/).

### Identification and testing of NbCYP98

The sequence encoding CYP98 in *Struthiopteris spicant* (taxid: 114,457, sample code: VITX) was obtained by searching the 1kP database with the CYP98A14 sequence from *Coleus blumei* (UniProt Q84VU3; Eberle et al. 2009). Based on this sequence, the primers were designed to amplify the corresponding sequence in *N. brasiliense* essentially as described above. The coding sequence of NbCYP98 was codon-optimized and synthesized commercially (BioCat). NbCYP98 as well as NADPH:cytochrome P450 reductase from *Coleus blumei* (CbCPR; AM980997) were ligated into the multiple cloning sites I and II, respectively, of the yeast expression vector pESC-Ura (Agilent Technologies). Yeast transformation, protein expression, microsome preparation as well as SDS-PAGE and Western blot analysis were essentially performed as described by Ernst et al. (2022).

Substrate search for NbCYP98A268 was performed with the following esters/amides, which were either commercial substances or products synthesized by NbHCTs: *p*-coumaroyl-5-*O*-shikimic acid, *p*-coumaroyl-3-*O*-quinic acid, *p*-coumaroyl-4-*O*-quinic acid, *p*-coumaroyl-5-*O*-quinic acid, *p*-coumaroyl-(*R*)-4’-hydroxyphenyllactic acid, *p*-coumaroyl-(*R*)-3’,4’-dihydroxyphenyllactic acid, caffeoyl-(*R*)-4’-hydroxyphenyllactic acid, *p*-coumaroyl-*N*-tyramine, *p*-coumaroyl-*N*-anthranilic acid, *p*-coumaroyl-*N*-3-hydroxyanthranilic acid, *p*-coumaroyl-*N*-5-hydroxyanthranilic acid, *p*-coumaroyl-2’-l-threonic acid and *p*-coumaric acid. Each assay consisted of 10 µL 5 mM NADPH, 5 µL 500 µM substrate (see above), 10 µL 100 mM Tris/HCl pH 7.5 and 100 µL microsomal preparation of codon-optimized NbCYP98A268. An additional negative control with the empty vector was performed. The reaction was incubated for 3 h for 25 °C with shaking at 1200 rpm to ensure oxygen supply and stopped with 50 µL 6 N HCl.

*p*-Coumaroyl-(*R*)-phenyllactic acid (NbHCT21), *p*-coumaroyltryptamine (NbHCT5; Ufland and Petersen 2026a), *p*-coumaroyl-ω-hydroxypalmitic acid (NbHCT19/NbHCT20), *p*-coumaroyl-l-tryptophan (NbHCT22) and *p*-coumaroyl-d-glucaric acid (NbHCT21) were synthesized by the corresponding *N. brasiliense* HCT named in brackets and tested as described. The pre-assay used to synthesize the corresponding esters consisted of 30 µL acyl acceptor (Table S3), 60 µL 2.5 mM *p*-coumaroyl-CoA and 100 µL purified NbHCT and was left to incubate at 25 °C overnight. The following day 10 µL 5 mM NADPH and 100 µL microsomal preparation of codon-optimized NbCYP98A268 were added and the assay incubated as described above. An additional negative control with the empty vector was run in parallel. Sample preparation and LC/ESI–MS/MS analysis (negative ionization mode) were performed as described above. Sample preparation was performed according to method A except for *p*-coumaroyltryptamine which was prepared according to method B.

### Phylogenetic analysis of BAHD acyltransferases

For phylogenetic analysis sequences from Kruse et al. (2022), Ufland and Petersen (2026a) and some additional sequences (Table S1) were aligned with MAFFT 7.526 (https://mafft.cbrc.jp; parameters: *–maxiterate 1000 –genafpair –thread 70*) and ensured that the HXXXDG and DFGWG motifs were correctly aligned. IQ-Tree v1.6.10 (www.iqtree.org) was then used to generate a phylogenetic tree (parameters: *-st AA -nt AUTO -ntmax 70 -b 1000 -m LG*+*F*+*G4*). The resulting tree was visualized with iTOL version 7.2 tree (https://itol.embl.de).

### Ortholog analysis of BAHD acyltransferases

For ortholog analysis the 1kp database (db.cngb.org) was searched with the blastp algorithm with one or two representative bait sequences (NbHCT3, NbHCT17, NbHCT21, NbHCT5, NbHCT16, NbHCT46 and NbHCT26, NbHCT24, NbHCT4) of orthologous groups. The results were aligned with Clustal Omega (www.theclustal.com) alongside the sequences used for phylogenetic analysis (Table S1) and a neighbor joining tree with 100 bootstrap replicates was generated with Clustal X v2.1 (clustalx.software.informer.com) and visualized with iTOL version 7.2. Plant lineages forming an orthogroup with the sequences from *N. brasiliense* were determined. As negative control, the described process was repeated, excluding plant lineages that were sorted with the investigated orthogroup, until no plant lineages are sorted to the orthogroup. Then, all the results were combined and used to generate a final tree.

### Blastp search for enzymes involved in the rosmarinic acid biosynthetic pathway

TransDecoder, Fasta Translate, CD-HIT and blastp were used on the Galaxy Europe page (https://usegalaxy.eu) (The Galaxy Community 2024). Transdecoder (150 amino acids), Fasta Translate followed by CD-HIT with standard settings was conducted on the total transcriptome of *N. brasiliense* to get all peptide sequences longer than 150 amino acids.

The peptide sequences of *Anthoceros agrestis* hydroxyphenylpyruvate reductase 2 (AaHPPR2, (Busch 2022), *Anthoceros agrestis* tyrosine aminotransferase (AaTAT, MN922307.1), *Marchantia polymorpha* 4-coumarate coenzyme A-ligase 3 (Mp4CL3, A0A2R6W0T8), *Physcomitrium patens* phenylalanine ammonia-lyase (PpPAL1, A0A2K1L8S9), *Selaginella moellendorffii* cinnamate 4-hydroxylase (SmC4H, EFJ22128.1), *Coleus scutellarioides* (syn. *Coleus blumei*) 3-hydroxylase (ScCYP98A14, AJ427452.2) and *Anthoceros agrestis* caffeoylshikimate esterase (AaCSE, PV962689) served as individual bait sequences for blastp searches on the database. The first ten results each were searched for conserved motifs of respective enzyme classes (Eberle et al. 2009; Janiak et al. 2010; Wang et al. 2017, 2018, 2021; Wohl and Petersen 2020a; Busch and Petersen 2021; de Vries et al. 2021; Fernie 2021; Schwarze and Petersen 2024) aligned with known sequences and depicted with CLC sequence viewer 8.0.0.

## Results

### Identification of BAHD acyltransferase sequences in the transcriptome of *Neoblechnum brasiliense*

The biosynthetic pathway for RA is well established in dicotyledonous angiosperms (Petersen et al. 1993, 2009; Petersen 2013; Ma et al. 2015; Levsh et al. 2019), but in no other plant group—despite the occurrence of RA in e.g., hornworts and ferns (Takeda et al. 1990; Vogelsang et al. 2006; Ufland and Petersen 2026b). In a previous study, we could identify nine distinct BAHD acyltransferases in *N. brasiliense* by RACE-PCR but could not identify an enzyme capable of forming RA (Ufland and Petersen 2026a). Therefore, we took a new approach by sequencing the *N. brasiliense* transcriptome using long-read sequencing. The high-quality consensus isoforms from the four RNA samples (prepared from two young and two adult fern frond tips) were merged and searched for BAHD acyltransferases using two different algorithms, HMMer and MMseqs2 with standard settings. As previous studies identified RA in fern fronds (Ufland and Petersen 2026b), it was hypothesized that its biosynthesis also occurs in these organs. The bait sequences consisted of a curated list of characterized BAHD acyltransferases assembled by Moghe et al. (2023) as well as the nine already identified BAHD sequences from *N. brasiliense* (Ufland and Petersen 2026a). Both algorithms produced the same number of BAHD sequences, while MMseqs2 produced slightly more non-BAHD sequences. The manual checking of the sequences for intact motifs and appropriate sequence length then resulted in the identification of 47 distinct BAHD acyltransferases. The previously published NbHCT1 to 9 could be retrieved in the transcriptome. A collection of the physicochemical data for these proteins is shown in Table S6.

Vector constructs for NbHCT10–47 were made by PCR amplification of the coding sequences and homologous recombination into pET-15b and finally transferred into the expression host strain *E. coli* SoluBL21. As NbHCT14 could not be amplified, it was purchased as codon-optimized synthetic sequence in pET-15b. Heterologous expression and 6xHis-tag purification of the recombinant proteins were analyzed by SDS-PAGE and Western blot to confirm the presence of all NbHCTs (Fig. S1). An empty vector control was treated in the same way.

### Biochemical characterization of BAHD acyltransferases in *Neoblechnum brasiliense*

**Fig. 1:**
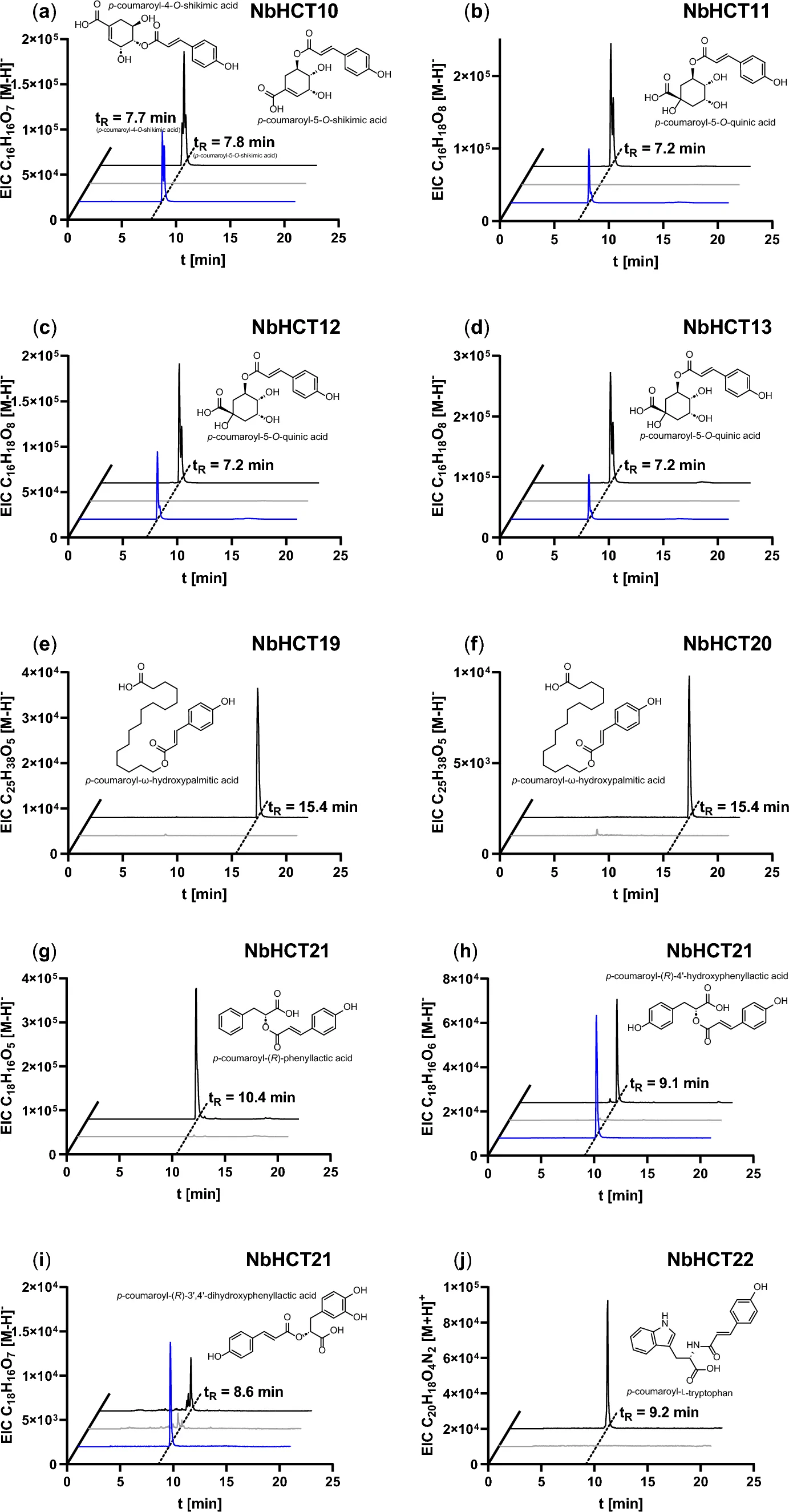
Extracted ion chromatograms (EIC) of selected substrate search assays of BAHD acyltransferases from *N. brasiliense*, t_R_ = retention time. blue line: reference substance, gray line: empty vector control, black line: incubation with NbHCT (shown in top right corner of the graph). Formation of *p*-coumaroyl-5-*O*-shikimic acid (main product) and *p*-coumaroyl-4-*O*-shikimic acid by NbHCT10 (**a**), *p*-coumaroyl-5-*O*-quinic acid by NbHCT11 (**b**), *p*-coumaroyl-5-*O*-quinic acid by NbHCT12 (**c**), *p*-coumaroyl-5-*O*-quinic acid by NbHCT13 (**d**), *p*-coumaroyl-ω-hydroxypalmitic acid by NbHCT19 (**e**), *p*-coumaroyl-ω-hydroxypalmitic acid by NbHCT20 (**f**), *p*-coumaroyl-(*R*)-phenyllactic acid by NbHCT21 (**g**), *p*-coumaroyl-(*R*)-4’-hydroxyphenyllactic acid by NbHCT21 (**h**), *p*-coumaroyl-(*R*)-3’,4’-dihydroxyphenyllactic acid by NbHCT21 (**i**), *p*-coumaroyl-l-tryptophan by NbHCT22 (**j**). For data of all acyl acceptor substrates accepted by their respective enzymes, refer to Tables S7-S14

For the substrate search assays, pre-testing was required to evaluate 38 newly identified BAHD acyltransferases against a panel of 70 acyl acceptors and 7 acyl donors (Table S3). Closely related HCTs were tested with similar acyl acceptors and extended incubation times to enable detection of even low levels of product formation. Whenever potential substrate conversion was observed, individual enzyme assays were performed alongside an empty vector control to confirm product formation. Eight of the 38 investigated BAHD acyltransferases (NbHCT10, 11, 12, 13, 19, 20, 21, 22) exhibited detectable product formation by LC/ESI–MS/MS and will be described in more detail below. Extracted ion chromatograms (EIC) illustrating product formation with the most efficient acyl acceptor for each active NbHCT with *p*-coumaroyl-CoA as donor substrate are shown in Fig. 1.

### Biochemical characterization of NbHCT10

NbHCT10 accepts quinic acid, shikimic acid, and 2,5-dihydroxybenzoic acid as acyl acceptors (Fig. 1a, Table S7). In the presence of *p*-coumaroyl-CoA, it predominantly forms 5-*O*-esters with both quinic acid and shikimic acid, with a clear preference for shikimic acid. Additionally, minor amounts of 4-*O*-esters are produced with shikimic acid. The identities of these esters were confirmed by comparison of retention times and characteristic fragmentation patterns of commercial reference substances of quinic and shikimic acid 3/4/5-*O*-esters, as described by Clifford et al. (2003) (Fig. S2). Cinnamoyl-CoA, *p*-coumaroyl-CoA, caffeoyl-CoA, feruloyl-CoA and sinapoyl-CoA are accepted as acyl donors in combination with shikimic acid as the acyl acceptor. In our previous work, all shikimate HCTs (NbHCT1, PV344582; NbHCT2, PV344583; NbHCT8, PV344589) displayed a less pronounced preference for shikimic acid over quinic acid than the quinate HCT NbHCT3 (PV344584) showed for quinic acid over shikimic acid. The same trend is observed for NbHCT10, which exhibits a product ratio of *p*-coumaroyl-5-*O*-shikimic acid to *p*-coumaroyl-5-*O*-quinic acid of 4:1.

### Biochemical characterization of NbHCT11, 12 and 13

NbHCT11, NbHCT12 and NbHCT13 accept quinic acid and shikimic acid (Fig. 1b, c, d, Tables S8-S10) in combination with *p*-coumaroyl-CoA, with a preference for quinic acid. 2,5-dihydroxybenzoic acid is only accepted by NbHCT12 and 13. When quinic acid is used as the acyl acceptor, only 5-*O*-esters are formed, whereas shikimic acid gives rise to both 4-*O*- and 5-*O*-esters. NbHCT10 (see above) preferentially catalyzes the formation of 5-*O*-esters with shikimic acid, while NbHCT11 and NbHCT13 produce nearly equimolar amounts of the 4-*O*- and 5-*O*-esters (NbHCT10 0.5:1, NbHCT11 1.2:1; NbHCT12 0.4:1, NbHCT13 1.2:1). The product ratio of the *p*-coumaroyl-5-*O*-quinic versus* p*-coumaroyl-5-*O*-shikimic acid esters demonstrates that most quinate transferases cannot transform shikimic acid as good as shikimate transferases quinic acid (NbHCT11 88:1, NbHCT12 23:1, NbHCT13 5:1). NbHCT13 is an exception, as it accepts its secondary substrate with an efficiency comparable to that of NbHCT10. Cinnamoyl-CoA, *p*-coumaroyl-CoA, caffeoyl-CoA, feruloyl-CoA, sinapoyl-CoA, and benzoyl-CoA are utilized by NbHCT11, 12 and 13 with quinic acid as the acyl acceptor. In addition, NbHCT13 accepts acetyl-CoA.

### Biochemical characterization of NbHCT19 and 20

*p*-Coumaroyl transfer by NbHCT19 and NbHCT20 was observed exclusively with ω-hydroxypalmitic acid as the acyl acceptor (Fig. 1e, f, Tables S11, S12). Interestingly, only acyl donors with 4-hydroxylated cinnamic acid derivatives (*p*-coumaroyl-CoA, caffeoyl-CoA, feruloyl-CoA, sinapoyl-CoA) were accepted, suggesting that the 4-hydroxy group is essential for product formation, as cinnamic acid lacking this hydroxyl group is not transferred. The highest peak intensity for NbHCT19 was measured for the formation of feruloyl-ω-hydroxypalmitic acid, whereas NbHCT20 preferentially formed caffeoyl-ω-hydroxypalmitic acid.

### Biochemical characterization of NbHCT21

NbHCT21 accepts phenyllactic acid, 4-hydroxyphenyllactic acid, 3,4-dihydroxyphenyllactic acid and in traces d-glucaric acid and mucic acid with *p*-coumaroyl-CoA as acyl donor (Fig. 1 g, h, i, Table S13). Enzyme assays performed with (*R*)- and (*S*)-phenyllactic acid and *p*-coumaroyl-CoA over 0, 1 and 3 h demonstrated that only (*R*)-phenyllactic acid is accepted (Fig. S3), indicating stereoselective conversion of (*R*)-phenyllactic acid derivatives. As acyl donors cinnamoyl-CoA, *p*-coumaroyl-CoA, caffeoyl-CoA and feruloyl-CoA are utilized in combination with (*R*)-phenyllactic acid, (*R*)-4-hydroxyphenyllactic acid and (*R*)-3,4-dihydroxyphenyllactic acid as acyl acceptors. In addition, the formation of benzoyl-(*R*)-phenyllactic acid was detected. Determination of the pH and temperature optima for the formation of *p*-coumaroyl-(*R*)-phenyllactic acid revealed maximal turnover at pH 7.80 (Fig. S4a) and 38.3 °C (Fig. S4b).

**Fig. 2:**
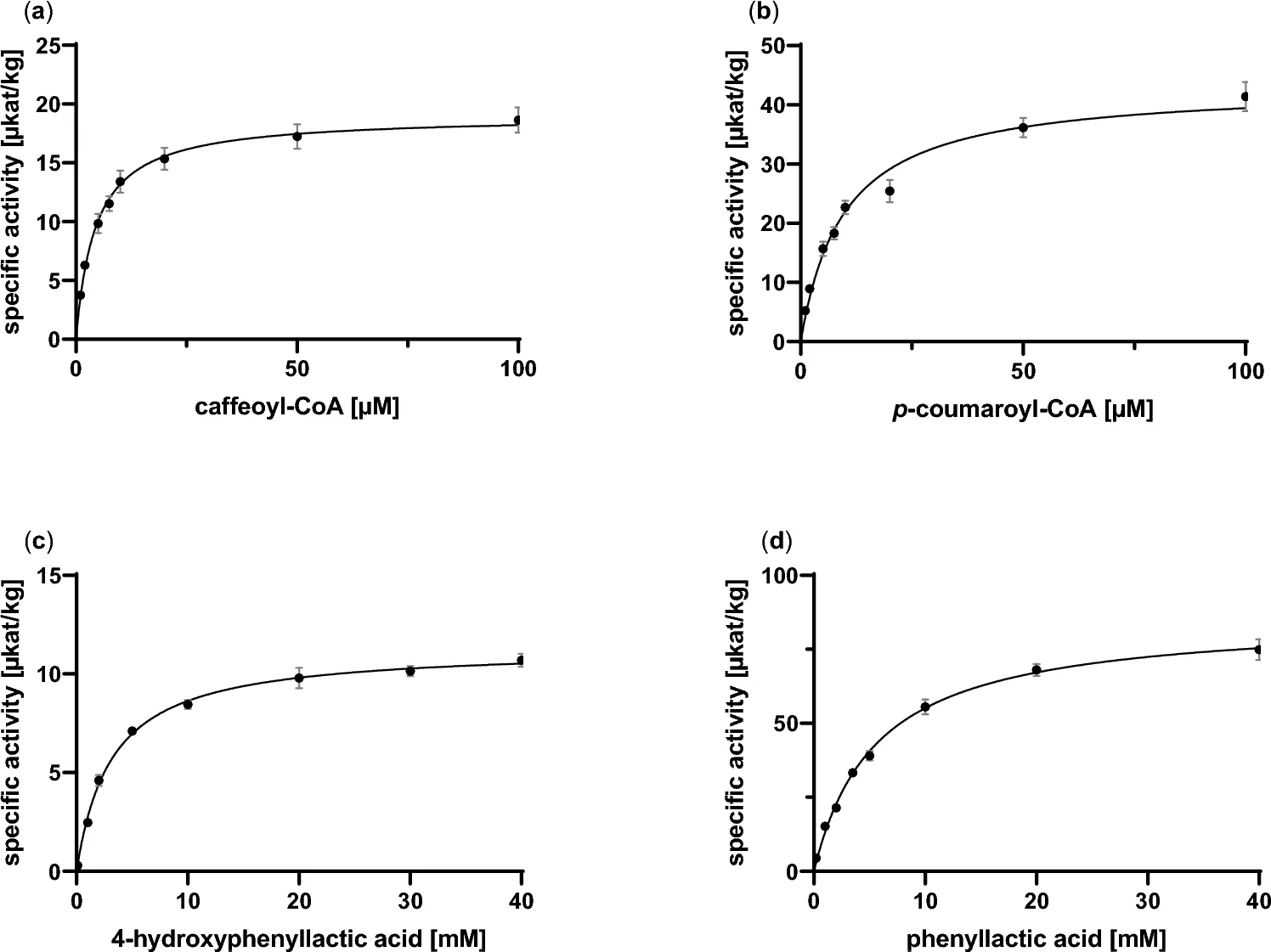
Michaelis–Menten graphs for NbHCT21 with variable concentrations of donor (top two) and acceptor (bottom two) substrates. **a**-**d** Substrate saturation curves for caffeoyl-CoA with (*R*)-phenyllactic acid (**a**), *p*-coumaroyl-CoA with (*R*)-phenyllactic acid (**b**), 4-hydroxyphenyllactic acid with *p*-coumaroyl-CoA (**c**), (*R*)-phenyllactic acid with *p*-coumaroyl-CoA (**d**). *n* = 9 ± SE

Substrate-saturation curves for acyl donors were recorded for variable concentrations of *p*-coumaroyl-CoA and caffeoyl-CoA in the presence of saturating levels of (*R*)-phenyllactic acid (Table 1, Fig. 2a, b). The K_m_ value for caffeoyl-CoA (4.43 µM) is approximately half that of *p*-coumaroyl-CoA (9.17 µM). Likewise, the turnover number for caffeoyl-CoA (0.0010 s^−1^) is about half that observed for *p*-coumaroyl-CoA (0.0023 s^−1^) resulting in nearly identical catalytic efficiencies for caffeoyl-CoA (241.68 l mol^−1^ s^−1^) and *p*-coumaroyl-CoA (267.35 l mol^−1^ s^−1^). At low acyl donor concentrations, caffeoyl-CoA is the preferred substrate, whereas at higher concentrations *p*-coumaroyl-CoA exhibits superior catalytic performance.

**Table 1:** Kinetic data for NbHCT21 (hydroxycinnamoyl-CoA:4-hydroxyphenyllactic acid hydroxycinnamoyltransferase); *n* = 3 × 3 ± SEM. k_cat_ is calculated with the molar mass of the protein (including 6x-His-Tag) of 55.0 kDa

Kinetic data for	With constant substrate	K_m_ [µM]	V_max_ [mkat kg^−1^]	k_cat_ [s^−1^]	k_cat_/K_m_ [l mol^−1^ s^−1^]
caffeoyl-CoA	(*R*)-phenyl-lactic acid	4.43 ± 0.59	0.0189 ± 0.0010	0.0010 ± 0.0001	241.68 ± 25.71
*p*-coumaroyl-CoA	(*R*)-phenyl-lactic acid	9.17 ± 2.20	0.0422 ± 0.0052	0.0023 ± 0.0003	267.35 ± 37.13
(*R*)− 4-hydroxyphenyllactic acid	*p*-coumaroyl-CoA	3150.33 ± 116.14	0.0114 ± 0.0005	0.0006 ± 0.00003	0.20 ± 0.003
(*R*)-phenyllactic acid	*p*-coumaroyl-CoA	5609.00 ± 445.16	0.0861 ± 0.0059	0.0047 ± 0.0003	0.85 ± 0.02
pH optimum	7.80		Temperature optimum	38.3 °C

For acyl acceptors, substrate saturation curves were recorded using varying concentrations of (*R*)-phenyllactic acid and (*R*)-4-hydroxyphenyllactic acid with *p*-coumaroyl-CoA as acyl donor (Table 1, Fig. 2c, d). Assays performed with (*R*)-3,4-dihydroxyphenyllactic acid exhibited very low activities, precluding the determination of substrate-saturation curves with reasonable enzyme concentrations. The K_m_ value for (*R*)-phenyllactic acid (5.61 mM) is about twice the value for (*R*)-4-hydroxyphenyllactic acid (3.15 mM). Notably, compared to other HCTs accepting these substrates the fern enzyme displays relatively low substrate affinity (Sander 2010; Levsh et al. 2019; Bömeke and Petersen 2025). The turnover number for (*R*)-phenyllactic acid (0.0047 s^−1^) is around eight times the value of (*R*)-4-hydroxyphenyllactic acid (0.0006 s^−1^) resulting in an approximately four times better catalytic efficiency for (*R*)-phenyllactic acid (0.85 l mol^−1^ s^−1^) compared to (*R*)-4-hydroxyphenyllactic acid (0.20 l mol^−1^ s^−1^). Still, at low concentrations (*R*)-4-hydroxyphenyllactic acid is preferred over (*R*)-phenyllactic acid. NbHCT21 therefore could be involved in RA biosynthesis in *N. brasiliense*.

Levsh et al. (2019) proposed that a lysine residue in the active site may facilitate complexation of the acceptor’s carboxyl group, as both RAS from *Phacelia campanularia* and *Coleus scutellarioides*, two plant species that also evolved independently, contain this residue (K396 in *C. scutellarioides*, K426 in *P. campanularia*). It is also present in *Sarcandra glabra* RAS (K414). However, this lysine residue is absent in NbHCT21, having been replaced by a more space-consuming arginine residue, which is also basic. This suggests that this fern enzyme may have evolved its RAS activity independently, aided by a different amino acid in the complexation of the acyl acceptor.

### Biochemical characterization of NbHCT22

From the 70 acyl acceptors tested, NbHCT22 converted only tryptophan (Fig. 1j, Table S14). Enzyme assays demonstrated that l-tryptophan is accepted approximately 44-fold more efficiently than d-tryptophan with *p*-coumaroyl-CoA as acyl donor. To our knowledge, this represents the first characterized BAHD acyltransferase reported to accept l-tryptophan as a substrate. Accepted acyl donors include cinnamoyl-CoA, *p*-coumaroyl-CoA, feruloyl-CoA, sinapoyl-CoA and caffeoyl-CoA, with the latter being accepted best. Among the identified BAHD acyltransferases, NbHCT22 exhibits the highest amino acid sequence identity to NbHCT21 (67.0%).

### Phylogenetic and orthogroup analysis of BAHD acyltransferases in *Neoblechnum brasiliense*

**Fig. 3:**
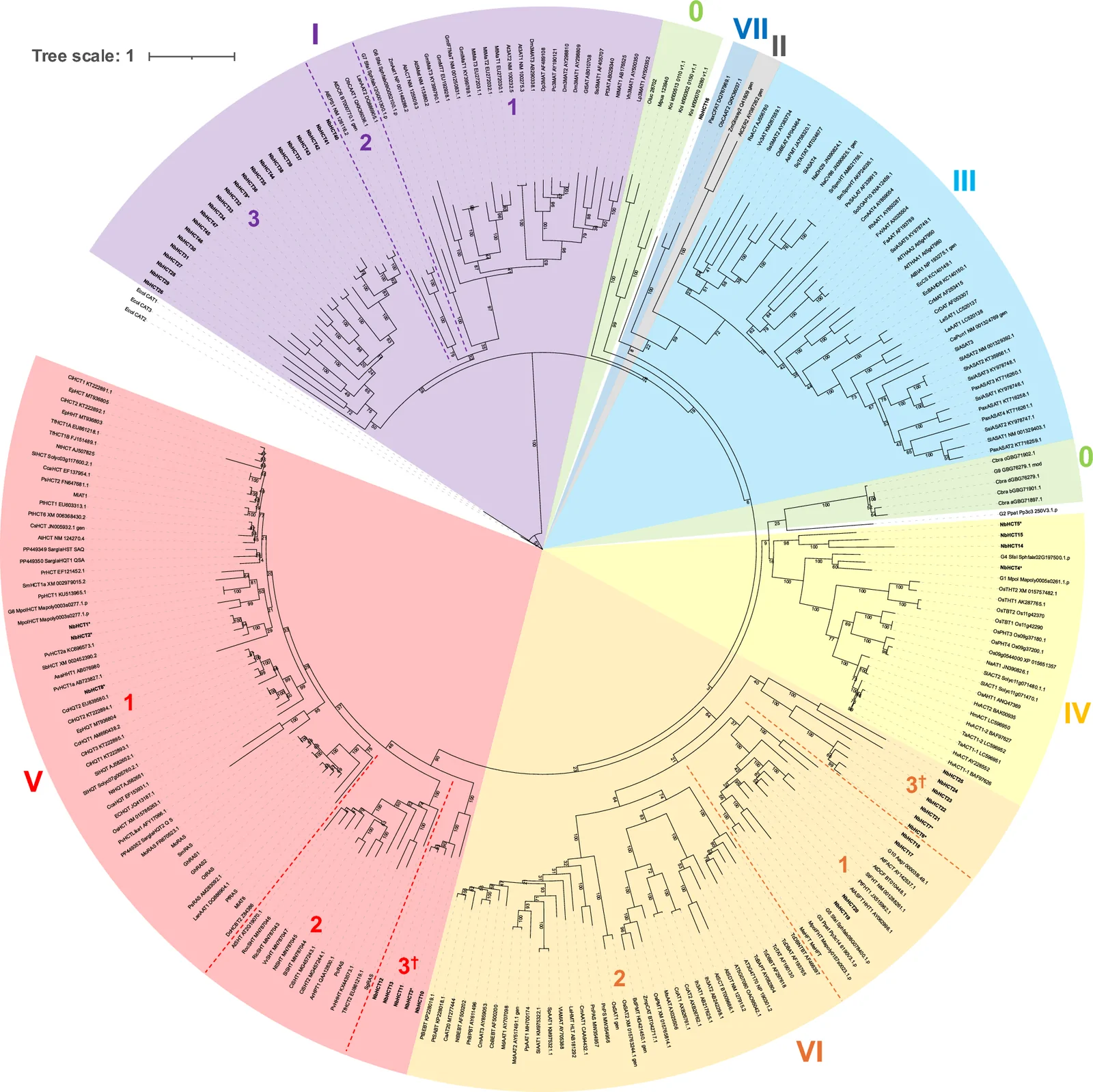
Phylogenetic tree of BAHD acyltransferases using IQ-Tree (LG+F+G4) with 1000 bootstrap replicates. The tree was rooted with three chloramphenicol acyltransferases, a discussed origin of BAHDs. Clades are defined according to D’Auria (2006) and Kruse et al. (2022). Bootstrap values are shown on the branches. BAHDs identified in *Neoblechnum brasiliense* are written in bold letters. BAHDs with an asterisk (NbHCT1 to 9) were already identified in a previous study (Ufland and Petersen 2026a). Depictions of single clades with additional information are shown in Figs S5-S17. ^†)^ New lineage-specific subclades suggested by this work

A phylogenetic tree constructed from 265 verified or putative hydroxycinnamoyltransferase sequences (Table S1) with 1000 bootstrap replicates is depicted in Fig. 3. The clade designations follow the classifications proposed by D’Auria (2006) and Kruse et al. (2022).

Clade VI (Fig. S5) is conserved across all embryophytes and comprises eleven *N. brasiliense* BAHD acyltransferases. Orthogroup analysis revealed that NbHCT17 and 18 have orthologs throughout all embryophyte lineages (Fig. S6). NbHCT21, a putative RAS, which accepts (*R*)-phenyllactic acid derivatives and NbHCT22, which accepts l-tryptophan, cluster within a larger group consisting of NbHCT6, 7, 21–25. Orthogroup analysis showed, that these enzymes are part of a lineage-specific clade restricted to monilophytes (Fig. S7, S8). This group is supported by a bootstrap value of 64 relative to the remaining sequences in clade VI (64). These findings were unexpected, as all previously characterized BAHD acyltransferases involved in rosmarinic acid biosynthesis belong to clades V-1 and V-2. Notably, the acceptance of l-tryptophan represents a novel functional feature. NbHCT19 and NbHCT20, both of which accept ω-hydroxypalmitic acid, are classified within clade VI-1 alongside other ω-hydroxyacid/fatty alcohol hydroxycinnamoyltransferases (FHT).

Clade V (Fig. S9) is conserved across all embryophytes and includes eight *N. brasiliense* BAHD acyltransferases. NbHCT10 to NbHCT13, which accept shikimic and quinic acid to varying extents, cluster together with NbHCT3, a hydroxycinnamoyl-CoA:quinate hydroxycinnamoyltransferase (HQT) described previously (Ufland and Petersen 2026a). Although this group is clearly distinct, it remains strongly linked to the other sequences from clade V (bootstrap value: 90). Orthogroup analysis (Fig. S10) further confirms that these enzymes belong to a lineage-specific clade conserved in members of the order Polypodiales and Cyatheales. Most hydroxycinnamoyl-CoA:shikimate hydroxycinnamoyltransferases (HST) and HQTs cluster within clade V (Moghe et al. 2023). The combination of a high bootstrap support and experimentally confirmed enzymatic activity strongly supports the classification of these *N. brasiliense* BAHDs within clade V. NbHCT1, 2 and 8 (Ufland and Petersen 2026a) have previously been characterized as hydroxycinnamoyl-CoA:shikimate hydroxycinnamoyltransferases (HST) and grouped in clade V-1 along with other HSTs.

Clade IV (Fig. S11) is conserved across all embryophytes and contains four *N. brasiliense* BAHD acyltransferases. NbHCT4, *Sphagnum fallax* Sphfalx02G197500.1.p and *Marchantia polymorpha* Mapoly0005s0261.1.p form an orthogroup that is present in non-seed plants and gymnosperms (Fig. S12), consistent with the findings of Kruse et al. (2022). The remaining three enzymes—NbHCT5, 14 and 15—form an orthogroup found exclusively in non-seed plants (Fig. S13). NbHCT5 was shown to accept tryptamine (Ufland and Petersen 2026a) and is the only Clade IV BAHD from *N. brasiliense* exhibiting high turnover with one of the applied substrates.

Clade I (Fig. S14) is conserved across tracheophytes and bryophytes sensu stricto and 23 *N. brasiliense* BAHDs were assigned to this clade. These sequences (NbHCT9, NbHCT26-47) form a subgroup adjacent to the remaining Clade I BAHDs (bootstrap value: 28). Orthogroup analysis indicates that orthologs of this subgroup are present in monilophytes, lycophytes and gymnosperms (Fig. S15). To date, no enzymatic activity has been reported for Clade I BAHDs in non-seed plants.

Clade VII (Fig. S16) is conserved across tracheophytes and bryophytes sensu stricto and one *N. brasiliense* sequence (NbHCT16) was placed near this clade, although it does not form a monophyletic grouping. One possible explanation is that clade VII is represented by only two angiosperm sequences, highlighting the evolutionary gap between monilophytes and angiosperms. However, orthogroup analysis showed that orthologs of NbHCT16 are present in all tracheophytes (Fig. S17), even though this group does not cluster monophyletically with clade VII BAHDs. The precise classification of NbHCT16 therefore remains unresolved.

### Identification and biochemical characterization of NbCYP98A268

A single copy of the CYP98 family was found in *N. brasiliense* (GenBank PZ166776), which was co-expressed with the *Coleus scutellarioides* CPR (syn. *C. blumei*, NADPH:cytochrome P450 reductase, AM980997; Eberle et al. 2009) in *Saccharomyces cerevisiae* YPH501. Western blotting revealed the successful expression of the codon-optimized NbCYP98A268 (Fig. S18), but no expression of CbCPR. It was assumed that low amounts of CbCPR are still present and the *S. cerevisiae* CPRs are able to reduce NbCYP98A268. A substrate search was conducted with various esters. Four of these substrates were hydroxylated by NbCYP98A268 (Fig. S19, Table S15). The highest peak intensity was observed for *p*-coumaroyltryptamine. Weaker hydroxylated esters were *p*-coumaroyl-*N*-tyramine, *p*-coumaroyl-*N*-3-hydroxyanthranilic acid and *p*-coumaroyl-*N*-anthranilic acid. Analysis of the fragmentation of the hydroxylation product of *p*-coumaroyltryptamine reveals a decarboxylated *p*-coumarate ion (*m/z* 119) and a hydroxylated tryptamine ion (*m/z* 175) indicating that the hydroxylation occurs at the tryptamine moiety of *p*-coumaroyltryptamine. Comparing the hydroxylated *p*-coumaroyl-*N*-3-hydroxyanthranilic and caffeoyl-*N*-3-hydroxyanthranilic acid produced by NbHCT8 (Ufland and Petersen 2026a) showed that they have the same retention time but different fragmentation patterns. This suggests that *p*-coumaroyl-*N*-3-hydroxyanthranilic acid is probably not hydroxylated to caffeoyl-*N*-3-hydoxyanthranilic acid by NbCYP98A268. No MS2 event occurred for *p*-coumaroyl-*N*-anthranilic acid and *p*-coumaroyl-*N*-tyramine due to low product formation, and therefore no statement regarding the hydroxylation position can be made. It can be assumed that the esters are not hydroxylated to the corresponding caffeoyl derivatives either.

### Reconstruction of the phenylpropanoid pathway in *Neoblechnum brasiliense*

**Fig. 4:**
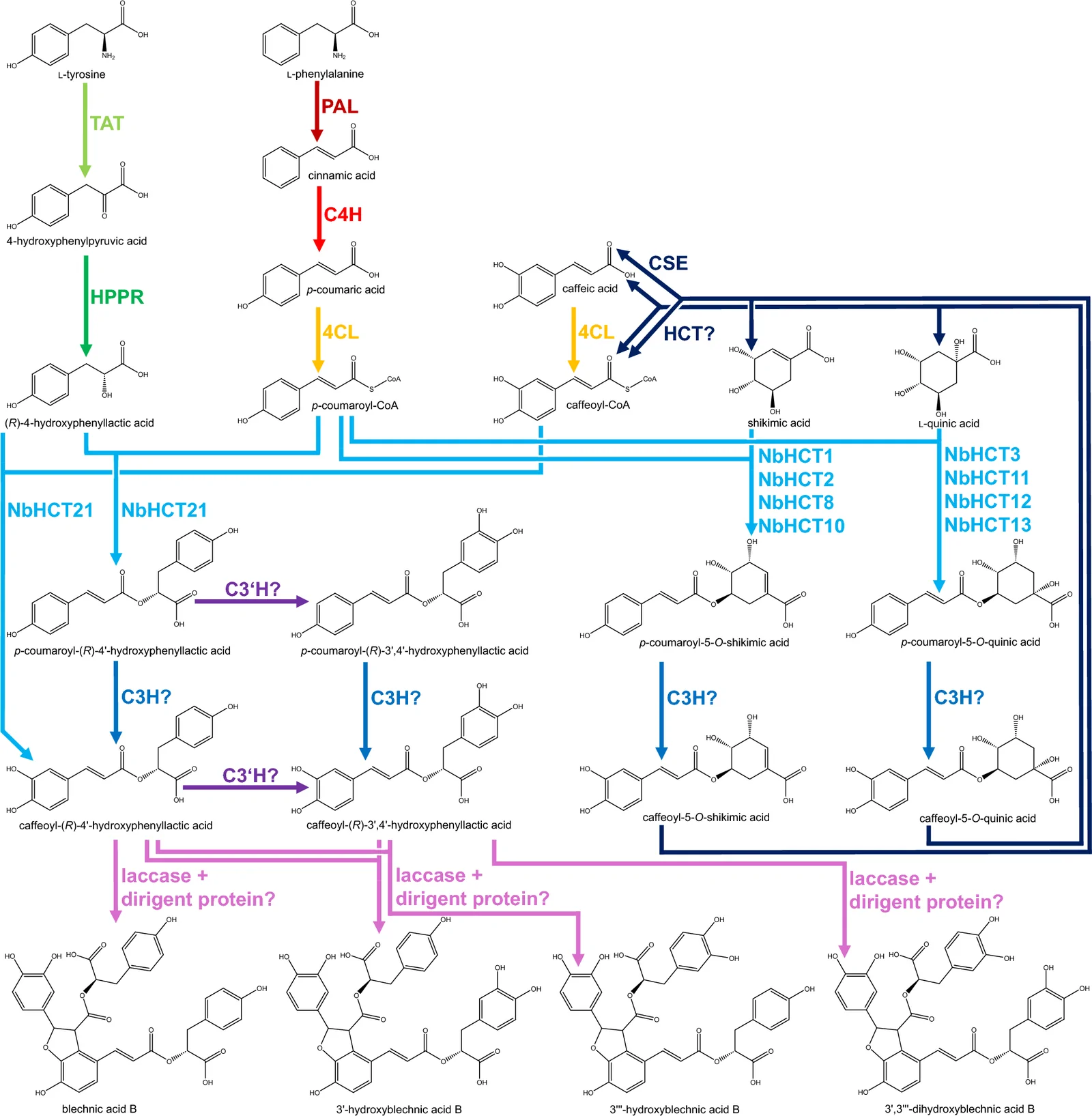
Potential pathway of the biosynthesis of rosmarinic acid and blechnic acids in *Neoblechnum brasiliense*. Enzymes catalyzing the same reaction are shown in the same color. Enzymatic steps that are not yet known or unlikely are marked with a question mark. TAT, tyrosine aminotransferase; HPPR, hydroxyphenylpyruvate reductase; PAL, phenylalanine ammonia-lyase; C4H, cinnamate 4-hydroxylase; 4CL, 4-coumarate coenzyme A-ligase; HCT, hydroxycinnamoyltransferase; C3H, 3-hydroxylase; C3’H, 3’-hydroxylase; CSE, caffeoylshikimate esterase

The sequence of biosynthetic steps for the formation of RA in Lamiaceae was proposed by Petersen et al. (1993). This was used as a starting point for the reconstruction of a hypothetical biosynthetic pathway towards RA in *N. brasiliense* which is summarized in Fig. 4. The biosynthesis of 4-hydroxyphenyllactic acid starts with tyrosine aminotransferase (TAT) which catalyzes the conversion of l-tyrosine to 4-hydroxyphenylpyruvic acid. A putative TAT homolog (GenBank PZ166773) with high similarity to characterized TATs from bryophytes and angiosperms with pyridoxal 5'-phosphate binding sites and important catalytic amino acids was identified (Fig. S20) (Wang et al. 2018; Busch and Petersen 2021).

Next, 4-hydroxyphenylpyruvic acid is stereoselectively reduced to (*R*)-4-hydroxyphenyllactic acid by hydroxyphenylpyruvate reductase (HPPR). Two putative homologs were retrieved (GenBank PZ166774, PZ166775) and aligned with characterized HPPRs and hydroxypyruvate reductase (HPR) from *Anthoceros agrestis* (Fig. S21) (Busch 2022). Conserved motifs such as the NAD(P)H binding site or myristylation sites (Wang et al. 2017) as well as amino acids involved in catalysis and substrate binding (Janiak et al. 2010) were identified in the *N. brasiliense* homologs. These homologs were highly similar to characterized HPPRs from bryophytes and angiosperms and distinctly different from AaHPR, a peroxisomal enzyme involved in photorespiration.

The biosynthesis of the acyl donor starts with l-phenylalanine. Phenylalanine ammonia-lyase (PAL) eliminates ammonia, resulting in the production of *t*-cinnamic acid. Four putative homologs (GenBank PZ166758, PZ166759, PZ166760, PZ166761) could be identified in the *N. brasiliense* transcriptome, all of which having the ASG motif forming the 3,5-dihydro-5-methylidene-4H-imidazol-4-one (MIO) group which is the reactive center of PAL (Fig. S22) (Ritter and Schulz 2004). Cinnamic acid is then hydroxylated by cinnamate 4-hydroxylase (C4H) to produce *p*-coumaric acid. This step is catalyzed by CYP73s in plants. Three putative homologs (GenBank PZ166762, PZ166763, PZ166764) were identified and aligned and found to possess all CYP73 motifs and transmembrane domains (Fig. S23) (Wohl and Petersen 2020a). Next, 4-coumarate coenzyme A-ligase (4CL) activates the acid by forming a thioester with coenzyme A. Caffeic acid is mostly also accepted by 4CLs. Four putative homologs could be retrieved in *N. brasiliense* (GenBank PZ166765, PZ166766, PZ166767, PZ166768). All of these contained the conserved boxes I and II in the alignment (Fig. S24) (Schwarze and Petersen 2024). As PAL, C4H and 4CL make up the general phenylpropanoid pathway that is conserved in all land plants (Fernie 2021; de Vries et al. 2021) and the sequence similarities to the corresponding characterized enzymes from bryophytes and angiosperms are high, we assume that these enzymes in *Neoblechnum brasiliense* may be functional.

Of the hydroxycinnamoyltransferases investigated, NbHCT21 catalyzes the formation of *p*-coumaroyl-(*R*)-4’-hydroxyphenyllactic acid and *p*-coumaroyl-(*R*)-3’,4’-dihydroxyphenyllactic acid and the respective caffeoyl esters. *p*-Coumaroyl-5-*O*-shikimic acid is formed by NbHCT1 (GenBank PV344582) and NbHCT8 (GenBank PV344589) and putatively by NbHCT2 (GenBank PV344583) and NbHCT10, while *p*-coumaroyl-5-*O*-quinic acid is formed by NbHCT3 (GenBank PV344584) and putatively by NbHCT11, 12 and 13. These esters can undergo further hydroxylation in *meta*-position by a 3-hydroxylase, which in land plants usually is a member of the CYP98 family. One CYP98 homolog (GenBank PZ166776) was identified, which is the same as described above. Alignment with known C3H revealed all conserved motifs and transmembrane domains (Fig. S25) (Eberle et al. 2009). Caffeoyl-5-*O*-shikimic acid and caffeoyl-5-*O*-quinic acid can be hydrolyzed by caffeoylshikimate esterases (CSE) releasing caffeic acid (Vanholme et al. 2013). Four homologs (GenBank PZ166769, PZ166770, PZ166771, PZ166772) were identified in the transcriptome of *N. brasiliense*. All of these have two lyase (GxSxG) domains and one acyltransferase (HxxxxD) domain (Wang et al. 2021). While NbCSE2 and NbCSE3 carry a domain specific for monoacylglycerol lipases (MAGL), NbCSE1 and NbCSE4 have a CSE/MAGL3 domain making them putative CSE candidates in *N. brasiliense* (Fig. S26) (de Vries et al. 2021).

## Discussion

*N. brasiliense* is a fern that contains a wide range of phenolic compounds including various hydroxycinnamic acid esters (Harborne 1966; Bohm 1968; Andrade et al. 2016; Ufland and Petersen 2026b). The biosynthesis of these compounds is only partly understood. The reactions leading to *p*-coumaroylshikimic and *p*-coumaroylquinic acid esters are catalyzed by redundant BAHD hydroxycinnamoyltransferases, that have recently been characterized in detail (Ufland and Petersen 2026a). However, the formation of rosmarinic acid remained elusive. Therefore, we established a transcriptome from young and adult fern fronds, which are organs containing RA (0.27% of the dry weight). This allowed us to identify the transcribed members of the BAHD acyltransferase superfamily in fern fronds. 47 BAHD sequences were identified, including the nine sequences already known (Ufland and Petersen 2026a). The number of BAHD sequences in *N. brasiliense* is relatively high compared to other ferns investigated in this respect, such as *Azolla filiculoides* and *Salvinia cucullata*, which have been reported to contain fewer than 20 members of this family (Moghe et al. 2023). Generally, it has been observed that algae contain only a few BAHD genes (1 to 5) and that this number increases significantly in land plants, reaching up to 200 in angiosperms (Moghe et al. 2023).

Following amplification and heterologous expression, the ability of 38 proteins to transfer hydroxycinnamoyl units onto a wide range of substrates for ester and/or amide formation was tested (Table S3). Eight out of the 38 novel encoded enzymes showed catalytic activity in our assays. This is not necessarily due to inactivity of the enzymes but to the range of substrates supplied in the assays. A high number of novel acyltransferases group in clade I where mainly flavonoids are used as substrates. Flavonoid(glycoside)s have, however, not been applied in our assays. The formation of an ester of a hydroxycinnamic acid (*p*-coumaric, caffeic acid) with a phenyllactic acid (phenyllactic, 4-hydroxyphenyllactic and 3,4-dihydroxyphenyllactic acids) was of particular interest, as this reaction leads to the formation of RA. Classifying the newly identified BAHDs from *N. brasiliense* into the established system of eight clades (clade 0 to VII; Kruse et al. 2022; Moghe et al. 2023) revealed the need for reclassification which will be explained below. This reclassification of clades will certainly continue in the future with the addition of further sequences from non-seed plants. In the following, subclades defined by Kruse et al. (2022) are designated by letters, whereas the simplified system proposed in the present study uses Arabic numerals (Fig. S5, S9, S11, S14, S16).

### Clade VI BAHDs

Clade VI BAHDs are primarily involved in the acylation of aromatic and aliphatic alcohols, as well as flavonoid glycosides. Additionally, they play a key role in acylation reactions during paclitaxel biosynthesis (Kruse et al. 2022). Eleven BAHD sequences from *N. brasiliense* (NbHCT 6, 7, 17–25) were found to belong to this clade. NbHCT19 and NbHCT20 were assigned to subclade VI-1 (Fig. 3, S5), which is consistent with their observed enzymatic activities, as all functionally characterized BAHD enzymes within subclade VI-1 accept ω-hydroxypalmitic acid or other aliphatic alcohols as substrates. LC/MS data indicate that NbHCT19 utilizes feruloyl-CoA preferentially, whereas NbHCT20 shows a preference for caffeoyl-CoA. The presence of a *p*-hydroxy group in the acyl donor appears to be essential for catalytic activity, since cinnamoyl-CoA was not accepted as a substrate. The preference for caffeoyl-CoA is uncommon among related enzymes; to date, only *p*-coumaroyl-, feruloyl- and sinapoyl-CoA have been identified as acyl donor substrates (e.g., Lotfy et al. 1994; Gou et al. 2009; Cheng et al. 2013). However, caffeoyl-CoA-specific enzymes have been reported for the formation of root waxes in *Arabidopsis thaliana* tap roots (Kosma et al. 2012).

Similar to subclade VIa, as defined by Kruse et al. (2022), NbHCT17 and NbHCT18 were found to be placed in a group encompassing genes from all embryophytes (Fig. S6). Along with sequences assigned to subclades VIa and VIb—which differ from clade VIa in that they exclude anthocerotophytes and marchantiophytes—these sequences constitute a newly defined monophyletic subclade. This subclade has been designated VI-1 and is present in all embryophytes. However, none of the tested acyl acceptors yielded detectable reaction products for NbHCT17 or NbHCT18.

NbHCT6, 7 and 21 to 25 were assigned to subclade VI-3. Among the enzymes tested, only NbHCT21 and NbHCT22 exhibited enzymatic activity. NbHCT22 accepts l-tryptophan, while NbHCT21 accepts (*R*)-phenyllactic acid, (*R*)-4-hydroxyphenyllactic acid and (*R*)-3,4-dihydroxyphenyllactic acid, which makes it the only candidate as RAS in *N. brasiliense*. These two acyl acceptors share structural similarities in that they both have a carboxyl group and an aromatic system in the α-position relative to the acylated amine or alcohol. They differ in terms of their acylated group, aromatic system and chirality. Therefore, a gene duplication event followed by neofunctionalization appears plausible for these genes. NbHCT21 exhibits a higher turnover number for (*R*)-phenyllactic acid (0.0047 s⁻^1^) than for (*R*)-4-hydroxyphenyllactic acid (0.0006 s⁻^1^) when using *p*-coumaroyl-CoA as the substrate. However, the K_m_ value is lower for (*R*)-4-hydroxyphenyllactic acid (3.2 mM) compared to (*R*)-phenyllactic acid (5.6 mM). This suggests that (*R*)− 4-hydroxyphenyllactic acid may represent the substrate utilized in vivo. In principle, phenylpyruvate aminotransferases could produce phenylpyruvate in plant cells (Yokoyama 2024), which can then be reduced to phenyllactic acid by HPPR as in, e.g., *Coleus scutellarioides* (Kim et al. 2004).

To date, all characterized enzymes that accept (*R*)-4-hydroxyphenyllactic acid belong to subclades V-1 (Va) and V-2 (Vc). RAS from Lamiaceae (Sander and Petersen 2011) are supposed to have diverged from HSTs, whereas RAS from Boraginaceae (Levsh et al. 2019) and Chloranthaceae (Bömeke and Petersen 2025) are thought to have evolved from spermidine hydroxycinnamoyltransferases (SHT). Comparing the kinetic parameters for 4-hydroxyphenyllactic acid together with *p*-coumaroyl CoA as acyl donor reveals that RAS from *N. brasiliense* has high K_m_-values and low reaction velocities (K_m_ 3.15 mM, V_max_ 11.4 µkat kg^−1^) compared to RAS from other species that have been investigated (*C. scutellarioides*: K_m_ 25 µM, V_max_ 20.5 mkat kg^−1^, Sander 2010; *P. campanularia*: K_m_ 875 µM, V_max_ 2.7 mkat kg^−1^, Levsh et al. 2019; *S. glabra*: K_m_ 544 µM, V_max_ 2.6 mkat kg^−1^, Bömeke and Petersen 2025) resulting in a low catalytic efficiency. This might correspond to the observation that RA levels in *N. brasiliense* are on the lower end of the spectrum (0.27% of the DW in pinnae, Ufland and Petersen 2026b; *C. scutellarioides*: 11.1% of the DW in leaves, Tungmunnithum et al. 2019; *P. campanularia*: 1.14% of the fresh weight in leaves, Levsh et al. 2019; *S. glabra*: 3.53% of the DW in young leaves, Bömeke and Petersen 2025). Lignans formed from rosmarinic acid and its precursors were found to be present in *N. brasiliense* (Ufland and Petersen 2026b). The formation of these compounds would lead to a depletion of RA in the plant cells. As the overall activity of NbHCT21 is low relative to other RAS, in vivo validation is required to confirm its role as the enzyme forming RA in *N. brasiliense*.

As already pointed out by Levsh et al. (2019), the enzymes forming RA have evolved from different ancestral enzymes, which can lead to differing catalytic efficiencies. RAS from Boraginaceae and Chloranthaceae have probably evolved from spermidine hydroxycinnamoyltransferases and exhibit considerably lower catalytic efficiencies than RAS from Lamiaceae, which supposedly evolved from HST/HQT. The phylogenetic placement of NbHCT21 in clade VI suggests that its RAS activity evolved independently. This raises the possibility that aliphatic alcohol hydroxycinnamoyltransferases represent a third evolutionary origin of RAS activity, as the only currently characterized non-seed plant BAHDs from clade VI accept aliphatic alcohols. However, further research is required to substantiate this hypothesis.

NbHCT6 and 7 are phylogenetically closely related to NbHCT21 and NbHCT22. Testing substrates with structural similarity to the identified acyl acceptors may help elucidate their function. Orthogroup analysis (Fig. S7) revealed that subclade VI-3 BAHDs are specific to the monilophyte lineage. Therefore, it can be assumed that plants from earlier diverged lineages containing rosmarinic acid, such as *Anthoceros agrestis*, evolved distinct enzymes that are not related to subclade VI-3 BAHDs. The closest homolog of NbHCT21, and currently the only one identified, was found in *Dennstaedtia davallioides* (1kp sample code: MTGC, scaffold 2,044,669), the only fern species from the Dennstaedtiaceae family that has been sequenced to date. The family’s phenolic profile is poorly described (Bohm 1968) highlighting it as a promising starting point for further research on BAHD acyltransferases in monilophytes.

NbHCT24 and 25 were initially included in the orthogroup analysis of NbHCT6, 7, 21–23; however, they were separated during the analysis, so a separate orthogroup analysis was performed (Fig. S8). It was found that orthologs of NbHCT24 and 25 are present in monilophytes. As these sequences share an orthogroup origin with NbHCT6, 7, 21–23 they were placed alongside the mentioned enzymes in clade VI-3.

### Clade V BAHDs

The main substrates acylated by clade V BAHDs are aromatic alcohols, including shikimic, quinic and hydroxyphenyllactic acids, as well as aromatic and aliphatic amines (Kruse et al. 2022). The previously characterized NbHCT1, 2 and 8 preferentially esterify shikimic acid over quinic acid (Ufland and Petersen 2026a) and have been assigned to subclade V-1, which is present in embryophytes (Fig. 3, S9). In contrast, NbHCT3 and NbHCT10 to 13 are sorted into a separate subclade V-3. Orthogroup analysis (Fig. S10) confirmed that subclade V-3 is a lineage-specific clade restricted to the orders Polypodiales and Cyatheales. These two orders share a monophyletic origin, either exclusively (Christenhusz and Chase 2014) or together with the order Salviniales (Nitta et al. 2022). Since most characterized HQT/HST enzymes belong to clade V (Moghe et al. 2023) and there is high bootstrap support for both clade V and the aforementioned subgroup (90), it can be assumed that these enzymes are part of clade V. Of this subgroup, only NbHCT3 has been studied in detail and confirmed to function as an HQT. The LC/MS data presented here suggest that NbHCT11 to 13 prefer quinic acid, whereas NbHCT10 favors shikimic acid. The HQTs cluster together with short branch lengths (Fig. S10) while the putative HST (NbHCT10) diverged from the HQT branch earlier. Taken together, these patterns suggests that quinic acid specificity may have evolved independently several times in the plant kingdom. This hypothesis is further supported by the observation that all previously characterized HQTs cluster in subclade V-1. It remains an open question whether the HQTs in *N. brasiliense* and other Blechnaceae ferns, most of which contain chlorogenic acid (Ufland and Petersen 2026b), originated from a single gene duplication event followed by gain-of-function mutations in an ancestral HST in a last common ancestor of the family, or whether this occurred earlier. Further research is required to resolve this issue.

### Clade IV BAHDs

Clade IV BAHDs primarily use aliphatic amines as acceptor substrates, with some enzymes being involved in alkaloid metabolism (Kruse et al. 2022). Our phylogenetic analysis placed NbHCTs 4, 5, 14 and 15 into clade IV. NbHCT4 clusters with G1 *Marchantia polymorpha* Mapoly0005s0261.1.p and G4 *Sphagnum fallax* Sphfalx02G197500.1.p (Kruse et al. 2022). A second group comprises NbHCT5, 14, 15 (Fig. 3, S11). Orthogroup analysis (Fig. S12, S13) suggests that the first group is present in non-seed plants and gymnosperms, whereas the second group is only found in non-seed plants. All other clade IV BAHDs are present in seed plants and are subdivided into clades IVa and IVb by Kruse et al. (2022), the latter being only present in monocotyledonous plants. In our previous study, an acyl acceptor was only identified for NbHCT5 (Ufland and Petersen 2026a). Tryptamine was acylated with high turnover. This is in accordance with the general assumption that amines are preferred substrates of clade IV BAHDs.

### Clade I BAHDs

The majority of the *N. brasiliense* BAHDs (NbHCT9 and NbHCT26-47) were categorized as belonging to clade I which includes enzymes in flavonoid and anthocyanin transformation (Kruse et al. 2022; Moghe et al. 2023). All NbHCTs in this clade are in a separate subclade I-3 alongside the clade I sequences from other plant species (bootstrap value: 28) (Fig. 3, S14). Orthogroup analysis (Fig. S15) revealed that orthologs of clade I-3 BAHDs are present in monilophytes, lycophytes and gymnosperms. Clade I-1 BAHDs from spermatophytes (Clade Ia, Ib according to Kruse et al. 2022) were shown to accept flavonoid glycosides, anthocyanins and phenolic glucosides (Moghe et al. 2023; Xu et al. 2023). However, because our substrate catalog (Table S3) did not include flavonoids, anthocyanins, or phenolic glycosides as acyl acceptors and due to the limited knowledge about flavonoids in *N. brasiliense* (Waswa et al. 2022), no product formation was observed with the tested substrates. Most active clade I BAHDs have been identified in angiosperms, so further research on non-seed plant clade I BAHDs is necessary.

### NbHCT16 as BAHD without clear association

The position of NbHCT16 is unclear (Fig. 3). It could be placed in clades II, III, or VII; however, BAHDs from clades 0, II, and III have been reported absent in ferns (Moghe et al. 2023). Orthologs of NbHCT16 are present in tracheophytes (Fig. S17).

### Reconstruction of the phenylpropanoid pathway in *Neoblechnum brasiliense*

It is reasonable to assume that the enzymes involved in the early steps of the phenylpropanoid pathway (TAT, HPPR, PAL, C4H, 4CL, CSE; Fig. 4) are functional in *N. brasiliense*. Previous studies have demonstrated that the corresponding gene homologs are established in ferns (de Vries et al. 2021; Fernie 2021). The proteins encoded by homologous genes in extant early-branching lineages have been experimentally confirmed to be functionally active, including those from the hornwort *Anthoceros agrestis* (Wohl and Petersen 2020b, 2020a; Busch and Petersen 2021; Busch 2022; Pezeshki et al. 2022; Marks and Petersen 2026). To learn more about the situation *N. brasiliense*, the transcriptome was searched for homologs of the aforementioned enzymes and their amino acid sequences aligned with characterized sequences to identify conserved motifs. The homologs of all genes involved in RA biosynthesis in seed plants were retrieved. This would make a biosynthesis of RA in a pathway similar to that found in Lamiaceae probable.

At least one TAT and two HPPRs could be active to form 4-hydroxyphenyllactic acid in *N. brasiliense*. The amino acid sequence of NbTAT has a high similarity to the *A. agrestis* TAT, which has been shown to be active (Busch and Petersen 2021). The two NbHPPRs are closely related to other HPPRs, but distinct from plant hydroxypyruvate reductases. *p*-Coumaroyl-CoA could be formed by at least four PALs, three C4Hs and four 4CLs in *N. brasiliense*. All discovered amino acid sequences thus far bear a strong resemblance to previously characterized enzymes and possess the essential motifs.

In a recent study (Ufland and Petersen 2026a) and in this work, four HCTs were found to accept quinic acid, four to accept shikimic acid, and one to accept (*R*)-4-hydroxyphenyllactic acid as an acyl acceptor. It is unclear which of these HCTs contribute to the formation of the respective esters in vivo and to what extent. Therefore, further investigation is required. HCTs can also catalyze the reverse reaction to produce caffeoyl-CoA (Hoffmann et al. 2003; Moghe et al. 2023), however, this reaction is rather ineffective. In contrast, caffeoylshikimate esterase (CSE) effectively releases caffeic acid from caffeic acid esters, suggesting its essential role for the formation of C-, G- and S-monolignols as well as hydroxycinnamic acid derivatives with complex substitution patterns (Vanholme et al. 2013). The hydrolysis of esters by CSEs in *N. brasiliense* is supported by the identification of two CSE candidate genes (*NbCSE2* and *NbCSE3*) in the *N. brasiliense* transcriptome which encode proteins harboring the characteristic CSE motif (Fig. S26). The presence of an active CSE in a non-seed plant has been recently shown for the hornwort *Anthoceros agrestis* (Marks and Petersen 2026).

The CYP98 family encodes aromatic *meta*-hydroxylases, which are found in all land plants. A single copy of this gene exists in all lineages except the angiosperms, in which a dramatic increase in CYP98 copies is observed (Alber et al. 2019). We demonstrated that NbCYP98 accepts *p*-coumaroyl amides with tyramine, anthranilic acid, 3-hydroxyanthranilic acid and tryptamine, but not esters with shikimic, quinic or (*R*)-4-hydroxyphenyllactic acid derivatives. This is in line with the observation in *Anthoceros agrestis*, where the only CYP98 preferred *p*-coumaroyl amides as substrates (Ernst et al. 2022). A preference for *p*-coumaroyl amides was as well described for CYP98 from *Physcomitrium patens*, *Selaginella moellendorffii*, and *Pteris vittata* while gymnosperm and angiosperm CYP98 preferentially accept *p*-coumaroyl esters (Alber et al. 2019). Fragmentation analysis of the reaction products formed from *p*-coumaroyltryptamine indicates hydroxylation at the tryptamine moiety and not at the *p*-coumaroyl moiety as expected. We can assume that hydroxylation is performed at position 5 to *p*-coumaroyl-*N*-serotonin. This is supported by the presence of this ester in bamboo (Tanaka et al. 2003). Studies have shown that *p*-coumaroyl-*N*-serotonin has antifungal activity against *Aciculosporium take* infection in bamboo. This suggests that this compound may be a defense compound that is also found in *N. brasiliense*. Serotonin was tested with all *N. brasiliense* BAHDs, but none accepted it. These results suggest that the biosynthesis of this compound may include NbHCT5 as the initial step in forming *p*-coumaroyltryptamine, which is subsequently hydroxylated by NbCYP98A268 to produce *p*-coumaroyl-*N*-serotonin. Further research is needed to make definitive statements.

There are still some mysteries regarding the biosynthesis of RA in *N. brasiliense*, such as the formation of caffeic acid/caffeoyl-CoA as well as the introduction of the *meta*-hydroxy group in the 4-hydroxyphenyllactate part of the molecule. Several hypotheses could explain the activity of *meta*-hydroxylation in *N. brasiliense*. First, the enzymes could function as part of a metabolon, a multienzyme complex, which facilitates secondary metabolite biosynthesis in plants (Laursen et al. 2016). Second, a different cytochrome P450 family could be involved in *meta*-hydroxylation. Third, an ascorbate peroxidase could catalyze the hydroxylation of *p*-coumaric acid to form caffeic acid, as demonstrated in other plants (Barros et al. 2019).

## Conclusion

In conclusion, a transcriptome of *N. brasiliense* was generated. It comprises 2.6 million high-quality consensus isoforms. Analysis of the transcriptome identified 47 distinct BAHD acyltransferases, of which substrates could be determined for 13 of these. NbHCT21 is of particular interest. It belongs to a monilophyte lineage-specific clade and catalyzes the formation of rosmarinic acid precursors. This makes it the first potential non-seed plant BAHD enzyme with this catalytic activity. However, more research is needed to understand NbHCT21’s role in the in vivo formation of rosmarinic and blechnic acids.

Eight HCTs were shown to utilize shikimic and quinic acids as substrates. The enzyme forming quinic acid esters is associated with a lineage-specific clade with members of the orders Polypodiales and Cyatheales. The other identified putative main acyl acceptors were tryptamine, ω-hydroxypalmitic acid, and l-tryptophan, with the latter representing a novel substrate for BAHD enzymes. For the remaining 34 BAHDs, acyl acceptors have not yet been identified.

Reconstruction of the phenylpropanoid pathway from the *N. brasiliense* transcriptome revealed that early biosynthetic steps are conserved, because functional homologs have been identified in early-diverging embryophytes. In contrast, the hydroxylation steps that lead to rosmarinic acid and chlorogenic acid are not well understood and require further research. It is assumed that NbCYP98A268 is involved in forming *p*-coumaroyl-*N*-serotonin by hydroxylation of the tryptamine derivative.

### Accession numbers

The sequencing data of the transcriptome of *Neoblechnum brasiliense* in this work were deposited in the European Nucleotide Archive under the accession PRJEB111199. The accession numbers of cDNA coding sequences of the BAHD acyltransferases characterized in this work were deposited in Genbank as described in Table S6. The accession numbers of cDNA coding sequences of the enzymes of the rosmarinic acid biosynthesis and the NbCYP98A268 mentioned in this work were deposited in Genbank as described in Fig. S20-S26.

## Supplementary Information

Below is the link to the electronic supplementary material.

Supplementary file1 (PDF 3881 KB)

## Data Availability

The data supporting the findings of this study can be obtained from the supporting information of this article.

## Abbreviations

4CL: 4-Coumarate CoA-ligase
C4H: Cinnamic acid 4-hydroxylase
CSE: Caffeoylshikimate esterase
HCT: Hydroxycinnamoyltransferase
HPPR: Hydroxyphenylpyruvate reductase
PAL: Phenylalanine ammonia-lyase
RA: Rosmarinic acid
RAS: Rosmarinic acid synthase
TAT: Tyrosine aminotransferase
