# Supplementary material for: When ferns go BAHD: characterization of acyltransferases responsible for shaping the phenolic landscape of the fern Neoblechnum brasiliense
Source: Planta. 2026 Oct 27;264(6):177. doi: 10.1007/s00425-026-05206-5 (PMC13710588; doi:10.1007/s00425-026-05206-5)
Supplement: Supplementary file 1 [file 425_2026_5206_MOESM1_ESM.pdf]

Supplementary Material for

When ferns go BAHD: Characterization of acyltransferases responsible for shaping the phenolic landscape of the fern *Neoblechnum brasiliense*

Maximilian Ufland, Francy J. Pérez-Llanos, Sebastian Triesch, Jochen Hecht, Maike Petersen

Supplementary Figures

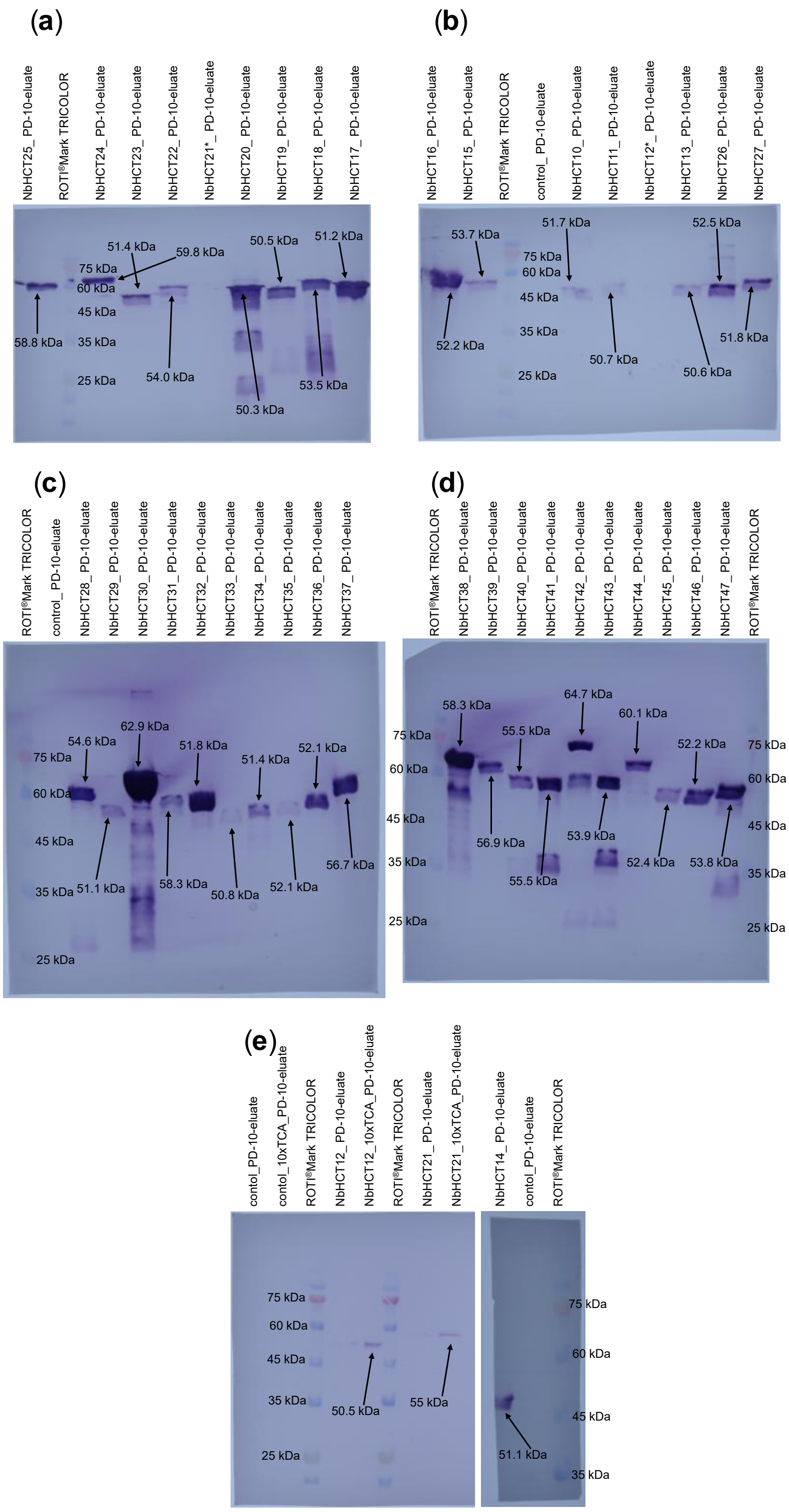

Fig. S1 Western blot analysis of NbHCT10 to NbHCT47. HCTs marked with an asterisk could not be detected in original samples and were 10x concentrated and are shown in (**e**)

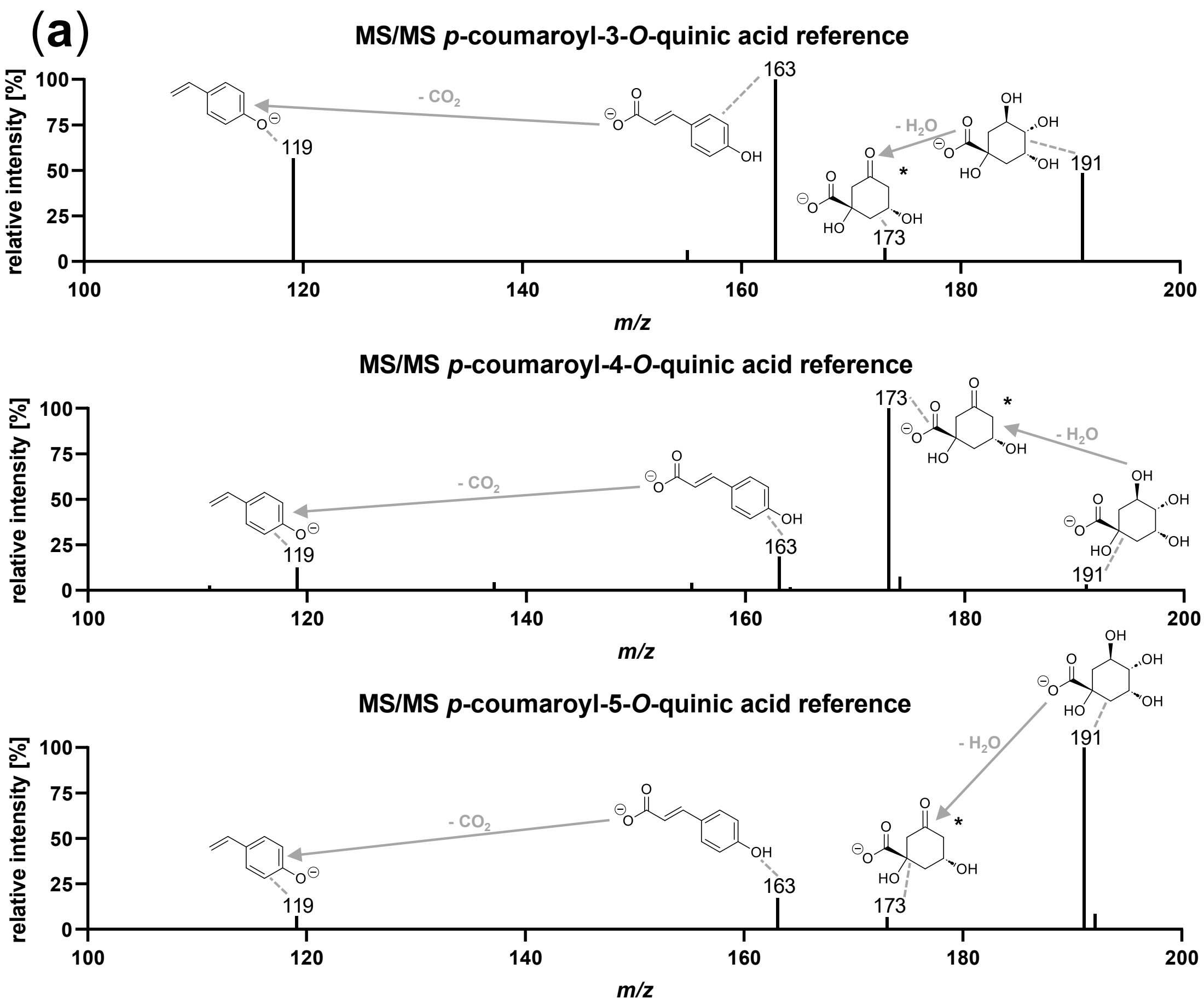
(a)
MS/MS p-coumaroyl-3-O-quinic acid reference
MS/MS p-coumaroyl-4-O-quinic acid reference
MS/MS p-coumaroyl-5-O-quinic acid reference
relative intensity [%]
m/z
- CO2
- H2O
119
163
173
191
OH
HO
O
*
0
25
50
75
100
120
140
160
180
200

MS/MS caffeoyl-3-*O*-quinic acid reference

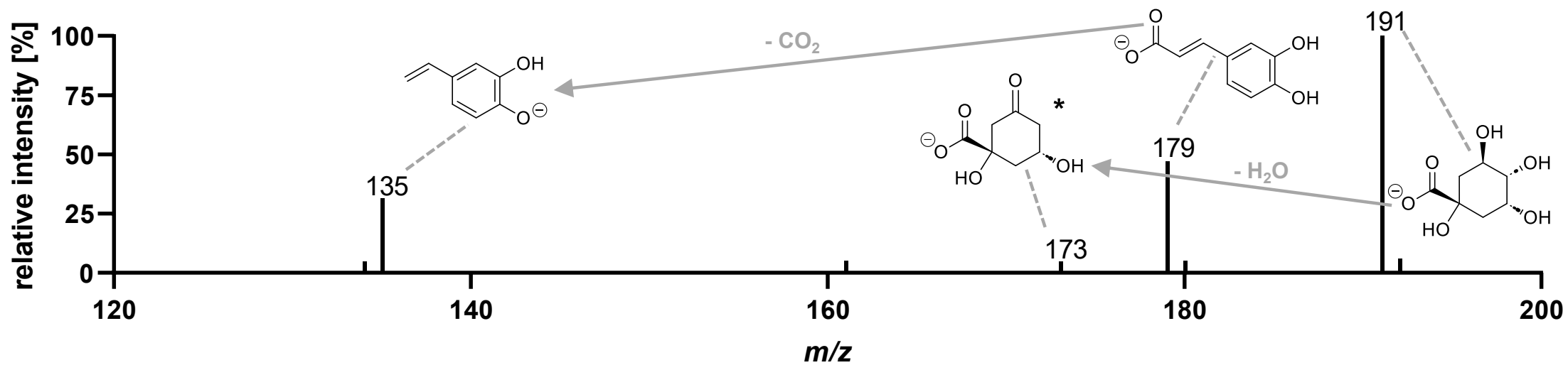

MS/MS caffeoyl-4-*O*-quinic acid reference

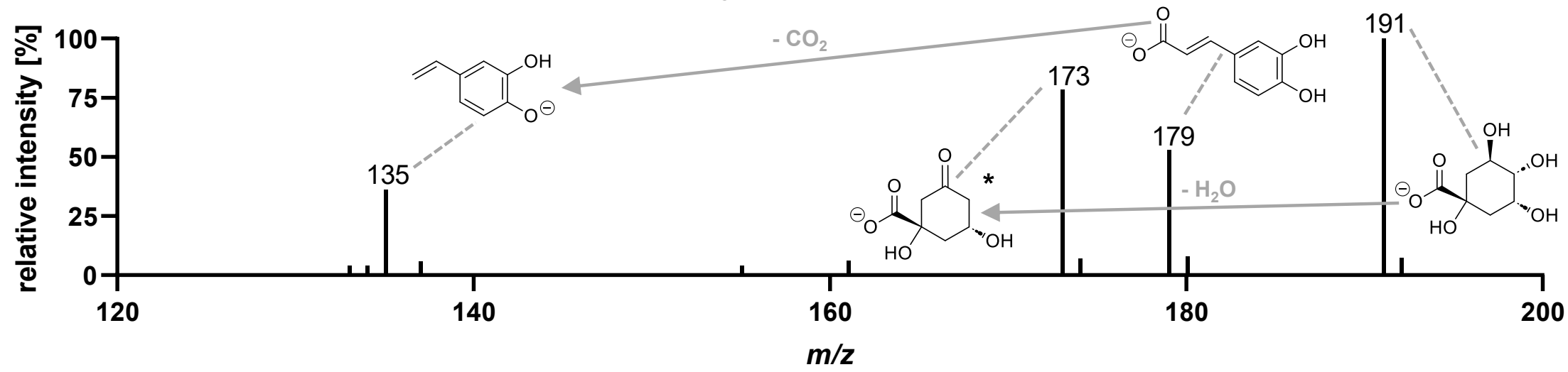

MS/MS caffeoyl-5-*O*-quinic acid reference

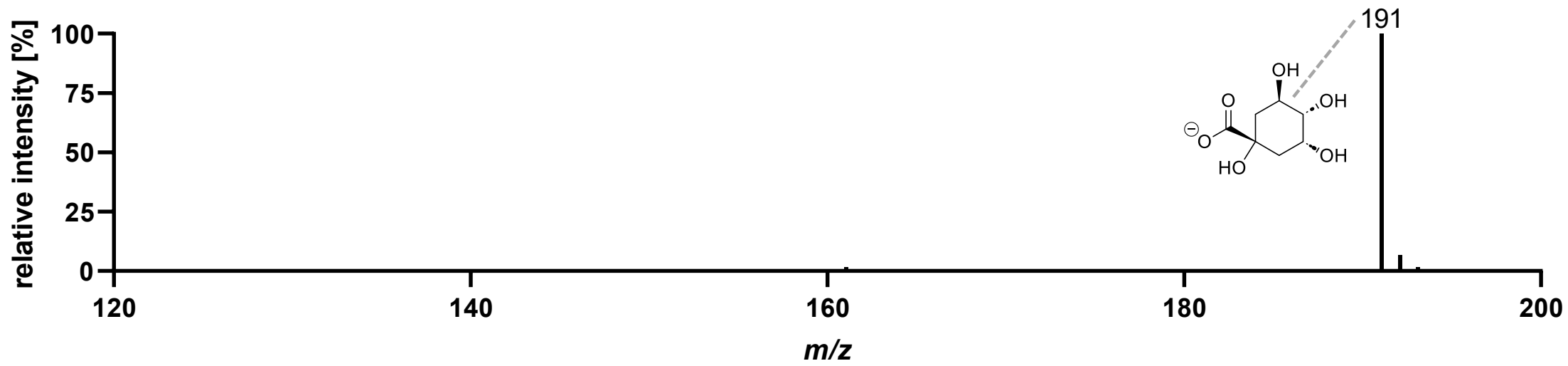

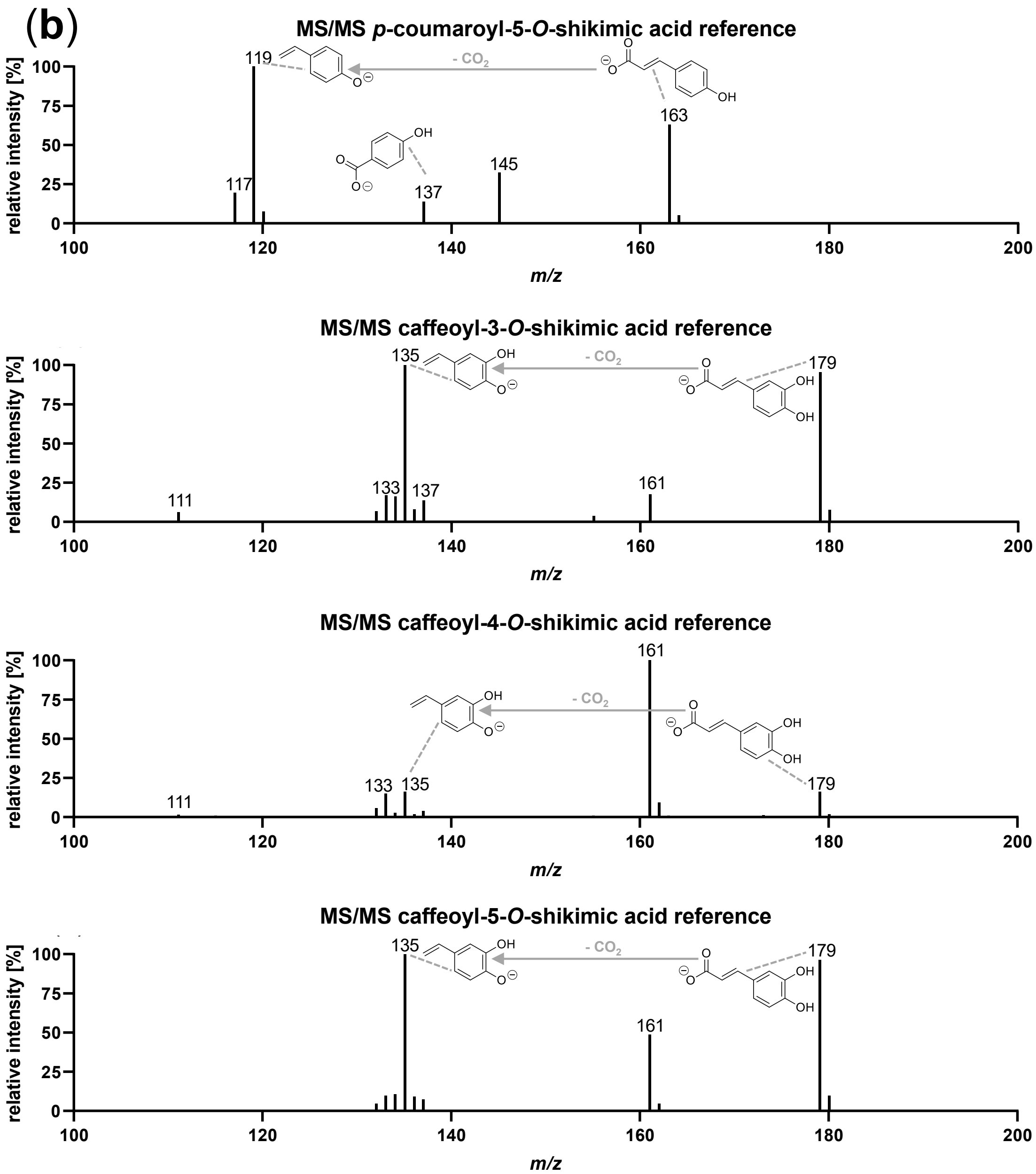
(b)
MS/MS p-coumaroyl-5-O-shikimic acid reference
relative intensity [%]
- CO2
119
117
137
145
163
OH
m/z
MS/MS caffeoyl-3-O-shikimic acid reference
135
179
111
133
137
161
MS/MS caffeoyl-4-O-shikimic acid reference
161
133
135
179
111
MS/MS caffeoyl-5-O-shikimic acid reference
135
179
161
100
75
50
25
0
120
140
160
180
200

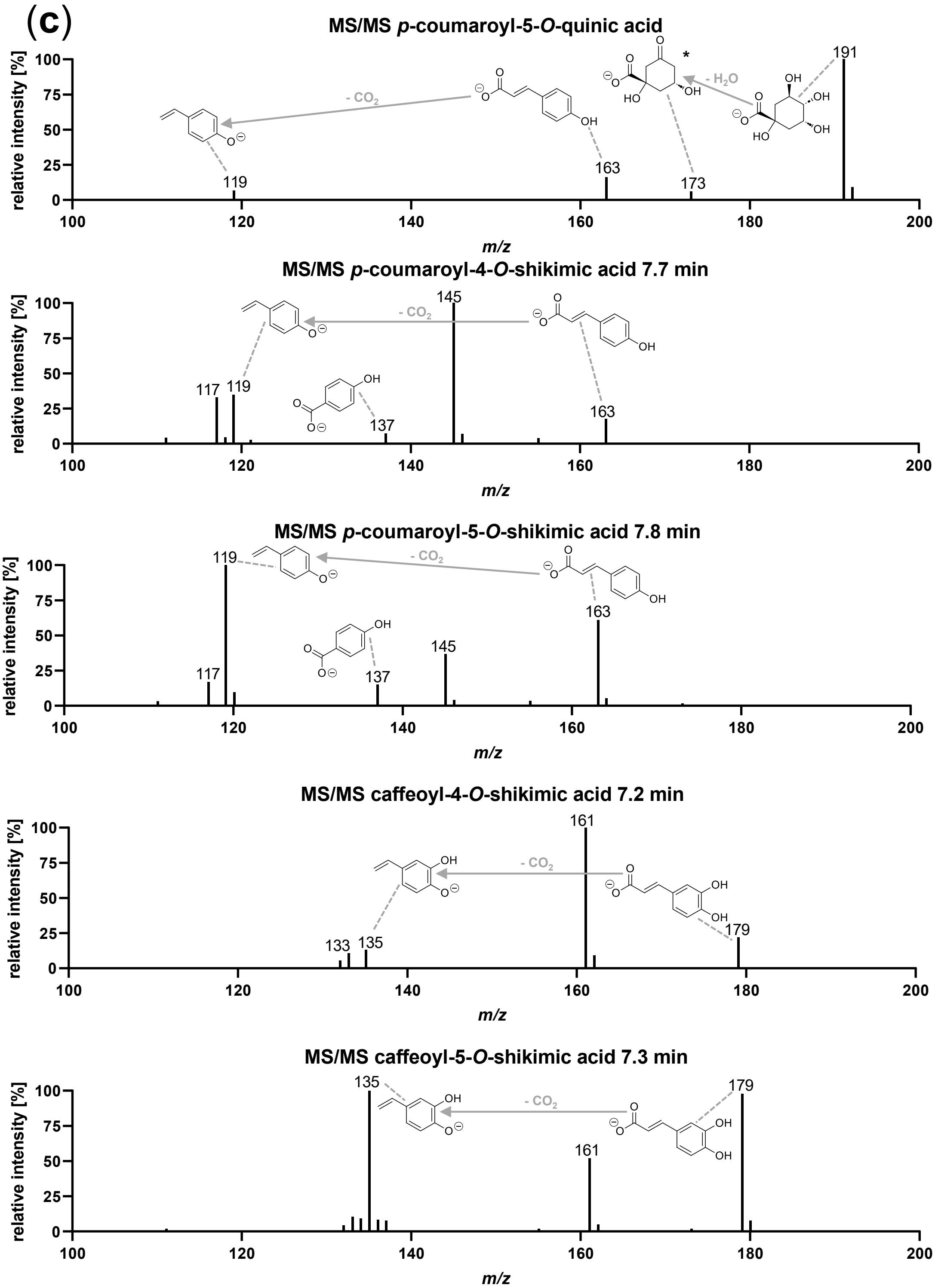
(c)
MS/MS p-coumaroyl-5-O-quinic acid
relative intensity [%]
m/z
- CO2
- H2O
119
163
173
191
MS/MS p-coumaroyl-4-O-shikimic acid 7.7 min
117
119
137
145
163
MS/MS p-coumaroyl-5-O-shikimic acid 7.8 min
117
119
137
145
163
MS/MS caffeoyl-4-O-shikimic acid 7.2 min
133
135
161
179
MS/MS caffeoyl-5-O-shikimic acid 7.3 min
135
161
179

(d)

MS/MS *p*-coumaroyl-5-*O*-quinic acid

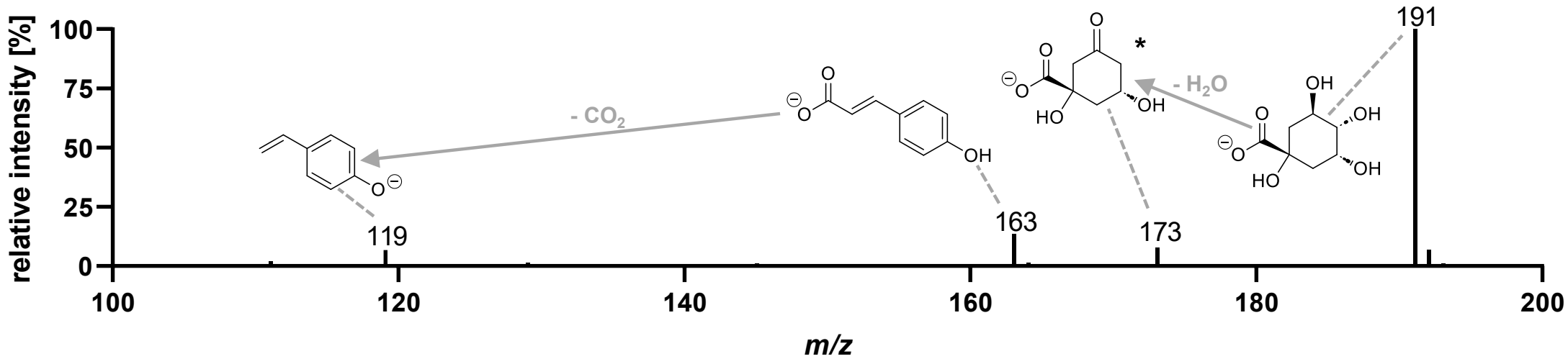

MS/MS caffeoyl-5-*O*-quinic acid

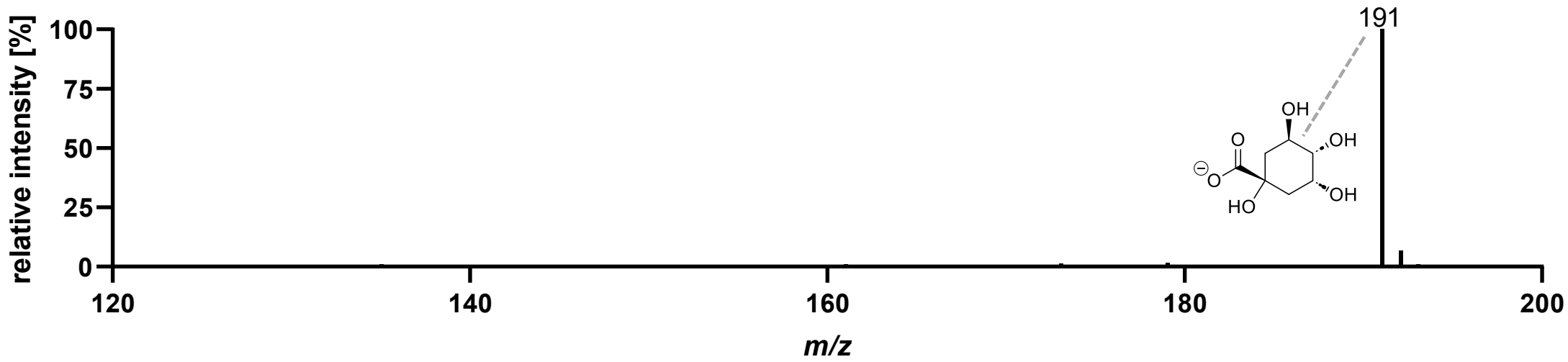

(e)

MS/MS *p*-coumaroyl-5-*O*-quinic acid

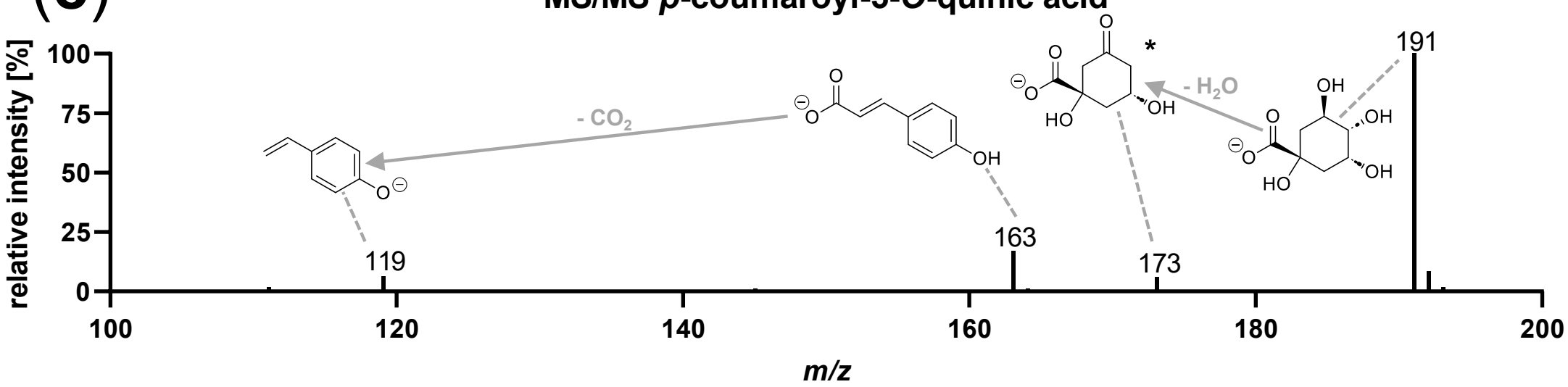

MS/MS caffeoyl-5-*O*-quinic acid

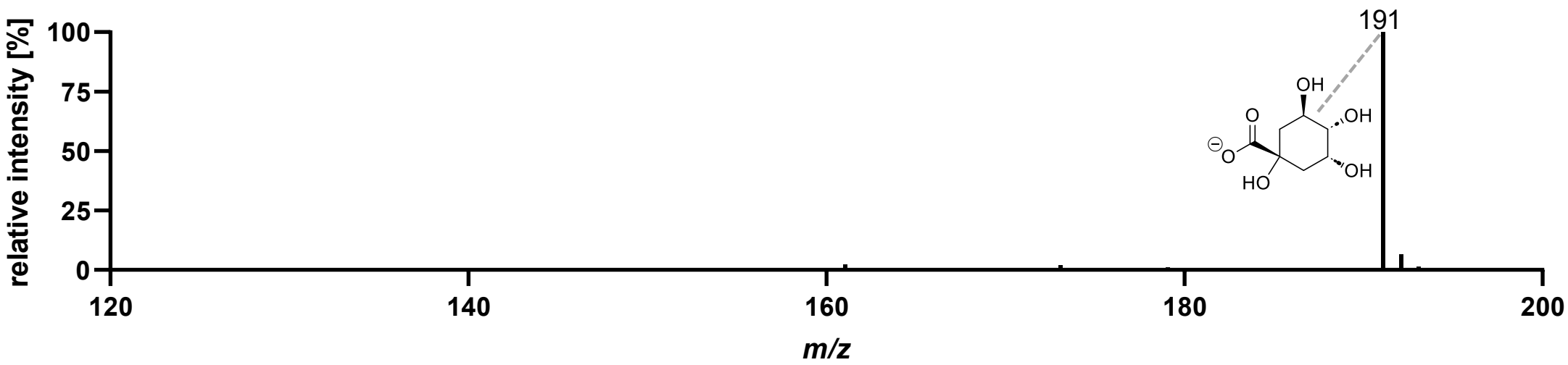

MS/MS *p*-coumaroyl-4-*O*-shikimic acid 7.6 min

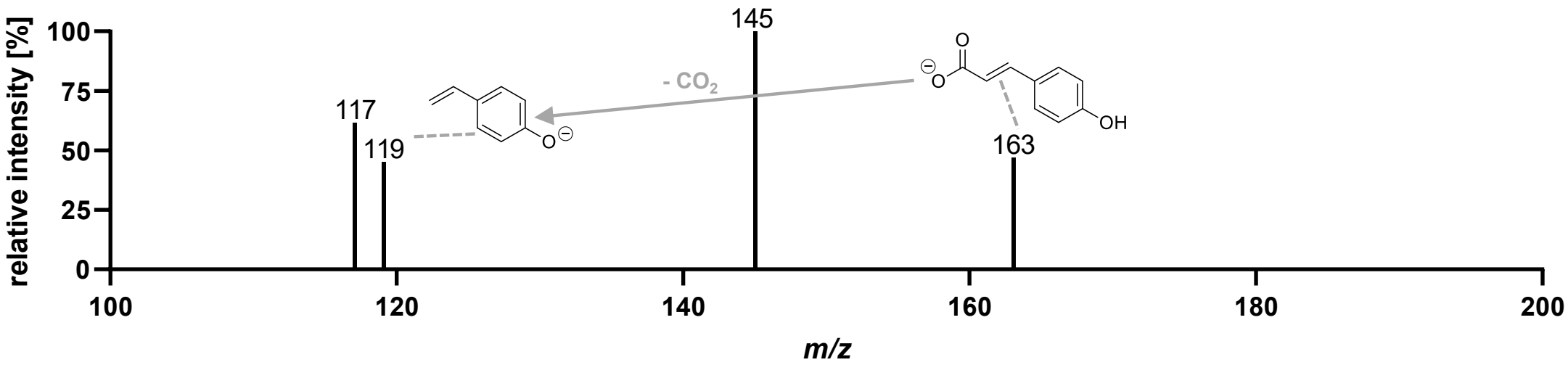

MS/MS *p*-coumaroyl-5-*O*-shikimic acid 7.7 min

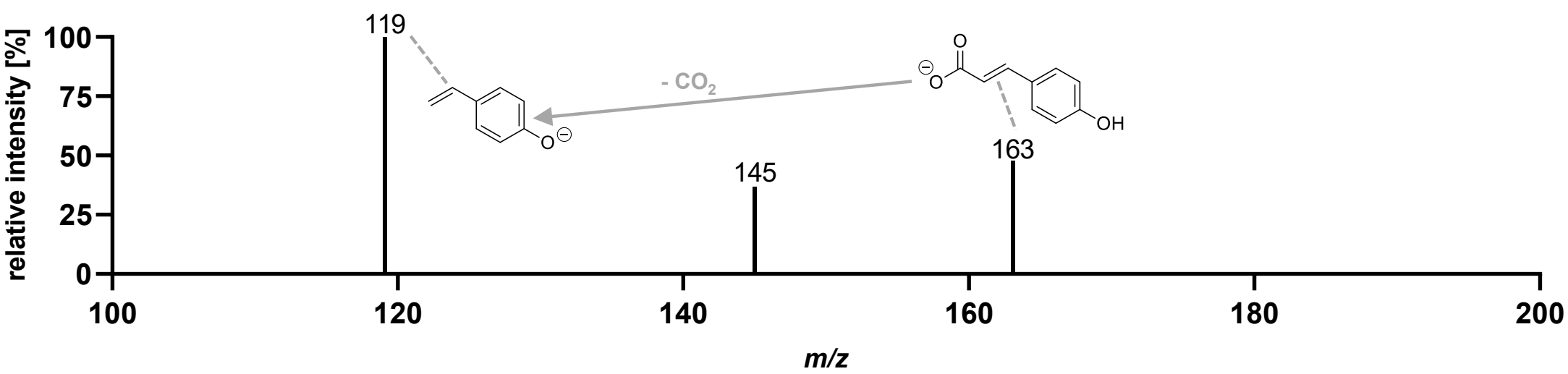

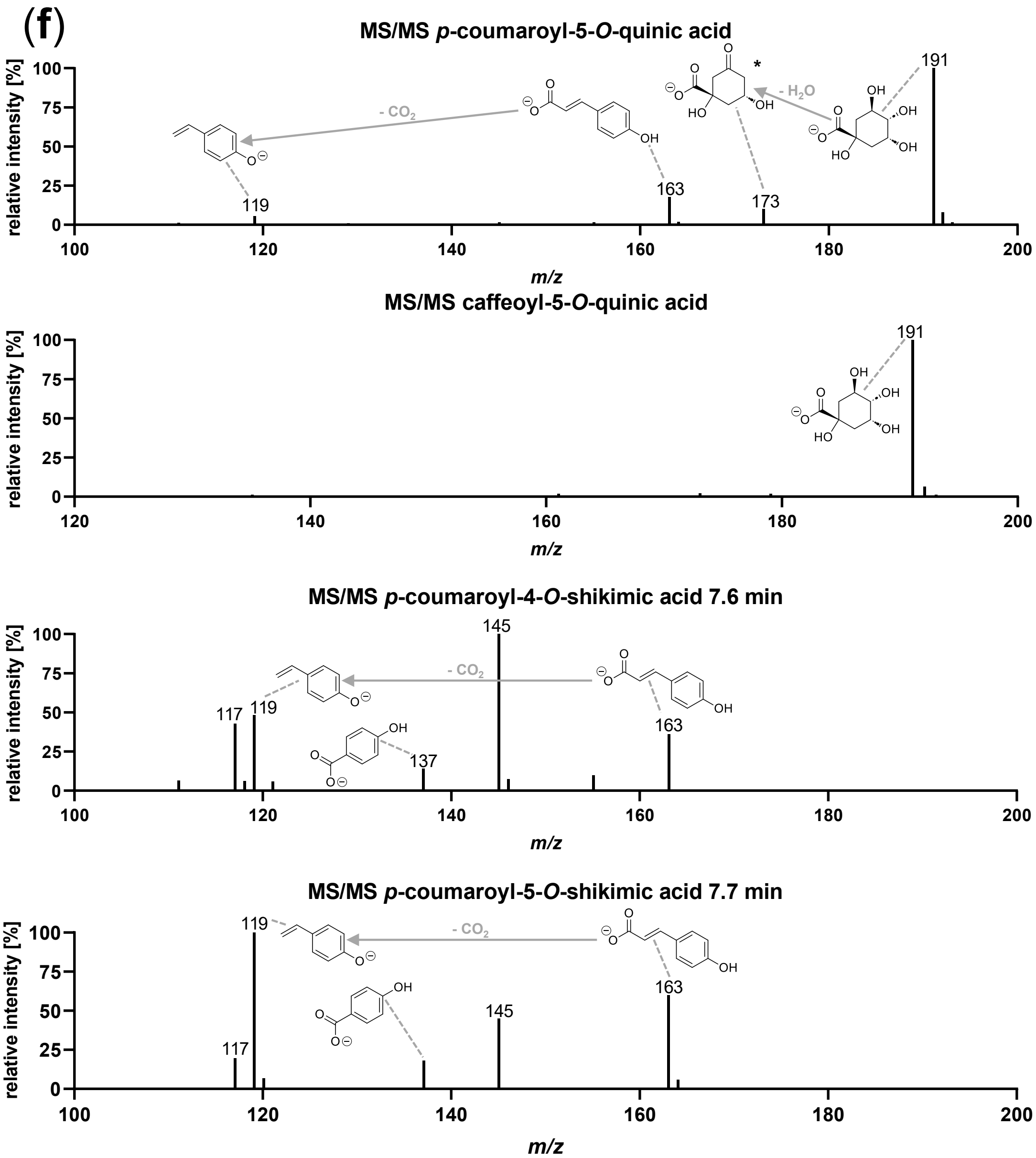

Fig. S2 MS/MS fragmentation of 3/4/5-*O*-shikimic/quinic acid esters. The water loss (*) shown at fragment 173 is only one of several possibilities. If no MS/MS event occurred, the retention time was used to determine the regio isomer (Tables S7-S14). Note, that the 4/5-*O* derivatives elute with almost identical retention times (4-*O* earlier than 5-*O*) and the 3-*O* derivative much earlier for quinic acid or much later for shikimic acid derivatives. (**a**) MS/MS fragmentation of *p*-coumaroyl/caffeoyl-3/4/5-*O*-quinic acid ester reference substances, (**b**) MS/MS fragmentation of *p*-coumaroyl-5-*O*-shikimic acid and caffeoyl-3/4/5-*O*-shikimic acid ester reference substances, (**c**) MS/MS fragmentation of quinic and shikimic acid ester derivatives formed by NbHCT10, (**d**) MS/MS fragmentation of quinic and shikimic acid ester derivatives formed by NbHCT11, (**e**) MS/MS fragmentation of quinic and shikimic acid ester derivatives formed by NbHCT12, (**f**) MS/MS fragmentation of quinic and shikimic acid ester derivatives formed by NbHCT13

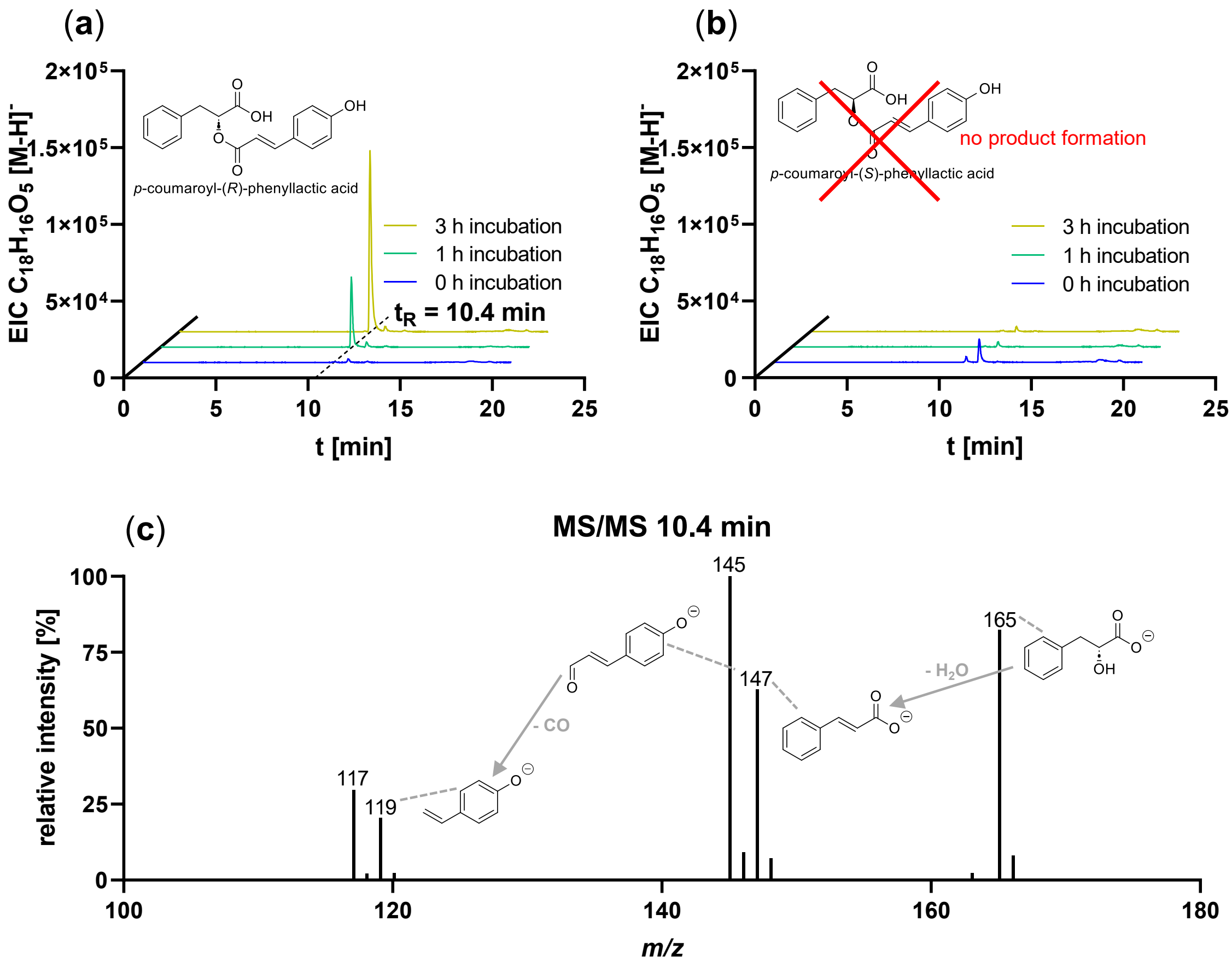

Fig. S3 Extracted ion chromatograms of enzyme assays of NbHCT21 after 0 h (blue), 1 h (green) and 3 h (yellow) of incubation with *p*-coumaroyl-CoA and (**a**) (*R*)-phenyllactic acid, (**b**) (*S*)-phenyllactic acid. (**c**) MS/MS fragmentation event of the product eluted at 10.4 min from the enzyme assays after incubation with *p*-coumaroyl-CoA and (*R*)-phenyllactic acid shown in (**a**)

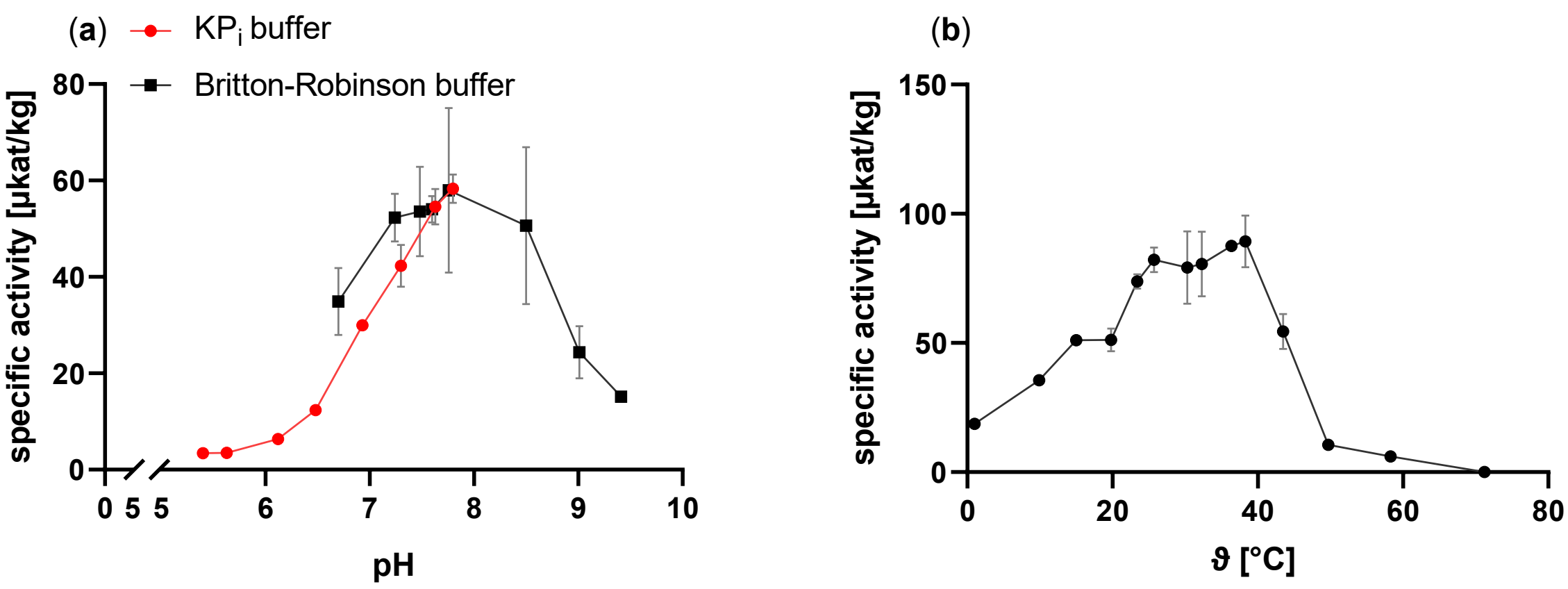

Fig. S4 pH and temperature optimum of NbHCT21. (**a**) pH optimum of NbHCT21 for the formation of *p*-coumaroyl-(*R*)-phenyllactic acid recorded in KP_i buffer (red line) and Britton-Robinson buffer (black line), (**b**) temperature optimum of NbHCT21 for the formation of *p*-coumaroyl-(*R*)-phenyllactic acid

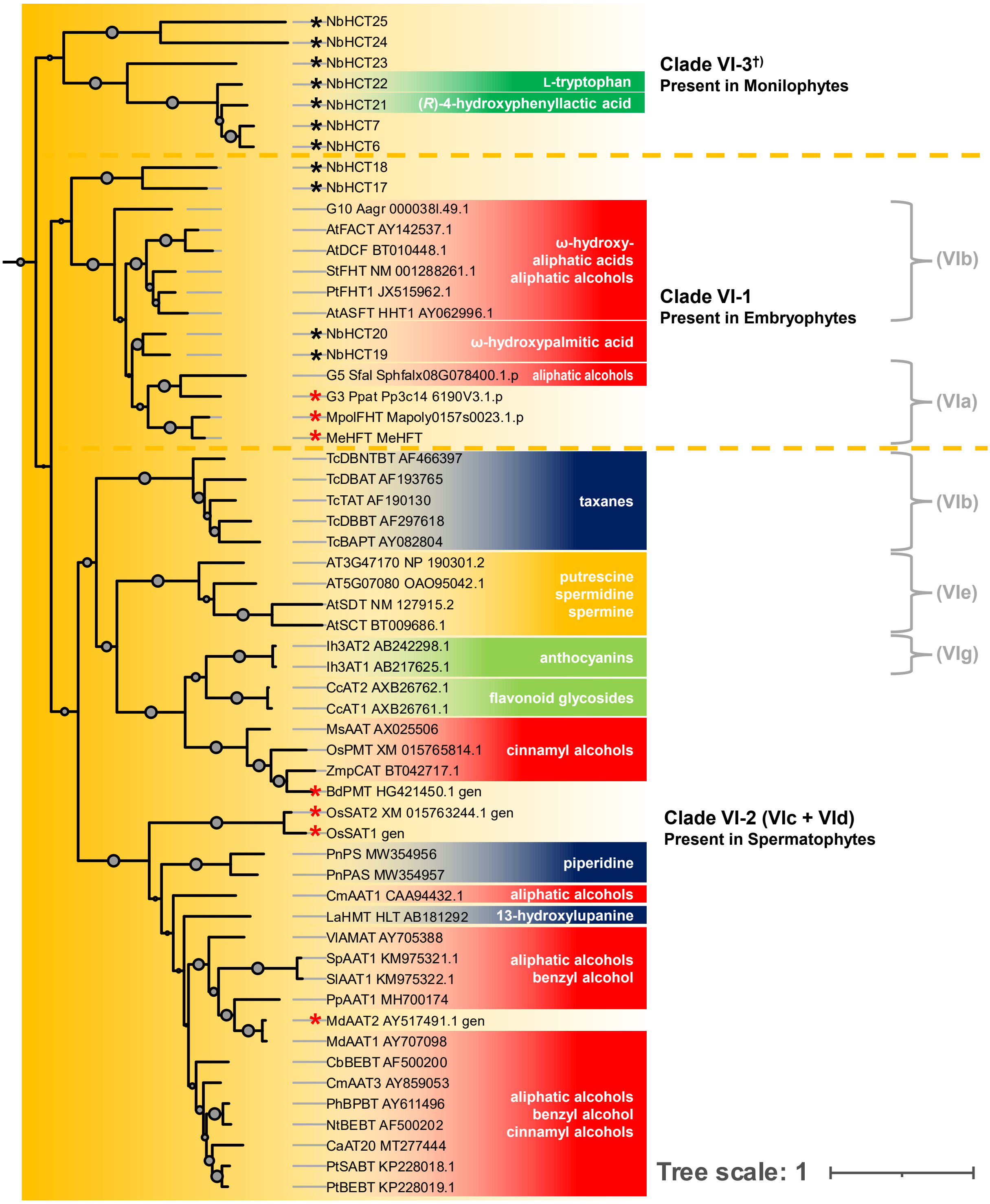

Fig. S5 Phylogenetic tree of clade VI of the tree shown in Fig. 3. The bootstrap support is shown by scaled grey circles. Sequences from *N. brasiliense* are marked with a black asterisk. Non-*N. brasiliense* sequences without known substrates are marked with a red asterisk. Acyl acceptor activity is colour coded: ■ shikimic acid and its derivatives, ■ quinic acid, ■ triterpenoids, ■ simple alcohols or amines with a carboxyl group, ■ flavonoid glycosides, ■ sugars, ■ other amines, ■ other alcohols, ■ other acceptors (e.g. alkaloids). Subclades as described by Kruse et al. (2022)(shown in brackets) and their orthogroup membership are summarized in new subclades. †) New lineage-specific subclade suggested by this work

(a)

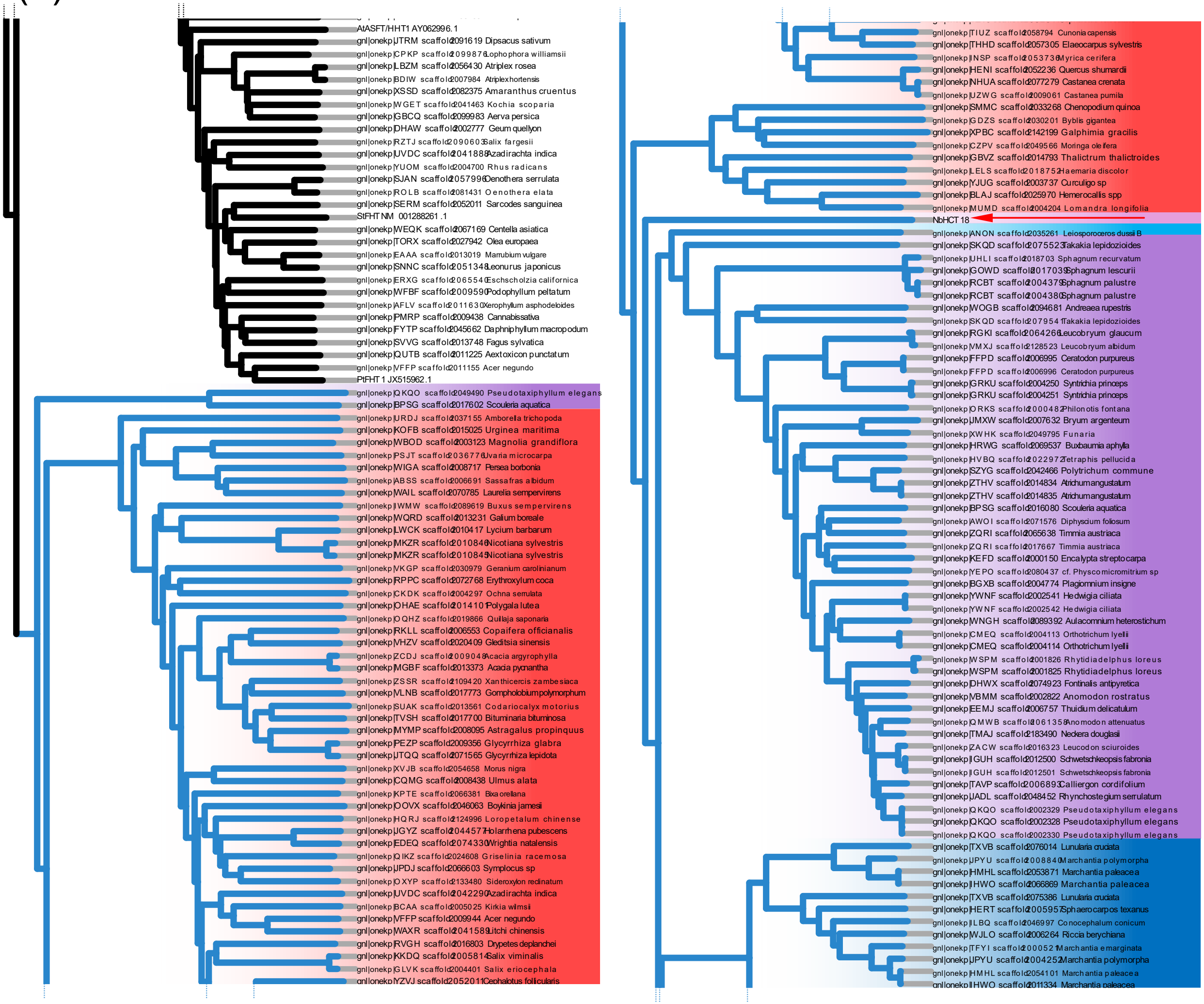

AtASFT/HHT1 AY062996.1
gnl|onekp|JTRM scaffold2091619 Dipsacus sativum
gnl|onekp|CPKP scaffold2099876 Lophophora williamsii
gnl|onekp|LBZM scaffold2056430 Atriplex rosea
gnl|onekp|BDIW scaffold2007984 Atriplex hortensis
gnl|onekp|XSSD scaffold2082375 Amaranthus cruentus
gnl|onekp|WGET scaffold2041463 Kochia scoparia
gnl|onekp|GBCQ scaffold2099983 Aerva persica
gnl|onekp|DHAW scaffold2002777 Geum quellyon
gnl|onekp|RZTJ scaffold2090603 Salix fargesii
gnl|onekp|UVDC scaffold2041888 Azadirachta indica
gnl|onekp|YUOM scaffold2004700 Rhus radicans
gnl|onekp|SJAN scaffold2057996 Oenothera serrulata
gnl|onekp|ROLB scaffold2081431 Oenothera elata
gnl|onekp|SERM scaffold2052011 Sarcodes sanguinea
StFHT NM 001288261.1
gnl|onekp|WEQK scaffold2067169 Centella asiatica
gnl|onekp|TORX scaffold2027942 Olea europaea
gnl|onekp|EAAA scaffold2013019 Marrubium vulgare
gnl|onekp|SNNC scaffold2051348 Leonurus japonicus
gnl|onekp|ERXG scaffold2065540 Eschscholzia californica
gnl|onekp|WFBF scaffold2009590 Podophyllum peltatum
gnl|onekp|AFLV scaffold2011630 Xerophyllum asphodeloides
gnl|onekp|PMRP scaffold2009438 Cannabis sativa
gnl|onekp|FYTP scaffold2045662 Daphniphyllum macropodum
gnl|onekp|SVVG scaffold2013748 Fagus sylvatica
gnl|onekp|QUTB scaffold2011225 Aextoxicon punctatum
gnl|onekp|VFFP scaffold2011155 Acer negundo
PtFHT1 JX515962.1
gnl|onekp|QKQO scaffold2049490 Pseudotaxiphyllum elegans
gnl|onekp|BPSG scaffold2017602 Scouleria aquatica
gnl|onekp|URDJ scaffold2037155 Amborella trichopoda
gnl|onekp|KOFB scaffold2015025 Urginea maritima
gnl|onekp|WBOD scaffold2003123 Magnolia grandiflora
gnl|onekp|PSJT scaffold2036776 Uvaria microcarpa
gnl|onekp|WIGA scaffold2008717 Persea borbonia
gnl|onekp|ABSS scaffold2006691 Sassafras albidum
gnl|onekp|WAIL scaffold2070785 Laurelia sempervirens
gnl|onekp|WMW scaffold2089619 Buxus sempervirens
gnl|onekp|WQRD scaffold2013231 Galium boreale
gnl|onekp|LWCK scaffold2010417 Lycium barbarum
gnl|onekp|MKZR scaffold2010846 Nicotiana sylvestris
gnl|onekp|MKZR scaffold2010845 Nicotiana sylvestris
gnl|onekp|VKGP scaffold2030979 Geranium carolinianum
gnl|onekp|RPPC scaffold2072768 Erythroxylum coca
gnl|onekp|CKDK scaffold2004297 Ochna serrulata
gnl|onekp|OHAE scaffold2014101 Polygala lutea
gnl|onekp|OQHZ scaffold2019866 Quillaja saponaria
gnl|onekp|RKLL scaffold2006553 Copaifera officianalis
gnl|onekp|VHZV scaffold2020409 Gleditsia sinensis
gnl|onekp|ZCDJ scaffold2009048 Acacia argyrophylla
gnl|onekp|MGBF scaffold2013373 Acacia pycnantha
gnl|onekp|ZSSR scaffold2109420 Xanthocercis zambesiaca
gnl|onekp|LNB scaffold2017773 Gompholobium polymorphum
gnl|onekp|SUAK scaffold2013561 Codariocalyx motorius
gnl|onekp|TVSH scaffold2017700 Bituminaria bituminosa
gnl|onekp|MYMP scaffold2008095 Astragalus propinquus
gnl|onekp|PEZP scaffold2009356 Glycyrrhiza glabra
gnl|onekp|JTQQ scaffold2071565 Glycyrrhiza lepidota
gnl|onekp|XVJB scaffold2054658 Morus nigra
gnl|onekp|CQMG scaffold2008438 Ulmus alata
gnl|onekp|KPTE scaffold2066381 Bixa orellana
gnl|onekp|OOVX scaffold2046063 Boykinia jamesii
gnl|onekp|HQRJ scaffold2124996 Loropetalum chinense
gnl|onekp|GYZ scaffold2044571 Holarrhena pubescens
gnl|onekp|EDEQ scaffold2074330 Wrightia natalensis
gnl|onekp|QIKZ scaffold2024608 Griselinia racemosa
gnl|onekp|JPDJ scaffold2066603 Symplocus sp
gnl|onekp|OXYP scaffold2133480 Sideroxylon reclinatum
gnl|onekp|UVDC scaffold2042290 Azadirachta indica
gnl|onekp|BCAA scaffold2005025 Kirkia wilmsii
gnl|onekp|VFFP scaffold2009944 Acer negundo
gnl|onekp|WAXR scaffold2041589 Litchi chinensis
gnl|onekp|RVGH scaffold2016803 Drypetes deplanchei
gnl|onekp|KKDQ scaffold2005814 Salix viminalis
gnl|onekp|GLVK scaffold2004401 Salix eriocephala
gnl|onekp|YZVJ scaffold2052011 Cephalotus follicularis
gnl|onekp|TIUZ scaffold2058794 Cunonia capensis
gnl|onekp|THHD scaffold2057305 Elaeocarpus sylvestris
gnl|onekp|NSP scaffold2053736 Myrica cerifera
gnl|onekp|HENI scaffold2052236 Quercus shumardii
gnl|onekp|NHUA scaffold2077279 Castanea crenata
gnl|onekp|UZWG scaffold2009061 Castanea pumila
gnl|onekp|SMMC scaffold2033268 Chenopodium quinoa
gnl|onekp|GDZS scaffold2030201 Byblis gigantea
gnl|onekp|XPBC scaffold2142199 Galphimia gracilis
gnl|onekp|CZPV scaffold2049566 Moringa oleifera
gnl|onekp|GBVZ scaffold2014793 Thalictrum thalictroides
gnl|onekp|LELS scaffold2018752 Haemaria discolor
gnl|onekp|YJUG scaffold2003737 Curculigo sp
gnl|onekp|BLAJ scaffold2025970 Hemerocallis spp
gnl|onekp|MUMD scaffold2004204 Lomandra longifolia
NbHCT18
gnl|onekp|ANON scaffold2035261 Leiosporoceros dussii B
gnl|onekp|SKQD scaffold2075523 Takakia lepidozioides
gnl|onekp|UHLI scaffold2018703 Sphagnum recurvatum
gnl|onekp|GOWD scaffold2017039 Sphagnum lescurii
gnl|onekp|RCBT scaffold2004379 Sphagnum palustre
gnl|onekp|RCBT scaffold2004380 Sphagnum palustre
gnl|onekp|WOGB scaffold2094681 Andreaea rupestris
gnl|onekp|SKQD scaffold2077954 Takakia lepidozioides
gnl|onekp|RGKI scaffold2064266 Leucobryum glaucum
gnl|onekp|VMXJ scaffold2128523 Leucobryum albidum
gnl|onekp|FFPD scaffold2006995 Ceratodon purpureus
gnl|onekp|FFPD scaffold2006996 Ceratodon purpureus
gnl|onekp|GRKU scaffold2004250 Syntrichia princeps
gnl|onekp|GRKU scaffold2004251 Syntrichia princeps
gnl|onekp|ORKS scaffold2000482 Philonotis fontana
gnl|onekp|JMXW scaffold2007632 Bryum argenteum
gnl|onekp|XWHK scaffold2049795 Funaria
gnl|onekp|HRWG scaffold2069537 Buxbaumia aphylla
gnl|onekp|HVBQ scaffold2022972 Tetraphis pellucida
gnl|onekp|SZYG scaffold2042466 Polytrichum commune
gnl|onekp|ZTHV scaffold2014834 Atrichum angustatum
gnl|onekp|ZTHV scaffold2014835 Atrichum angustatum
gnl|onekp|BPSG scaffold2016080 Scouleria aquatica
gnl|onekp|AWOI scaffold2071576 Diphyscium foliosum
gnl|onekp|ZQRI scaffold2065638 Timmia austriaca
gnl|onekp|ZQRI scaffold2017667 Timmia austriaca
gnl|onekp|KEFD scaffold2000150 Encalypta streptocarpa
gnl|onekp|YEPO scaffold2080437 cf. Physcomicromitrium sp
gnl|onekp|BGXB scaffold2004774 Plagiomnium insigne
gnl|onekp|YWNF scaffold2002541 Hedwigia ciliata
gnl|onekp|YWNF scaffold2002542 Hedwigia ciliata
gnl|onekp|WNGH scaffold2089392 Aulacomnium heterostichum
gnl|onekp|CMEQ scaffold2004113 Orthotrichum lyellii
gnl|onekp|CMEQ scaffold2004114 Orthotrichum lyellii
gnl|onekp|WSPM scaffold2001826 Rhytidiadelphus loreus
gnl|onekp|WSPM scaffold2001825 Rhytidiadelphus loreus
gnl|onekp|DHWX scaffold2074923 Fontinalis antipyretica
gnl|onekp|VBMM scaffold2002822 Anomodon rostratus
gnl|onekp|EEMJ scaffold2006757 Thuidium delicatulum
gnl|onekp|QMWB scaffold2061358 Anomodon attenuatus
gnl|onekp|TMAJ scaffold2183490 Neckera douglasii
gnl|onekp|ZACW scaffold2016323 Leucodon sciuroides
gnl|onekp|IGUH scaffold2012500 Schwetschkeopsis fabronia
gnl|onekp|IGUH scaffold2012501 Schwetschkeopsis fabronia
gnl|onekp|TAVP scaffold2006893 Calliergon cordifolium
gnl|onekp|JADL scaffold2048452 Rhynchostegium serrulatum
gnl|onekp|QKQO scaffold2002329 Pseudotaxiphyllum elegans
gnl|onekp|QKQO scaffold2002328 Pseudotaxiphyllum elegans
gnl|onekp|QKQO scaffold2002330 Pseudotaxiphyllum elegans
gnl|onekp|TXVB scaffold2076014 Lunularia cruciata
gnl|onekp|JPYU scaffold2008840 Marchantia polymorpha
gnl|onekp|HMHL scaffold2053871 Marchantia paleacea
gnl|onekp|IHWO scaffold2066869 Marchantia paleacea
gnl|onekp|TXVB scaffold2075386 Lunularia cruciata
gnl|onekp|HERT scaffold2005957 Sphaerocarpos texanus
gnl|onekp|ILBQ scaffold2046997 Conocephalum conicum
gnl|onekp|WJLO scaffold2006264 Riccia berychiana
gnl|onekp|TFYI scaffold2000521 Marchantia emarginata
gnl|onekp|JPYU scaffold2004252 Marchantia polymorpha
gnl|onekp|HMHL scaffold2054101 Marchantia paleacea
gnl|onekp|IHWO scaffold2011334 Marchantia paleacea

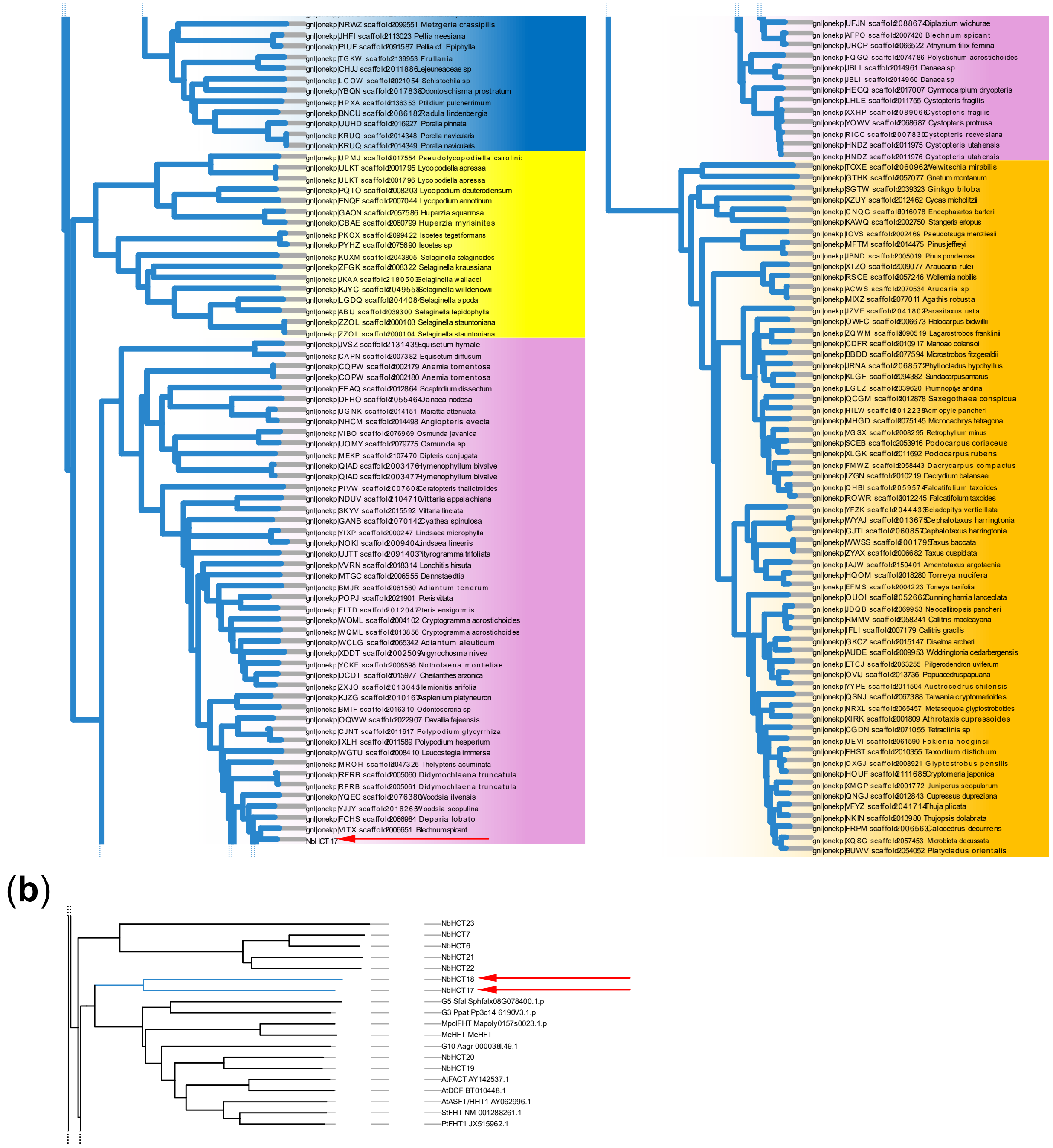

Fig. S6 Orthogroup analysis of NbHCT17 and NbHCT18. Excerpt of the final phylogenetic trees (indicated by the dotted lines). Blue branches indicate a monophyletic orthogroup. *Neoblechnum brasiliense* sequences are indicated by a red arrow. (**a**) Phylogenetic tree generated with all blastp results that are sorted into the orthogroup. Color code: ■ Angiosperms, ■ Gymnosperms, ■ Monilophytes (■ Member of the order Cyatheales, ■ Member of the order Polypodiales), ■ Lycophytes, ■ Bryophytes *sensu stricto*, ■ Anthocerotophytes, ■ Marchantiophytes. (**b**) Phylogenetic tree generated excluding embryophytes in the blastp searches

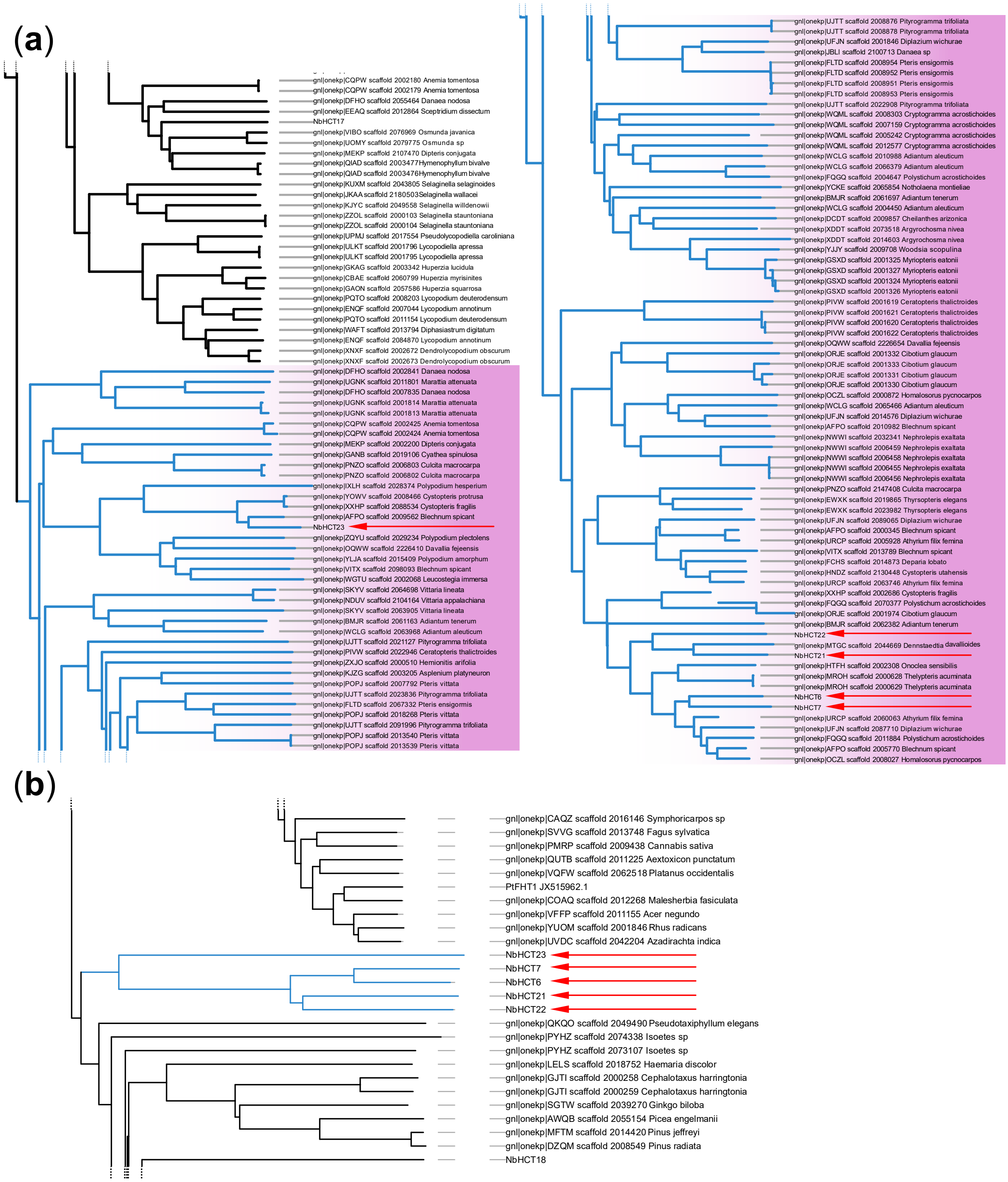

Fig. S7 Orthogroup analysis of NbHCT6, 7 and 21-23. Excerpt of the final phylogenetic trees (indicated by the dotted lines). Blue branches indicate a monophyletic orthogroup. *Neoblechnum brasiliense* sequences are indicated by a red arrow. (**a**) Phylogenetic tree generated with all blastp results that are sorted into the orthogroup. Color code: ■ Angiosperms, ■ Gymnosperms, ■ Monilophytes (■ Member of the order Cyatheales, ■ Member of the order Polypodiales), ■ Lycophytes, ■ Bryophytes *sensu stricto*, ■ Anthocerotophytes, ■ Marchantiophytes. (**b**) Phylogenetic tree generated excluding monilophytes in the blastp searches

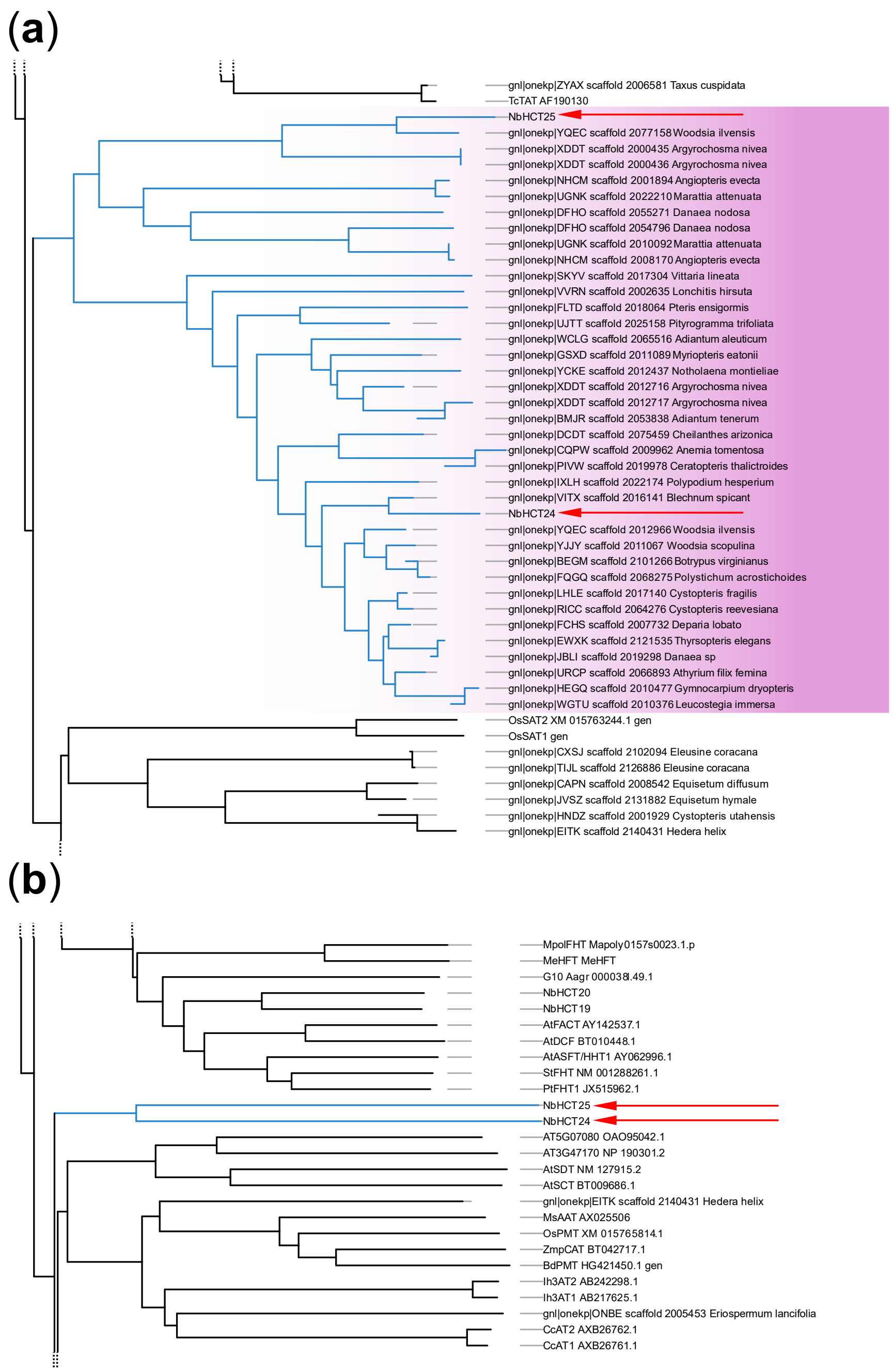

Fig. S8 Orthogroup analysis of NbHCT24 and 25. Excerpt of the final phylogenetic trees (indicated by the dotted lines). Blue branches indicate a monophyletic orthogroup. *Neoblechnum brasiliense* sequences are indicated by a red arrow. (**a**) Phylogenetic tree generated with all blastp results that are sorted into the orthogroup. Color code: ■ Angiosperms, ■ Gymnosperms, ■ Monilophytes (■ Member of the order Cyatheales, ■ Member of the order Polypodiales), ■ Lycophytes, ■ Bryophytes *sensu stricto*, ■ Anthocerotophytes, ■ Marchantiophytes. (**b**) Phylogenetic tree generated excluding monilophytes in the blastp searches

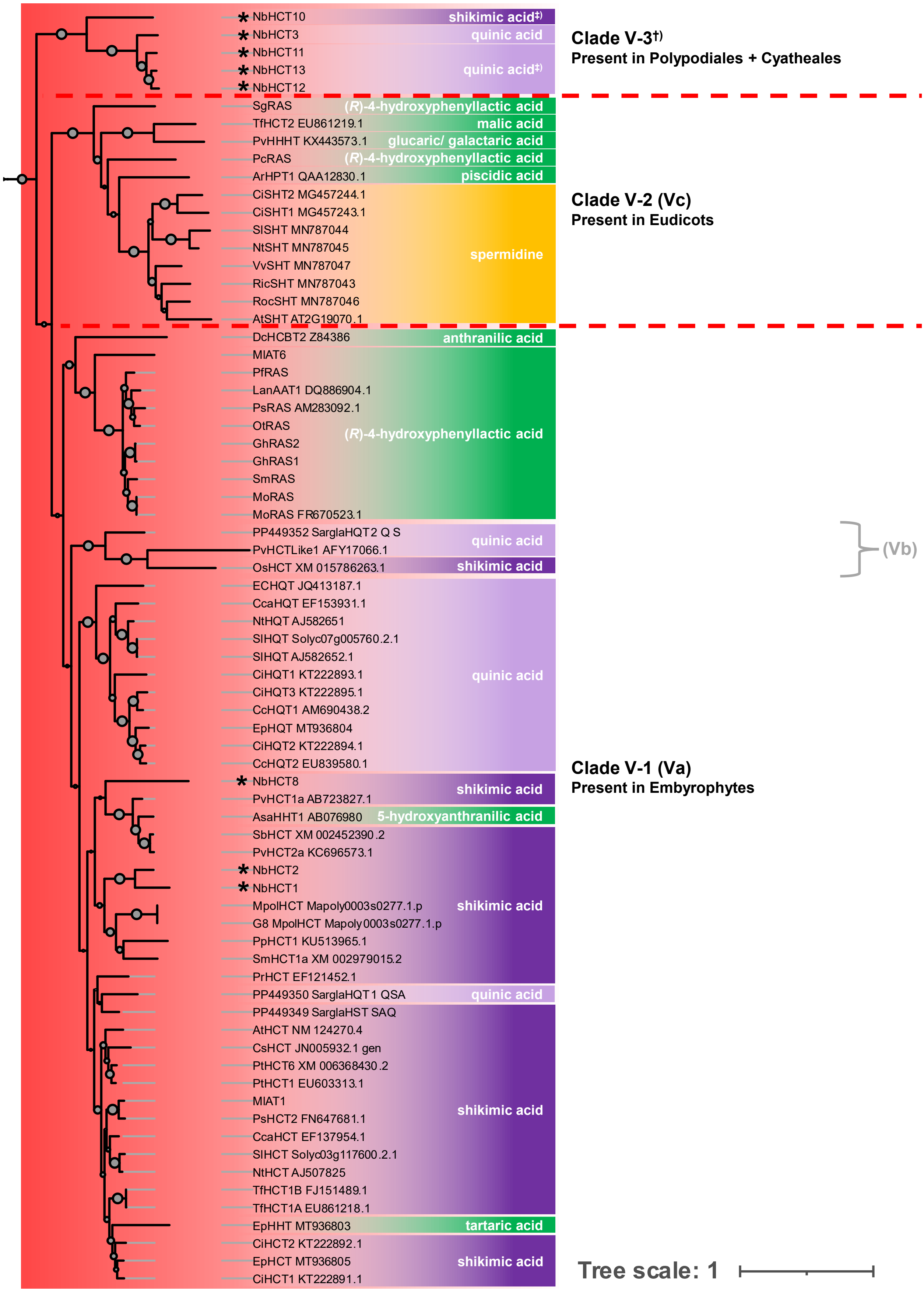

Fig. S9 Phylogenetic tree of clade V of the tree shown in Fig. 3. The bootstrap support is shown by scaled grey circles. Sequences from *N. brasiliense* are marked with a black asterisk. Acyl acceptor activity is colour coded: ■ shikimic acid and its derivatives, ■ quinic acid,

■ triterpenoids, ■ simple alcohols or amines with a carboxyl group, ■ flavonoid glycosides, ■ sugars, ■ other amines, ■ other alcohols, ■ other acceptors (e.g. alkaloids). Subclades as described by Kruse et al. (2022)(shown in brackets) and their orthogroup membership are summarized in new subclades. †) New lineage-specific subclade suggested by this work. ‡) preferred acyl acceptor only suggested by LC/ESI-MS/MS data in comparison to biochemically characterized *N. brasiliense* BAHDs

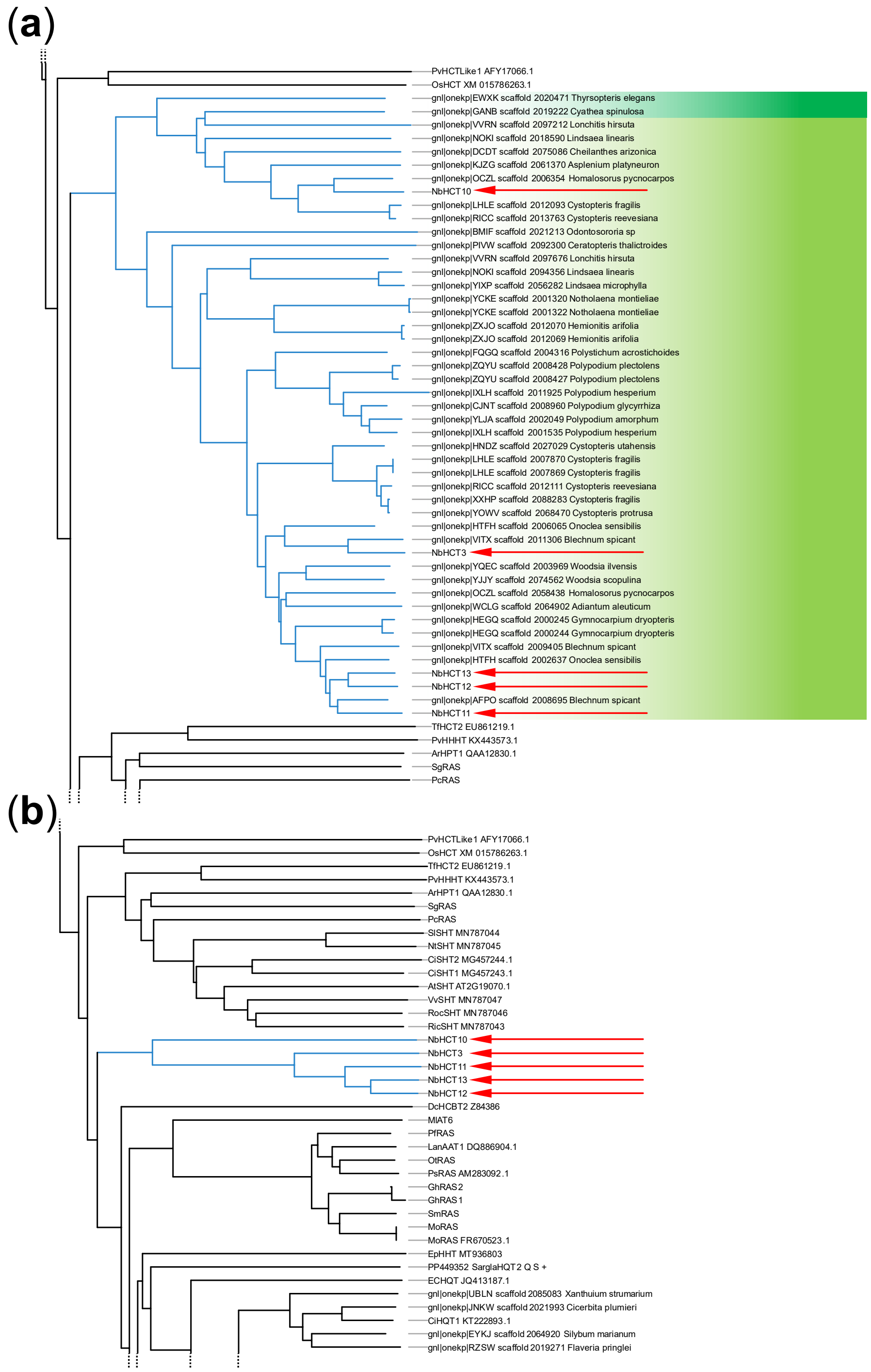

Fig. S10 Orthogroup analysis of NbHCT3 and NbHCT10-13. Excerpt of the final phylogenetic trees (indicated by the dotted lines). Blue branches indicate a monophyletic orthogroup. *Neoblechnum brasiliense* sequences are indicated by a red arrow. (**a**) Phylogenetic tree

generated with all blastp results that are sorted into the orthogroup. Color code: ■ Angiosperms, ■ Gymnosperms, ■ Monilophytes (■ Member of the order Cyatheales, ■ Member of the order Polypodiales), ■ Lycophytes, ■ Bryophytes *sensu stricto*, ■ Anthocerotophytes, ■ Marchantiophytes. (**b**) Phylogenetic tree generated excluding Cyatheales and Polypodiales in the blastp searches

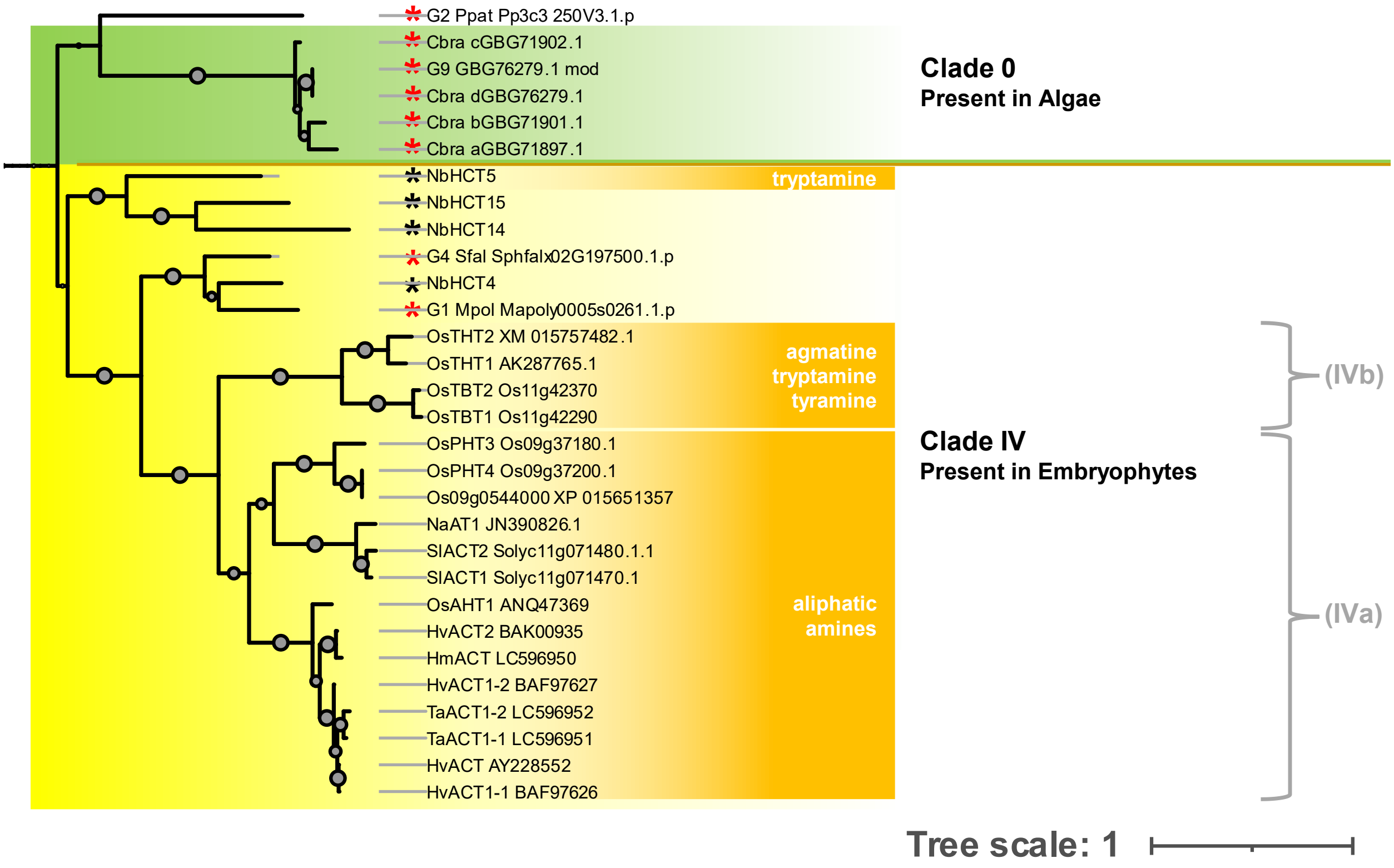

Fig. S11 Phylogenetic tree of clade IV and parts of clade I of the tree shown in Fig. 3. The bootstrap support is shown by scaled grey circles. Sequences from *N. brasiliense* are marked with a black asterisk. Non-*N. brasiliense* sequences without known substrates are marked with a red asterisk. Acyl acceptor activity is colour coded: ■ shikimic acid and its derivatives, ■ quinic acid, ■ triterpenoids, ■ simple alcohols or amines with a carboxyl group, ■ flavonoid glycosides, ■ sugars, ■ other amines, ■ other alcohols, ■ other acceptors (e.g. alkaloids). Subclades as described by Kruse et al. (2022) (shown in brackets) and their orthogroup membership are summarized in new subclades

(a)

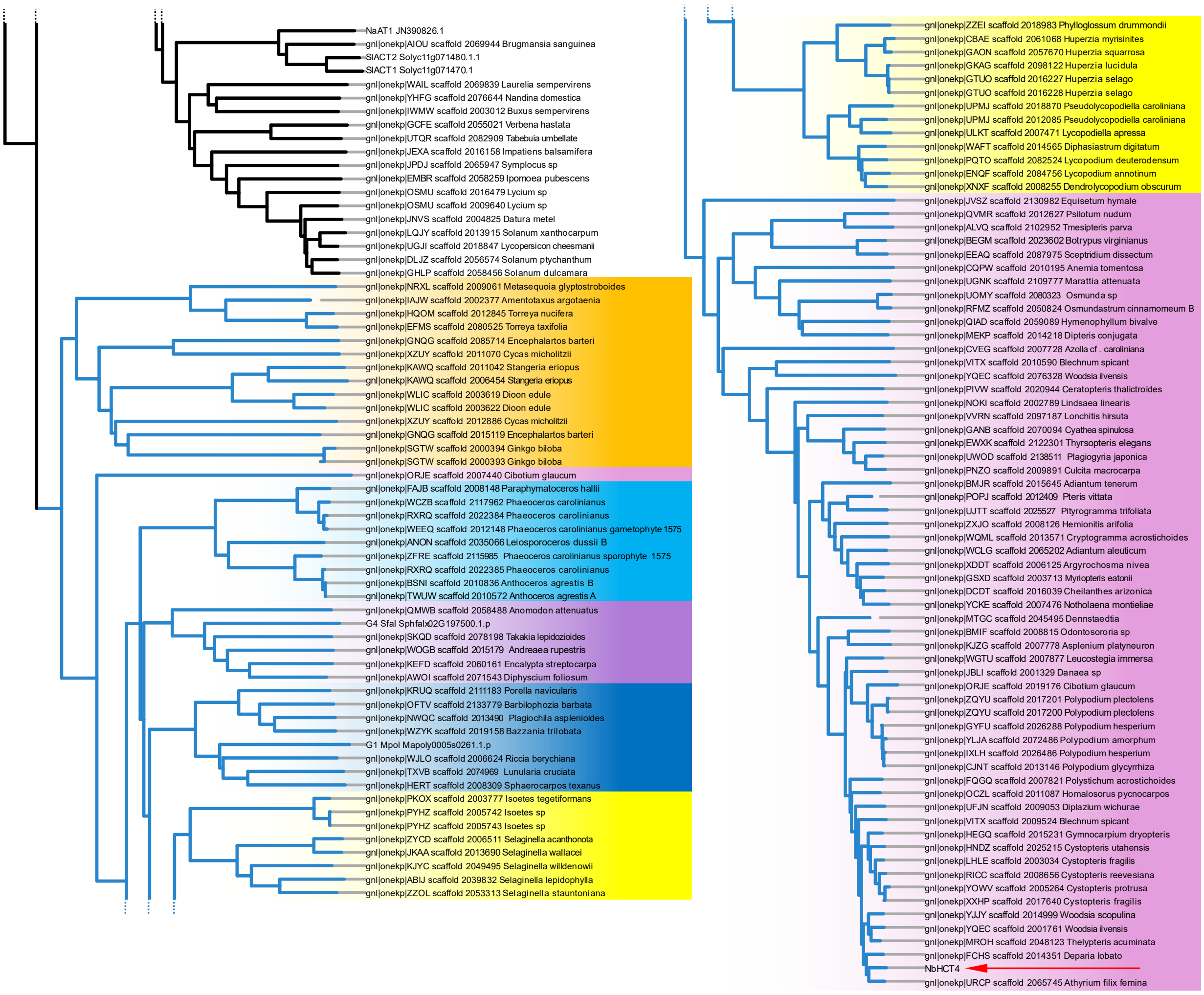

(b)

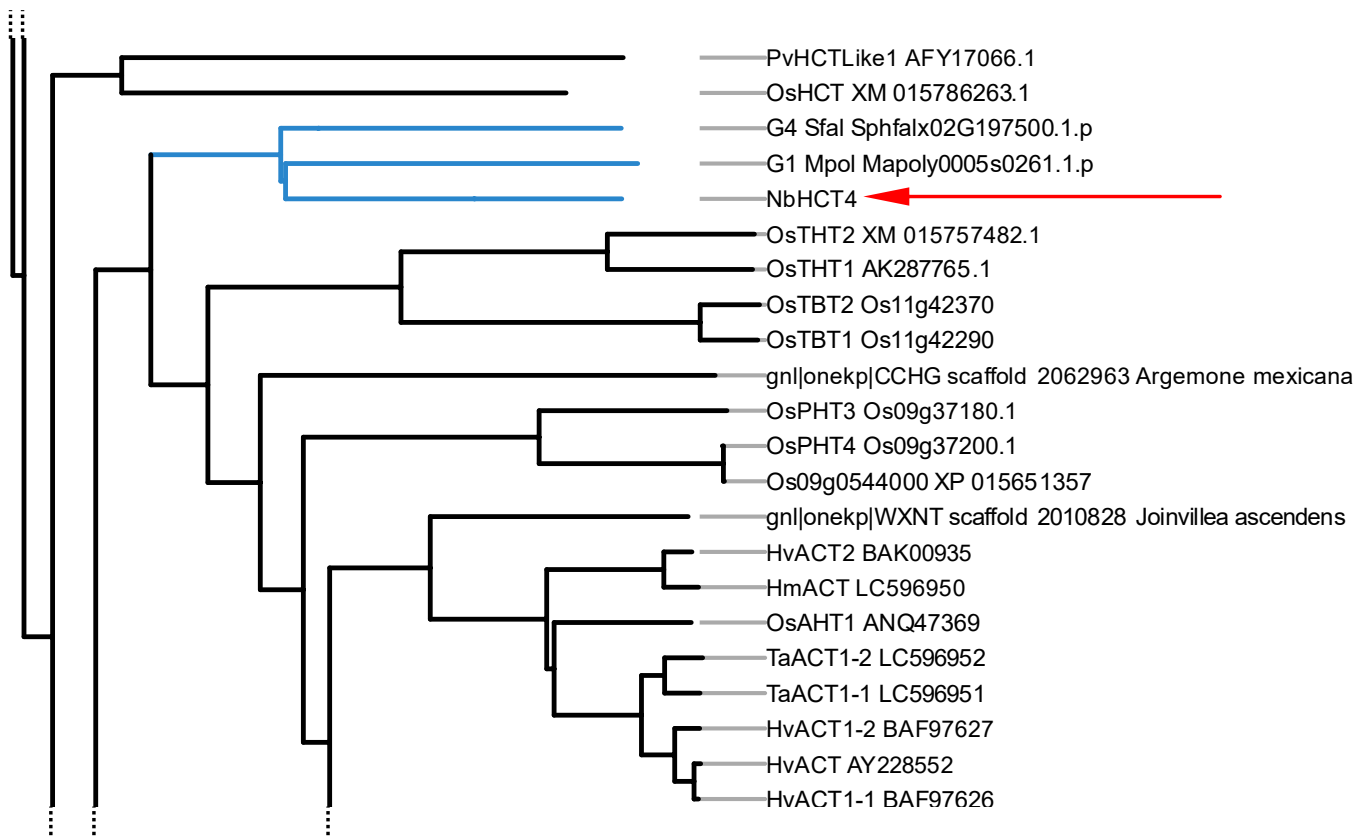

Fig. S12 Orthogroup analysis of NbHCT4, Mapoly0005s0261.1.p, Sphfalx02G197500.1.p. Excerpt of the final phylogenetic trees (indicated by the dotted lines). Blue branches indicate a monophyletic orthogroup. *Neoblechnum brasiliense* sequences are indicated by a red arrow. (**a**) Phylogenetic tree generated with all blastp results that are sorted into the orthogroup. Color code: ■ Angiosperms, ■ Gymnosperms, ■ Monilophytes (■ Member of the order Cyatheales, ■ Member of the order Polypodiales), ■ Lycophytes, ■ Bryophytes *sensu stricto*,

■ Anthocerotophytes, ■ Marchantiophytes. (**b**) Phylogenetic tree generated excluding non-seed plants and gymnosperms in the blastp searches

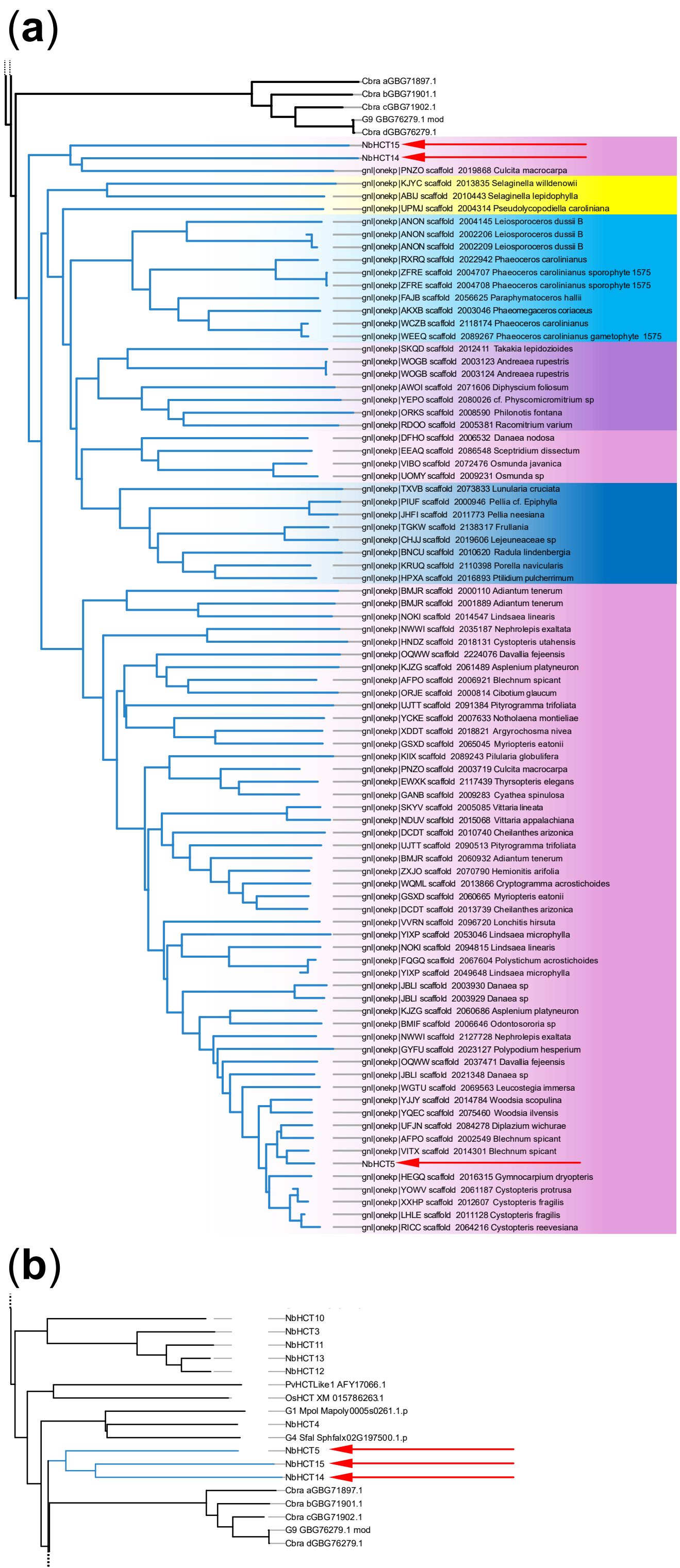

Fig. S13 Orthogroup analysis of NbHCT5, 14 and 15. Excerpt of the final phylogenetic trees (indicated by the dotted lines). Blue branches indicate a monophyletic orthogroup. *Neoblechnum brasiliense* sequences are indicated by a red arrow. (**a**) Phylogenetic tree generated with all blastp results that are sorted into the orthogroup. Color code: ■ Angiosperms, ■ Gymnosperms, ■ Monilophytes (■ Member of the order Cyatheales,

■ Member of the order Polypodiales), ■ Lycophytes, ■ Bryophytes *sensu stricto*, ■ Anthocerotophytes, ■ Marchantiophytes. (**b**) Phylogenetic tree generated excluding non-seed plants in the blastp searches

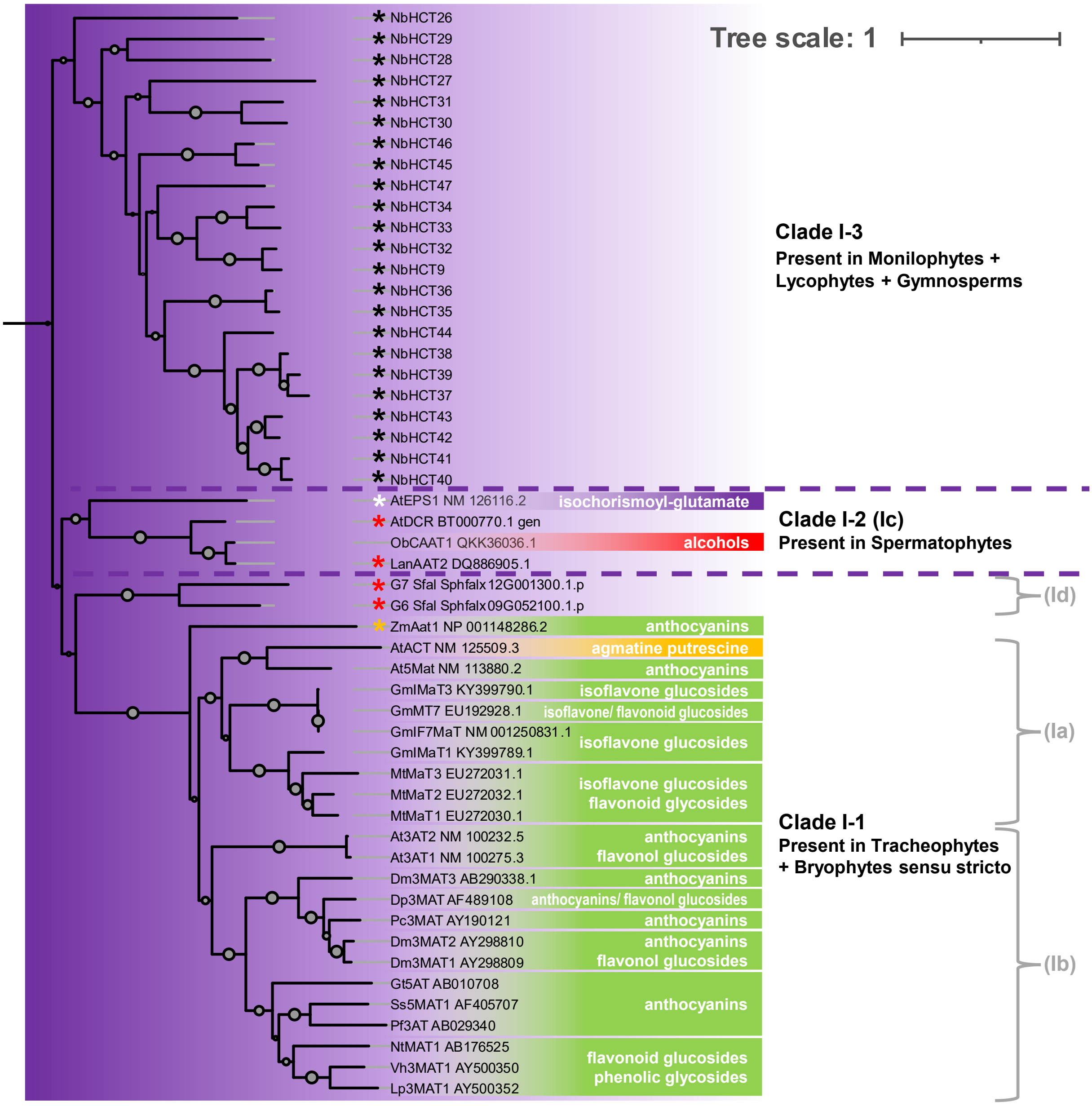

Fig. S14 Phylogenetic tree of clade I of the tree shown in Fig. 3. The bootstrap support is shown by scaled grey circles. Sequences from *N. brasiliense* are marked with a black asterisk. Non-*N. brasiliense* sequences without known substrates are marked with a red asterisk. The sequence with a white asterisk has an unusual lyase activity uncommon to the BAHD enzyme family. Acyl acceptor activity is colour coded: ■ shikimic acid and its derivatives, ■ quinic acid, ■ triterpenoids, ■ simple alcohols or amines with a carboxyl group, ■ flavonoid glycosides, ■ sugars, ■ other amines, ■ other alcohols, ■ other acceptors (e.g. alkaloids). Subclades as described by Kruse et al. (2022) (shown in brackets) and their orthogroup membership are summarized in new subclades

(a)

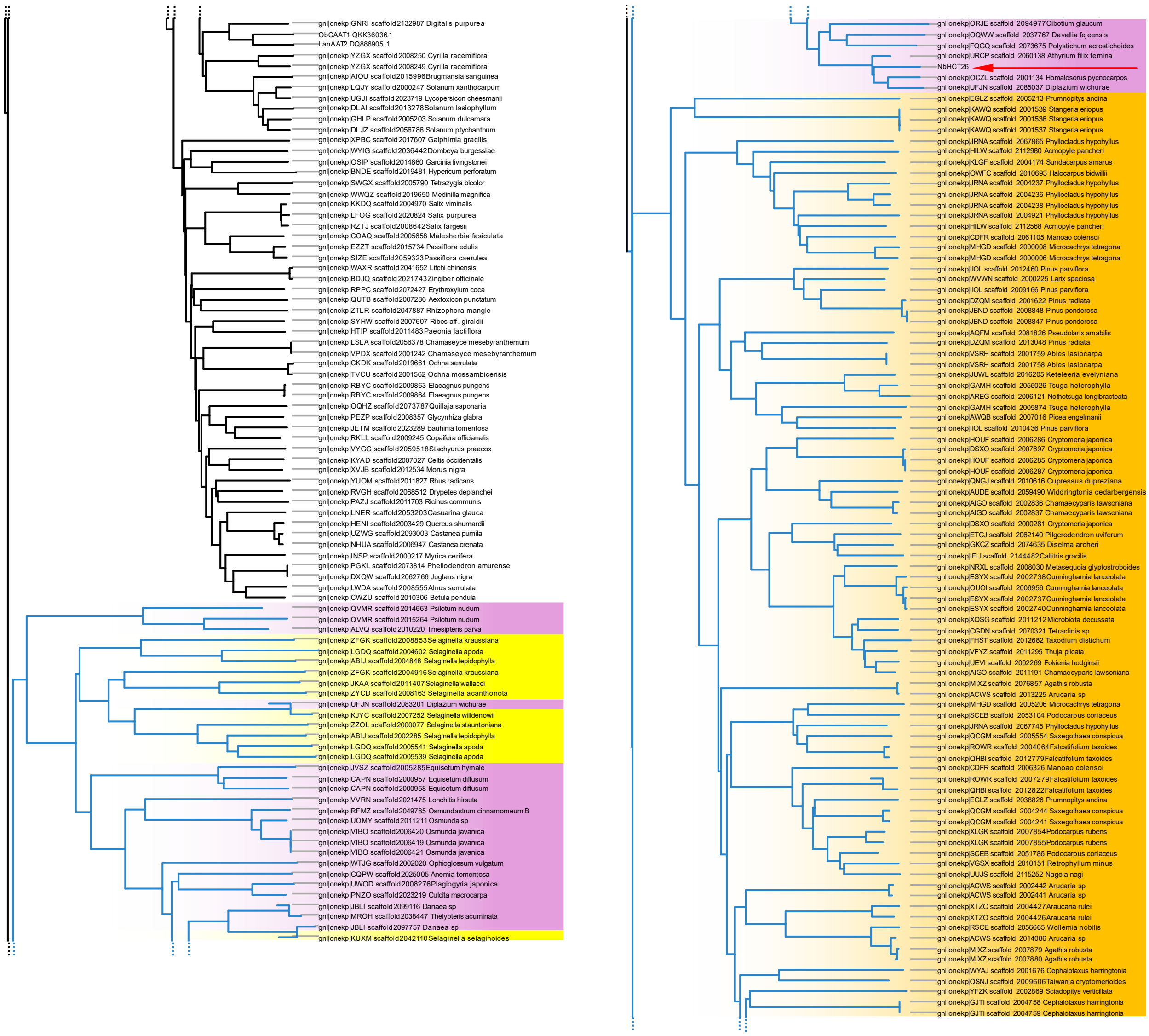
gnl|onekp|GNRI scaffold2132987 Digitalis purpurea
ObCAAT1 QKK36036.1
LanAAT2 DQ886905.1
gnl|onekp|YZGX scaffold2008250 Cyrilla racemiflora
gnl|onekp|YZGX scaffold2008249 Cyrilla racemiflora
gnl|onekp|AIOU scaffold2015996 Brugmansia sanguinea
gnl|onekp|LQJY scaffold2000247 Solanum xanthocarpum
gnl|onekp|UGJI scaffold2023719 Lycopersicon cheesmanii
gnl|onekp|DLAI scaffold2013278 Solanum lasiophyllum
gnl|onekp|GHLP scaffold2005203 Solanum dulcamara
gnl|onekp|DLJZ scaffold2056786 Solanum ptychanthum
gnl|onekp|XPBC scaffold2017607 Galphimia gracilis
gnl|onekp|WYIG scaffold2036442 Dombeya burgessiae
gnl|onekp|OSIP scaffold2014860 Garcinia livingstonei
gnl|onekp|BNDE scaffold2019481 Hypericum perforatum
gnl|onekp|SWGX scaffold2005790 Tetrazygia bicolor
gnl|onekp|WWQZ scaffold2019650 Medinilla magnifica
gnl|onekp|KKDQ scaffold2004970 Salix viminalis
gnl|onekp|LFOG scaffold2020824 Salix purpurea
gnl|onekp|RZTJ scaffold2008642 Salix fargesii
gnl|onekp|COAQ scaffold2005658 Malesherbia fasiculata
gnl|onekp|EZZT scaffold2015734 Passiflora edulis
gnl|onekp|SIZE scaffold2059323 Passiflora caerulea
gnl|onekp|WAXR scaffold2041652 Litchi chinensis
gnl|onekp|BDJQ scaffold2021743 Zingiber officinale
gnl|onekp|RPPC scaffold2072427 Erythroxylum coca
gnl|onekp|QUTB scaffold2007286 Aextoxicon punctatum
gnl|onekp|ZTLR scaffold2047887 Rhizophora mangle
gnl|onekp|SYHW scaffold2007607 Ribes aff. giraldii
gnl|onekp|HTIP scaffold2011483 Paeonia lactiflora
gnl|onekp|LSLA scaffold2056378 Chamaesyce mesebyranthemum
gnl|onekp|VPDX scaffold2001242 Chamaesyce mesebyranthemum
gnl|onekp|CKDK scaffold2019661 Ochna serrulata
gnl|onekp|TVCU scaffold2001562 Ochna mossambicensis
gnl|onekp|RBYC scaffold2009863 Elaeagnus pungens
gnl|onekp|RBYC scaffold2009864 Elaeagnus pungens
gnl|onekp|OQHZ scaffold2073787 Quillaja saponaria
gnl|onekp|PEZP scaffold2008357 Glycyrrhiza glabra
gnl|onekp|JETM scaffold2023289 Bauhinia tomentosa
gnl|onekp|RKLL scaffold2009245 Copaifera officinalis
gnl|onekp|VYGG scaffold2059518 Stachyurus praecox
gnl|onekp|KYAD scaffold2007027 Celtis occidentalis
gnl|onekp|XVJB scaffold2012534 Morus nigra
gnl|onekp|YUOM scaffold2011827 Rhus radicans
gnl|onekp|RVGH scaffold2068512 Drypetes deplanchei
gnl|onekp|PAZJ scaffold2011703 Ricinus communis
gnl|onekp|LNER scaffold2053203 Casuarina glauca
gnl|onekp|HENI scaffold2003429 Quercus shumardii
gnl|onekp|UZWG scaffold2093003 Castanea pumila
gnl|onekp|NHJA scaffold2006947 Castanea crenata
gnl|onekp|INSP scaffold2000217 Myrica cerifera
gnl|onekp|PGKL scaffold2073814 Phellodendron amurense
gnl|onekp|DXQW scaffold2062766 Juglans nigra
gnl|onekp|LWDA scaffold2008555 Alnus serrulata
gnl|onekp|CWZU scaffold2010306 Betula pendula
gnl|onekp|QVMR scaffold2014663 Psilotum nudum
gnl|onekp|QVMR scaffold2015264 Psilotum nudum
gnl|onekp|ALVQ scaffold2010220 Tmesipteris parva
gnl|onekp|ZFGK scaffold2008853 Selaginella kraussiana
gnl|onekp|LGDQ scaffold2004602 Selaginella apoda
gnl|onekp|ABIJ scaffold2004848 Selaginella lepidophylla
gnl|onekp|ZFGK scaffold2004916 Selaginella kraussiana
gnl|onekp|KAA scaffold2011407 Selaginella wallacei
gnl|onekp|ZYCD scaffold2008163 Selaginella acanthonota
gnl|onekp|UFJN scaffold2083201 Diplazium wichurae
gnl|onekp|KJYC scaffold2007252 Selaginella willdenowii
gnl|onekp|ZZOL scaffold2000077 Selaginella stauntoniana
gnl|onekp|ABIJ scaffold2002285 Selaginella lepidophylla
gnl|onekp|LGDQ scaffold2005541 Selaginella apoda
gnl|onekp|LGDQ scaffold2005539 Selaginella apoda
gnl|onekp|JVSZ scaffold2005285 Equisetum hymale
gnl|onekp|CAPN scaffold2000957 Equisetum diffusum
gnl|onekp|CAPN scaffold2000958 Equisetum diffusum
gnl|onekp|VVRN scaffold2021475 Lonchitis hirsuta
gnl|onekp|RFMZ scaffold2049785 Osmundastrum cinnamomeum B
gnl|onekp|UOMY scaffold2011211 Osmunda sp
gnl|onekp|VIBO scaffold2006420 Osmunda javanica
gnl|onekp|VIBO scaffold2006419 Osmunda javanica
gnl|onekp|VIBO scaffold2006421 Osmunda javanica
gnl|onekp|WTJG scaffold2002020 Ophioglossum vulgatum
gnl|onekp|CQPW scaffold2025005 Anemia tomentosa
gnl|onekp|UWOD scaffold2008276 Plagiogyria japonica
gnl|onekp|PNZO scaffold2023219 Culcita macrocarpa
gnl|onekp|JBLI scaffold2099116 Danaea sp
gnl|onekp|MROH scaffold2038447 Thelypteris acuminata
gnl|onekp|JBLI scaffold2097757 Danaea sp
gnl|onekp|KUXM scaffold2042110 Selaginella selaginoides
gnl|onekp|ORJE scaffold 2094977 Cibotium glaucum
gnl|onekp|OQWW scaffold 2037767 Davallia fejeensis
gnl|onekp|FQGQ scaffold 2073675 Polystichum acrostichoides
gnl|onekp|URCP scaffold 2060138 Athyrium filix femina
NbHCT26
gnl|onekp|OCZL scaffold 2001134 Homalosorus pycnocarpos
gnl|onekp|UFJN scaffold 2085037 Diplazium wichurae
gnl|onekp|EGLZ scaffold 2005213 Prumnopitys andina
gnl|onekp|KAWQ scaffold 2001539 Stangeria eriopus
gnl|onekp|KAWQ scaffold 2001536 Stangeria eriopus
gnl|onekp|KAWQ scaffold 2001537 Stangeria eriopus
gnl|onekp|JRNA scaffold 2067865 Phyllocladus hypophyllus
gnl|onekp|HILW scaffold 2112980 Acmopyle pancheri
gnl|onekp|KLGF scaffold 2004174 Sundacarpus amarus
gnl|onekp|OWFC scaffold 2010693 Halocarpus bidwillii
gnl|onekp|JRNA scaffold 2004237 Phyllocladus hypophyllus
gnl|onekp|JRNA scaffold 2004236 Phyllocladus hypophyllus
gnl|onekp|JRNA scaffold 2004238 Phyllocladus hypophyllus
gnl|onekp|JRNA scaffold 2004921 Phyllocladus hypophyllus
gnl|onekp|HILW scaffold 2112568 Acmopyle pancheri
gnl|onekp|CDFR scaffold 2061105 Manoao colensoi
gnl|onekp|MHGD scaffold 2000008 Microcachrys tetragona
gnl|onekp|MHGD scaffold 2000006 Microcachrys tetragona
gnl|onekp|IIOL scaffold 2012460 Pinus parviflora
gnl|onekp|WVWN scaffold 2000225 Larix speciosa
gnl|onekp|IIOL scaffold 2009166 Pinus parviflora
gnl|onekp|DZQM scaffold 2001622 Pinus radiata
gnl|onekp|JBND scaffold 2008848 Pinus ponderosa
gnl|onekp|JBND scaffold 2008847 Pinus ponderosa
gnl|onekp|AQFM scaffold 2081826 Pseudolarix amabilis
gnl|onekp|DZQM scaffold 2013048 Pinus radiata
gnl|onekp|VSRH scaffold 2001759 Abies lasiocarpa
gnl|onekp|VSRH scaffold 2001758 Abies lasiocarpa
gnl|onekp|JUWL scaffold 2016205 Keteleeria evelyniana
gnl|onekp|GAMH scaffold 2055026 Tsuga heterophylla
gnl|onekp|AREG scaffold 2006121 Nothotsuga longibracteata
gnl|onekp|GAMH scaffold 2005874 Tsuga heterophylla
gnl|onekp|AWQB scaffold 2007016 Picea engelmanii
gnl|onekp|IIOL scaffold 2010436 Pinus parviflora
gnl|onekp|HOUF scaffold 2006286 Cryptomeria japonica
gnl|onekp|DSXO scaffold 2007697 Cryptomeria japonica
gnl|onekp|HOUF scaffold 2006285 Cryptomeria japonica
gnl|onekp|HOUF scaffold 2006287 Cryptomeria japonica
gnl|onekp|QNGJ scaffold 2010616 Cupressus dupreziana
gnl|onekp|AUDE scaffold 2059490 Widdringtonia cedarbergensis
gnl|onekp|AIGO scaffold 2002836 Chamaecyparis lawsoniana
gnl|onekp|AIGO scaffold 2002837 Chamaecyparis lawsoniana
gnl|onekp|DSXO scaffold 2000281 Cryptomeria japonica
gnl|onekp|ETCJ scaffold 2062140 Pilgerodendron uviferum
gnl|onekp|GKCZ scaffold 2074635 Diselma archeri
gnl|onekp|IFLI scaffold 2144482 Callitris gracilis
gnl|onekp|NRXL scaffold 2008030 Metasequoia glyptostroboides
gnl|onekp|ESYX scaffold 2002738 Cunninghamia lanceolata
gnl|onekp|OUOI scaffold 2006956 Cunninghamia lanceolata
gnl|onekp|ESYX scaffold 2002737 Cunninghamia lanceolata
gnl|onekp|ESYX scaffold 2002740 Cunninghamia lanceolata
gnl|onekp|XQSG scaffold 2011212 Microbiota decussata
gnl|onekp|CGDN scaffold 2070321 Tetraclinis sp
gnl|onekp|FHST scaffold 2012682 Taxodium distichum
gnl|onekp|VFYZ scaffold 2011295 Thuja plicata
gnl|onekp|UEVI scaffold 2002269 Fokienia hodginsii
gnl|onekp|AIGO scaffold 2011191 Chamaecyparis lawsoniana
gnl|onekp|MIXZ scaffold 2076857 Agathis robusta
gnl|onekp|ACWS scaffold 2013225 Arucaria sp
gnl|onekp|MHGD scaffold 2005206 Microcachrys tetragona
gnl|onekp|SCEB scaffold 2053104 Podocarpus coriaceus
gnl|onekp|JRNA scaffold 2067745 Phyllocladus hypophyllus
gnl|onekp|QCGM scaffold 2005554 Saxegothaea conspicua
gnl|onekp|ROWR scaffold 2004064 Falcatifolium taxoides
gnl|onekp|QHBI scaffold 2012779 Falcatifolium taxoides
gnl|onekp|CDFR scaffold 2006326 Manoao colensoi
gnl|onekp|ROWR scaffold 2007279 Falcatifolium taxoides
gnl|onekp|QHBI scaffold 2012822 Falcatifolium taxoides
gnl|onekp|EGLZ scaffold 2038826 Prumnopitys andina
gnl|onekp|QCGM scaffold 2004244 Saxegothaea conspicua
gnl|onekp|QCGM scaffold 2004241 Saxegothaea conspicua
gnl|onekp|XLGK scaffold 2007854 Podocarpus rubens
gnl|onekp|XLGK scaffold 2007855 Podocarpus rubens
gnl|onekp|SCEB scaffold 2051786 Podocarpus coriaceus
gnl|onekp|VGSX scaffold 2010151 Retrophyllum minus
gnl|onekp|UUJS scaffold 2115252 Nageia nagi
gnl|onekp|ACWS scaffold 2002442 Arucaria sp
gnl|onekp|ACWS scaffold 2002441 Arucaria sp
gnl|onekp|XTZO scaffold 2004427 Araucaria rulei
gnl|onekp|XTZO scaffold 2004426 Araucaria rulei
gnl|onekp|RSCE scaffold 2056665 Wollemia nobilis
gnl|onekp|ACWS scaffold 2014086 Arucaria sp
gnl|onekp|MIXZ scaffold 2007879 Agathis robusta
gnl|onekp|MIXZ scaffold 2007880 Agathis robusta
gnl|onekp|WYAJ scaffold 2001676 Cephalotaxus harringtonia
gnl|onekp|QSNJ scaffold 2009606 Taiwania cryptomerioides
gnl|onekp|YFZK scaffold 2002869 Sciadopitys verticillata
gnl|onekp|GJTI scaffold 2004758 Cephalotaxus harringtonia
gnl|onekp|GJTI scaffold 2004759 Cephalotaxus harringtonia

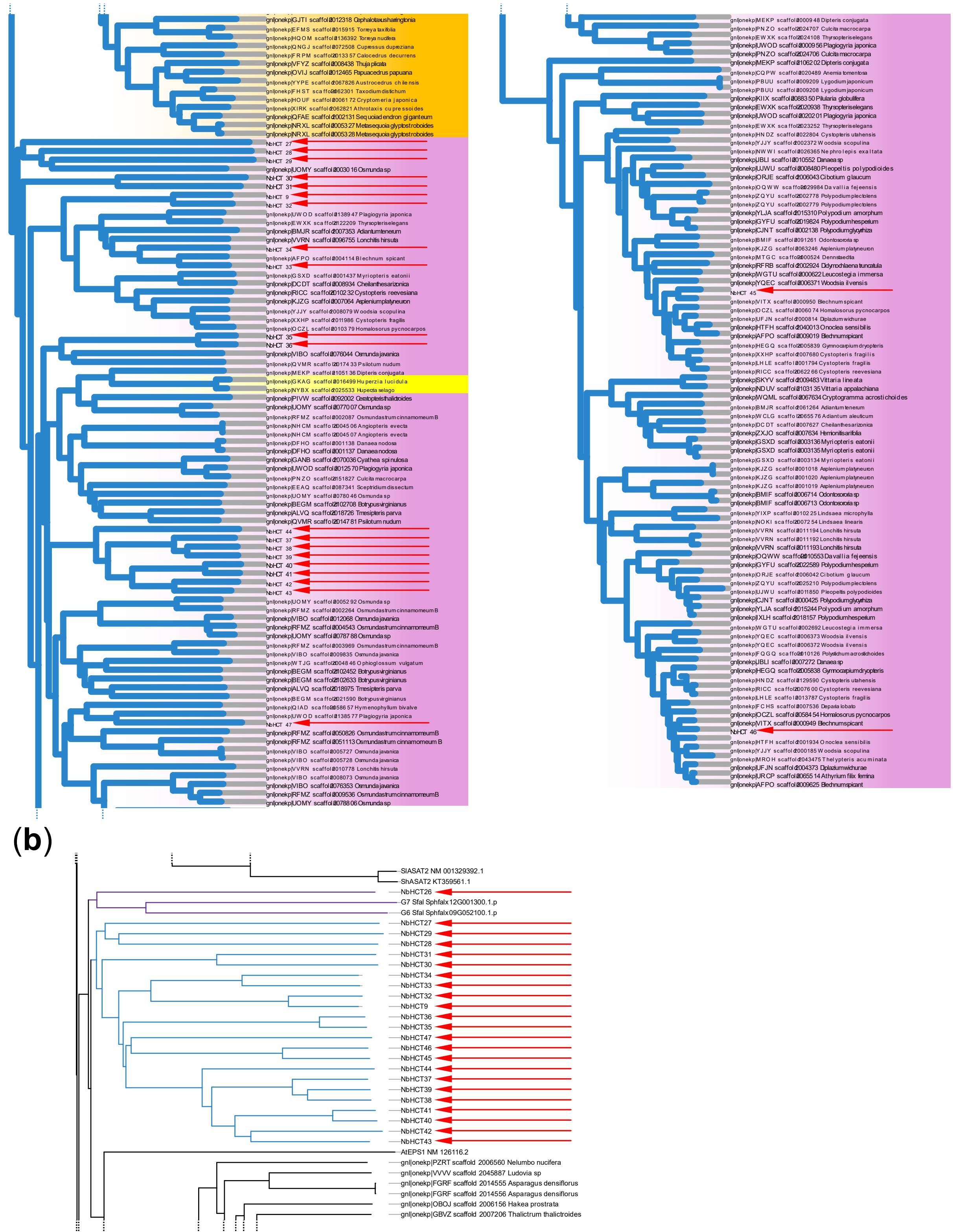

Fig. S15 Orthogroup analysis of NbHCT9 and NbHCT26-47. Excerpt of the final phylogenetic trees (indicated by the dotted lines). Blue branches indicate a monophyletic orthogroup. *Neoblechnum brasiliense* sequences are indicated by a red arrow. (**a**) Phylogenetic tree generated with all blastp results that are sorted into the orthogroup. Color code: ■ Angiosperms, ■ Gymnosperms, ■ Monilophytes (■ Member of the order Cyatheales, ■ Member of the order Polypodiales), ■ Lycophytes, ■ Bryophytes *sensu stricto*,

■ Anthocerotophytes, ■ Marchantiophytes. (**b**) Phylogenetic tree generated excluding gymnosperms, monilophytes and lycophytes in the blastp searches. Purple branch: Second monophyletic group in the negative control

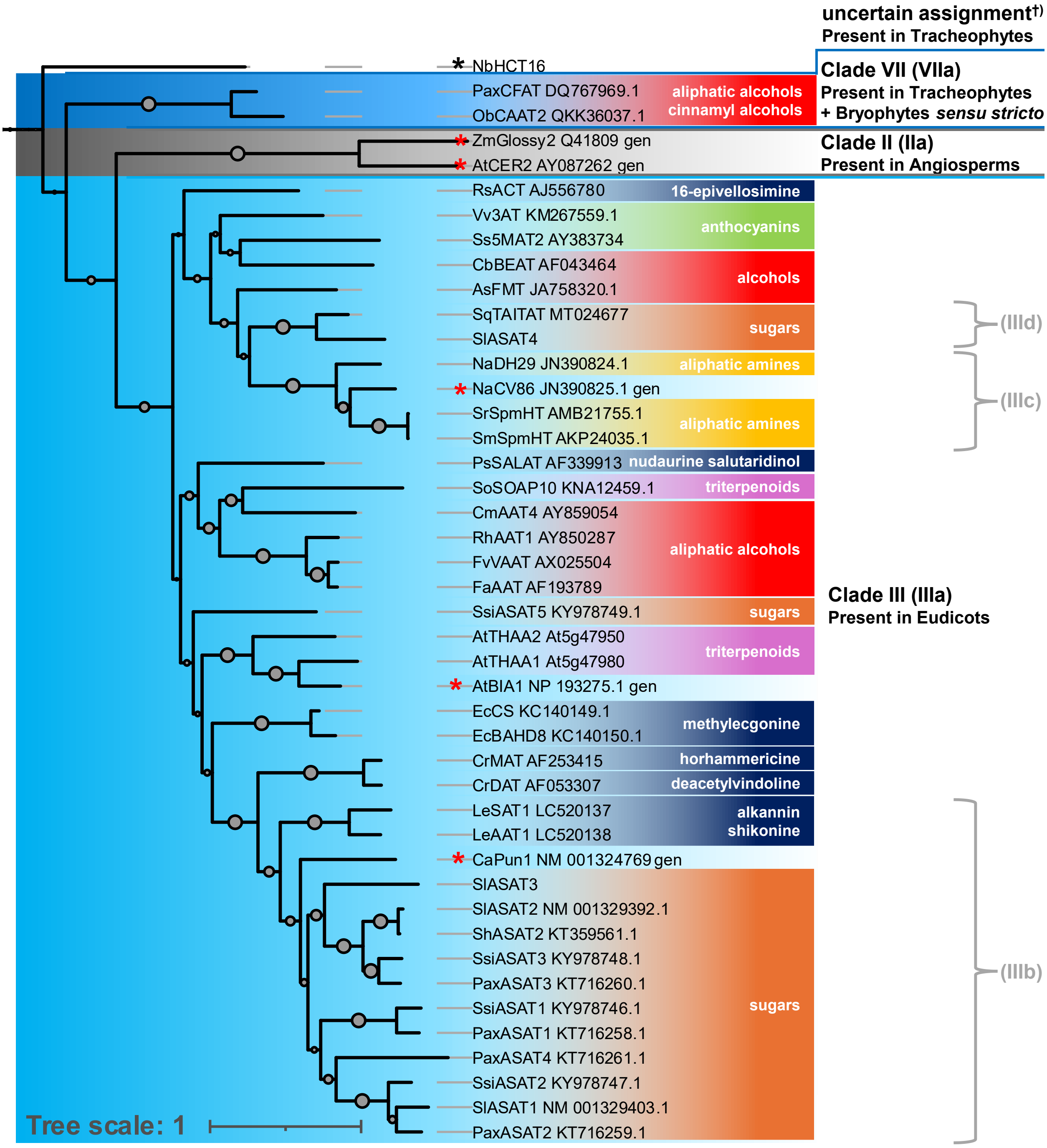

Fig. S16 Phylogenetic tree of clade II, III, VII and NbHCT16 of the tree shown in Fig. 3. The bootstrap support is shown by scaled grey circles. Sequences from *N. brasiliense* are marked with a black asterisk. Non-*N. brasiliense* sequences without known substrates are marked with a red asterisk. Acyl acceptor activity is colour coded: ■ shikimic acid and its derivatives, ■ quinic acid, ■ triterpenoids, ■ simple alcohols or amines with a carboxyl group, ■ flavonoid glycosides, ■ sugars, ■ other amines, ■ other alcohols, ■ other acceptors (e.g. alkaloids).

Subclades as described by Kruse et al. (2022) (shown in brackets) and their orthogroup membership are summarized in new subclades

(a)

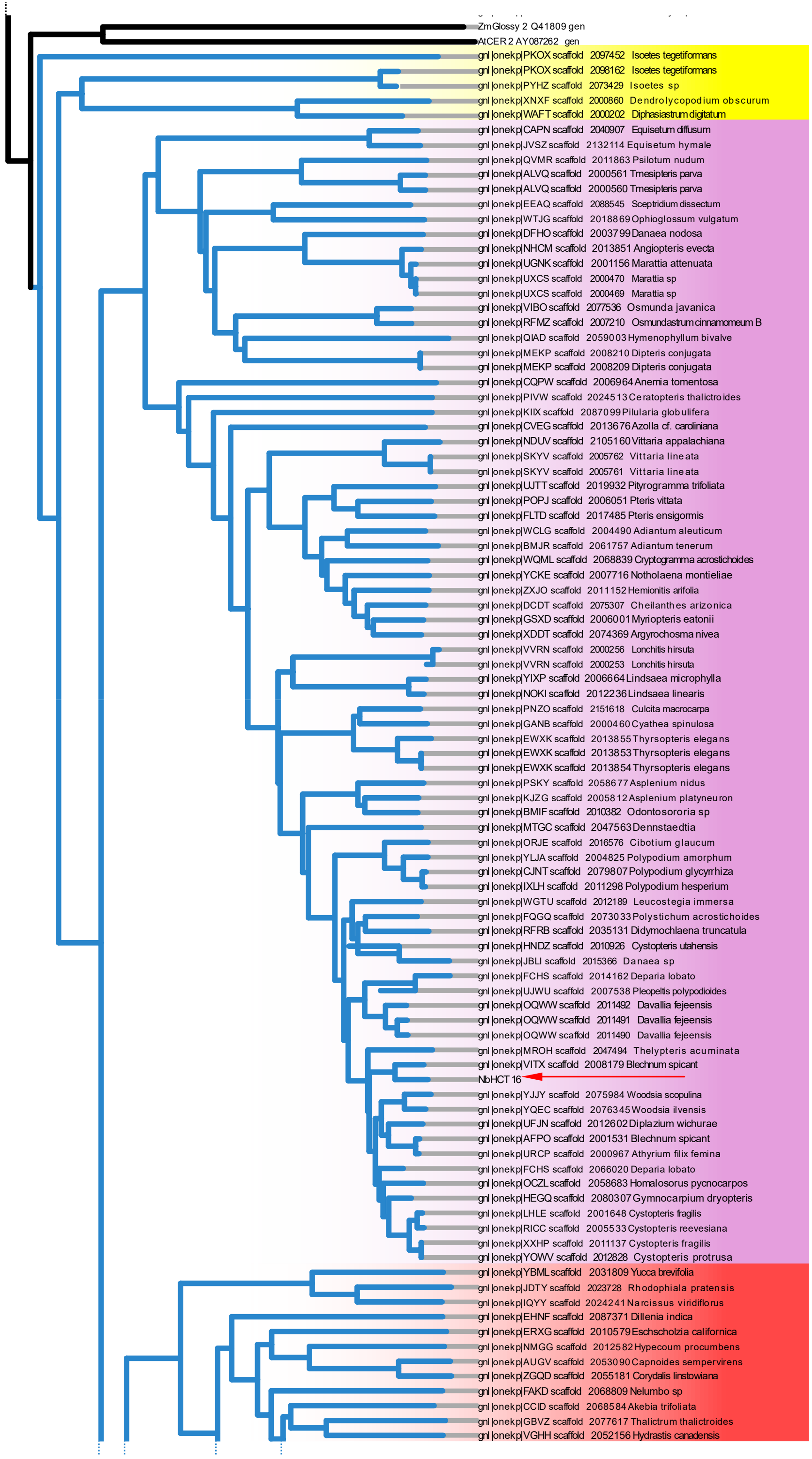

ZmGlossy 2 Q41809 gen
AtCER 2 AY087262 gen
gnl |onekp|PKOX scaffold 2097452 Isoetes tegetiformans
gnl |onekp|PKOX scaffold 2098162 Isoetes tegetiformans
gnl |onekp|PYHZ scaffold 2073429 Isoetes sp
gnl |onekp|XNXF scaffold 2000860 Dendrolycopodium obscurum
gnl |onekp|WAFT scaffold 2000202 Diphasiastrum digitatum
gnl |onekp|CAPN scaffold 2040907 Equisetum diffusum
gnl |onekp|JVSZ scaffold 2132114 Equisetum hymale
gnl |onekp|QVMR scaffold 2011863 Psilotum nudum
gnl |onekp|ALVQ scaffold 2000561 Tmesipteris parva
gnl |onekp|ALVQ scaffold 2000560 Tmesipteris parva
gnl |onekp|EEAQ scaffold 2088545 Sceptridium dissectum
gnl |onekp|WTJG scaffold 2018869 Ophioglossum vulgatum
gnl |onekp|DFHO scaffold 2003799 Danaea nodosa
gnl |onekp|NHCM scaffold 2013851 Angiopteris evecta
gnl |onekp|UGNK scaffold 2001156 Marattia attenuata
gnl |onekp|UXCS scaffold 2000470 Marattia sp
gnl |onekp|UXCS scaffold 2000469 Marattia sp
gnl |onekp|VIBO scaffold 2077536 Osmunda javanica
gnl |onekp|RFMZ scaffold 2007210 Osmundastrum cinnamomeum B
gnl |onekp|QIAD scaffold 2059003 Hymenophyllum bivalve
gnl |onekp|MEKP scaffold 2008210 Dipteris conjugata
gnl |onekp|MEKP scaffold 2008209 Dipteris conjugata
gnl |onekp|CQPW scaffold 2006964 Anemia tomentosa
gnl |onekp|PIVW scaffold 2024513 Ceratopteris thalictroides
gnl |onekp|KIIX scaffold 2087099 Pilularia globulifera
gnl |onekp|CVEG scaffold 2013676 Azolla cf. caroliniana
gnl |onekp|NDUV scaffold 2105160 Vittaria appalachiana
gnl |onekp|SKYV scaffold 2005762 Vittaria lineata
gnl |onekp|SKYV scaffold 2005761 Vittaria lineata
gnl |onekp|UJTT scaffold 2019932 Pityrogramma trifoliata
gnl |onekp|POPJ scaffold 2006051 Pteris vittata
gnl |onekp|FLTD scaffold 2017485 Pteris ensigormis
gnl |onekp|WCLG scaffold 2004490 Adiantum aleuticum
gnl |onekp|BMJR scaffold 2061757 Adiantum tenerum
gnl |onekp|WQML scaffold 2068839 Cryptogramma acrostichoides
gnl |onekp|YCKE scaffold 2007716 Notholaena montieliae
gnl |onekp|ZXJO scaffold 2011152 Hemionitis arifolia
gnl |onekp|DCDT scaffold 2075307 Cheilanthes arizonica
gnl |onekp|GSXD scaffold 2006001 Myriopteris eatonii
gnl |onekp|XDDT scaffold 2074369 Argyrochosma nivea
gnl |onekp|VVRN scaffold 2000256 Lonchitis hirsuta
gnl |onekp|VVRN scaffold 2000253 Lonchitis hirsuta
gnl |onekp|YIXP scaffold 2006664 Lindsaea microphylla
gnl |onekp|NOKI scaffold 2012236 Lindsaea linearis
gnl |onekp|PNZO scaffold 2151618 Culcita macrocarpa
gnl |onekp|GANB scaffold 2000460 Cyathea spinulosa
gnl |onekp|EWXK scaffold 2013855 Thyrsopteris elegans
gnl |onekp|EWXK scaffold 2013853 Thyrsopteris elegans
gnl |onekp|EWXK scaffold 2013854 Thyrsopteris elegans
gnl |onekp|PSKY scaffold 2058677 Asplenium nidus
gnl |onekp|KJZG scaffold 2005812 Asplenium platyneuron
gnl |onekp|BMIF scaffold 2010382 Odontosororia sp
gnl |onekp|MTGC scaffold 2047563 Dennstaedtia
gnl |onekp|ORJE scaffold 2016576 Cibotium glaucum
gnl |onekp|YLJA scaffold 2004825 Polypodium amorphum
gnl |onekp|CJNT scaffold 2079807 Polypodium glycyrrhiza
gnl |onekp|IXLH scaffold 2011298 Polypodium hesperium
gnl |onekp|WGTU scaffold 2012189 Leucostegia immersa
gnl |onekp|FQGQ scaffold 2073033 Polystichum acrostichoides
gnl |onekp|RFRB scaffold 2035131 Didymochlaena truncatula
gnl |onekp|HNDZ scaffold 2010926 Cystopteris utahensis
gnl |onekp|JBLI scaffold 2015366 Danaea sp
gnl |onekp|FCHS scaffold 2014162 Deparia lobato
gnl |onekp|UJWU scaffold 2007538 Pleopeltis polypodioides
gnl |onekp|OQWW scaffold 2011492 Davallia fejeensis
gnl |onekp|OQWW scaffold 2011491 Davallia fejeensis
gnl |onekp|OQWW scaffold 2011490 Davallia fejeensis
gnl |onekp|MROH scaffold 2047494 Thelypteris acuminata
gnl |onekp|VITX scaffold 2008179 Blechnum spicant
NbHCT 16
gnl |onekp|YJJY scaffold 2075984 Woodsia scopulina
gnl |onekp|YQEC scaffold 2076345 Woodsia ilvensis
gnl |onekp|UFJN scaffold 2012602 Diplazium wichurae
gnl |onekp|AFPO scaffold 2001531 Blechnum spicant
gnl |onekp|URCP scaffold 2000967 Athyrium filix femina
gnl |onekp|FCHS scaffold 2066020 Deparia lobato
gnl |onekp|OCZL scaffold 2058683 Homalosorus pycnocarpos
gnl |onekp|HEGQ scaffold 2080307 Gymnocarpium dryopteris
gnl |onekp|LHLE scaffold 2001648 Cystopteris fragilis
gnl |onekp|RICC scaffold 2005533 Cystopteris reevesiana
gnl |onekp|XXHP scaffold 2011137 Cystopteris fragilis
gnl |onekp|YOWV scaffold 2012828 Cystopteris protrusa
gnl |onekp|YBML scaffold 2031809 Yucca brevifolia
gnl |onekp|JDTY scaffold 2023728 Rhodophiala pratensis
gnl |onekp|IQYY scaffold 2024241 Narcissus viridiflorus
gnl |onekp|EHNF scaffold 2087371 Dillenia indica
gnl |onekp|ERXG scaffold 2010579 Eschscholzia californica
gnl |onekp|NMGG scaffold 2012582 Hypecoum procumbens
gnl |onekp|AUGV scaffold 2053090 Capnoides sempervirens
gnl |onekp|ZGQD scaffold 2055181 Corydalis linstowiana
gnl |onekp|FAKD scaffold 2068809 Nelumbo sp
gnl |onekp|CCID scaffold 2068584 Akebia trifoliata
gnl |onekp|GBVZ scaffold 2077617 Thalictrum thalictroides
gnl |onekp|VGHH scaffold 2052156 Hydrastis canadensis

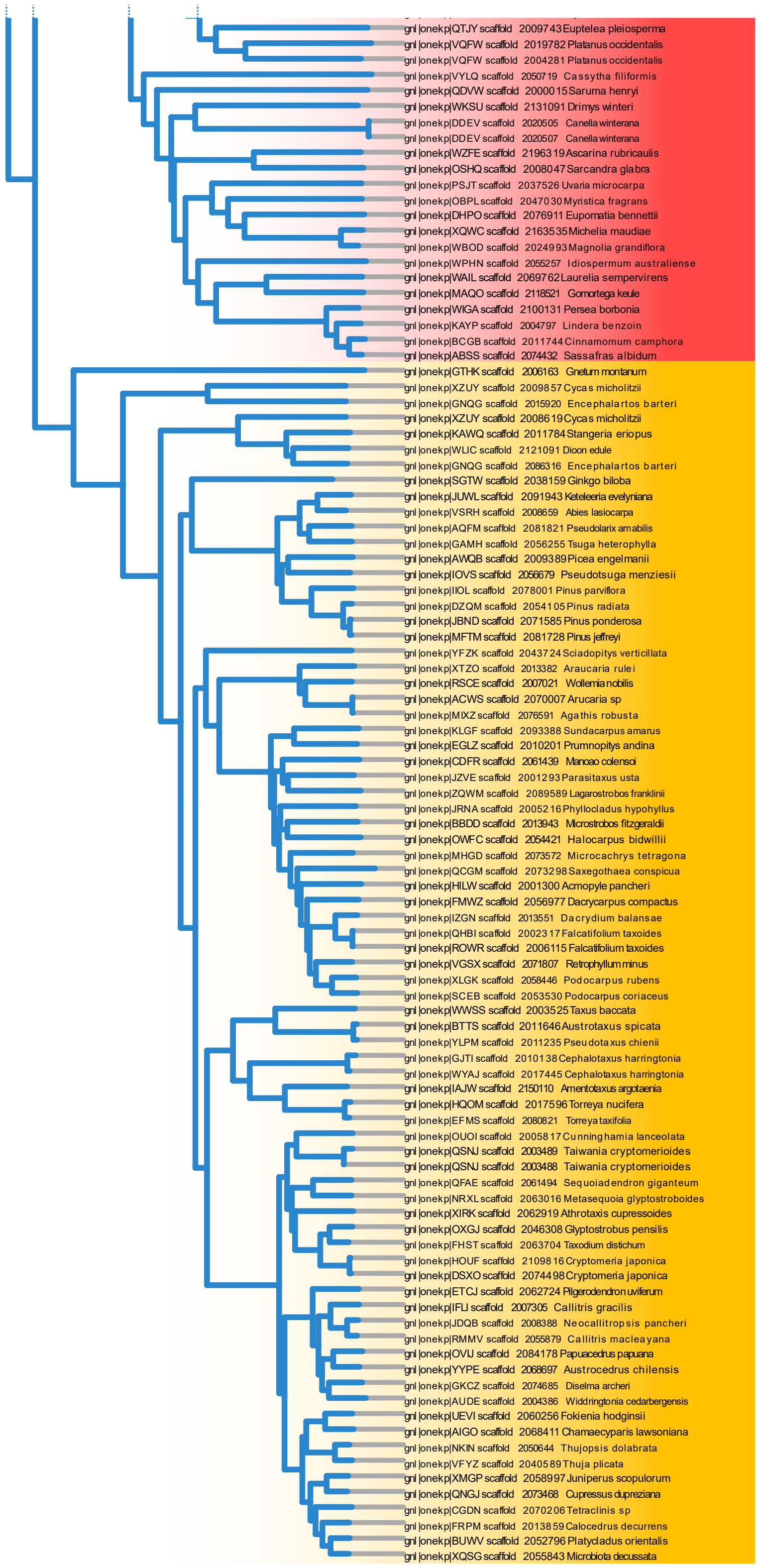

(**b**)

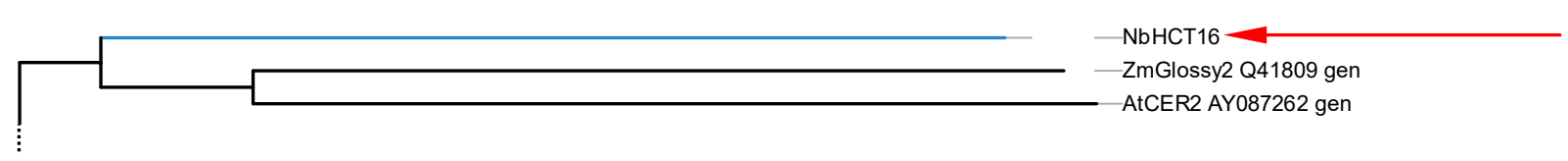

Fig. S17 Orthogroup analysis of NbHCT16. Excerpt of the final phylogenetic trees (indicated by the dotted lines). Blue branches indicate a monophyletic orthogroup. *Neoblechnum brasiliense* sequences are indicated by a red arrow. (**a**) Phylogenetic tree generated with all

blastp results that are sorted into the orthogroup. Color code: ■ Angiosperms, ■ Gymnosperms, ■ Monilophytes (■ Member of the order Cyatheales, ■ Member of the order Polypodiales), ■ Lycophytes, ■ Bryophytes *sensu stricto*, ■ Anthocerotophytes, ■ Marchantiophytes. (**b**) Phylogenetic tree generated excluding tracheophytes in the blastp searches

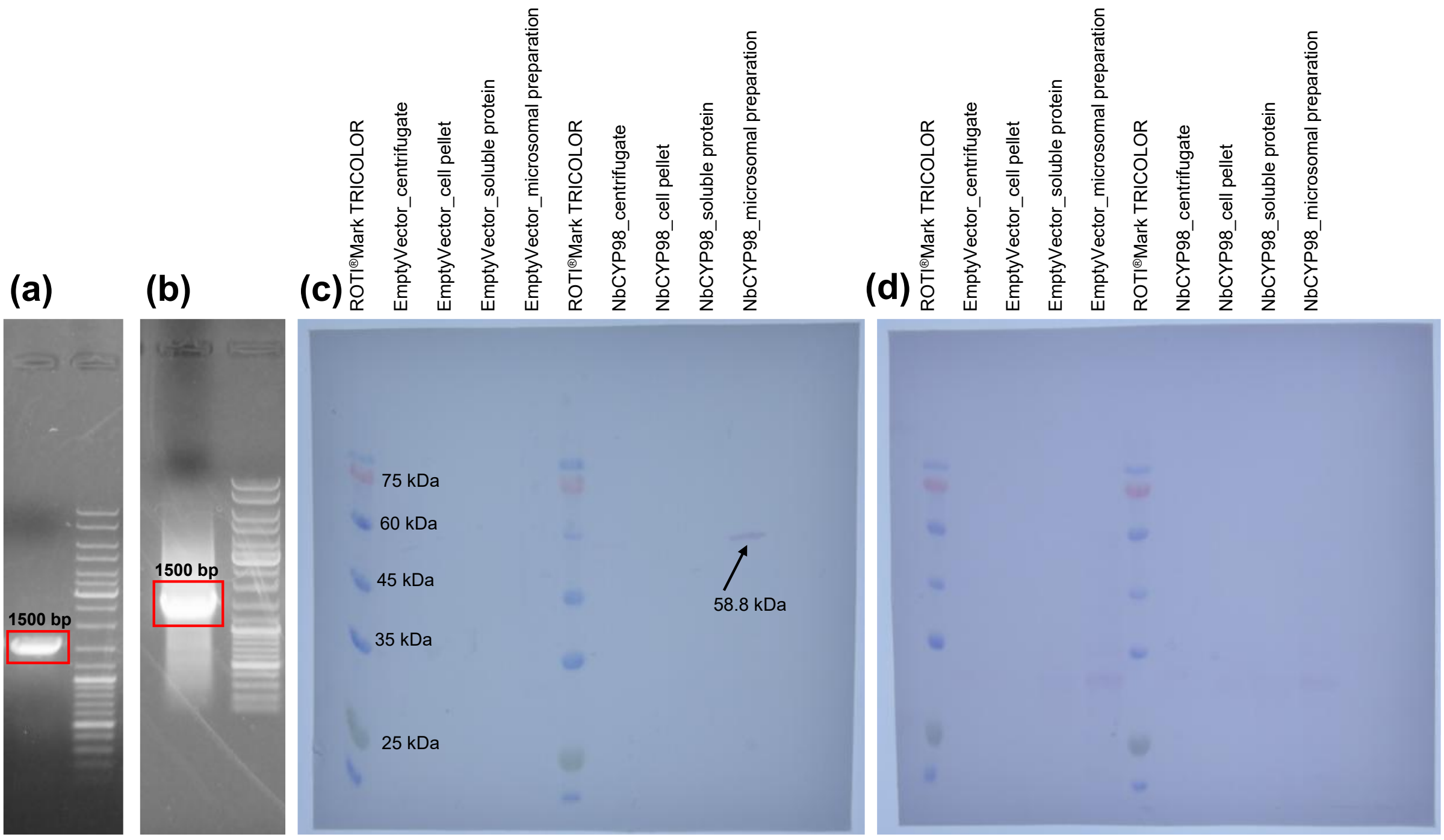

Fig. S18 PCR reactions and Western blots of NbCYP98. **(a)** Full-length PCR of NbCYP98 with restriction sites, **(b)** full-length PCR of the codon-optimized NbCYP98 with restriction sites and overhang with the pESC-URA vector for homologous recombination, **(c)** Western blot of the empty vector control and the codon-optimized NbCYP98 + CbCPR with a primary antibody against the FLAG-tag (attached to NbCYP98), the arrow points to NbCYP98, 58.8 kDa is the calculated value by ProtParam, **(d)** Western blot of the empty vector control and NbCYP98 with a primary antibody against the myc-tag (attached to CbCPR)

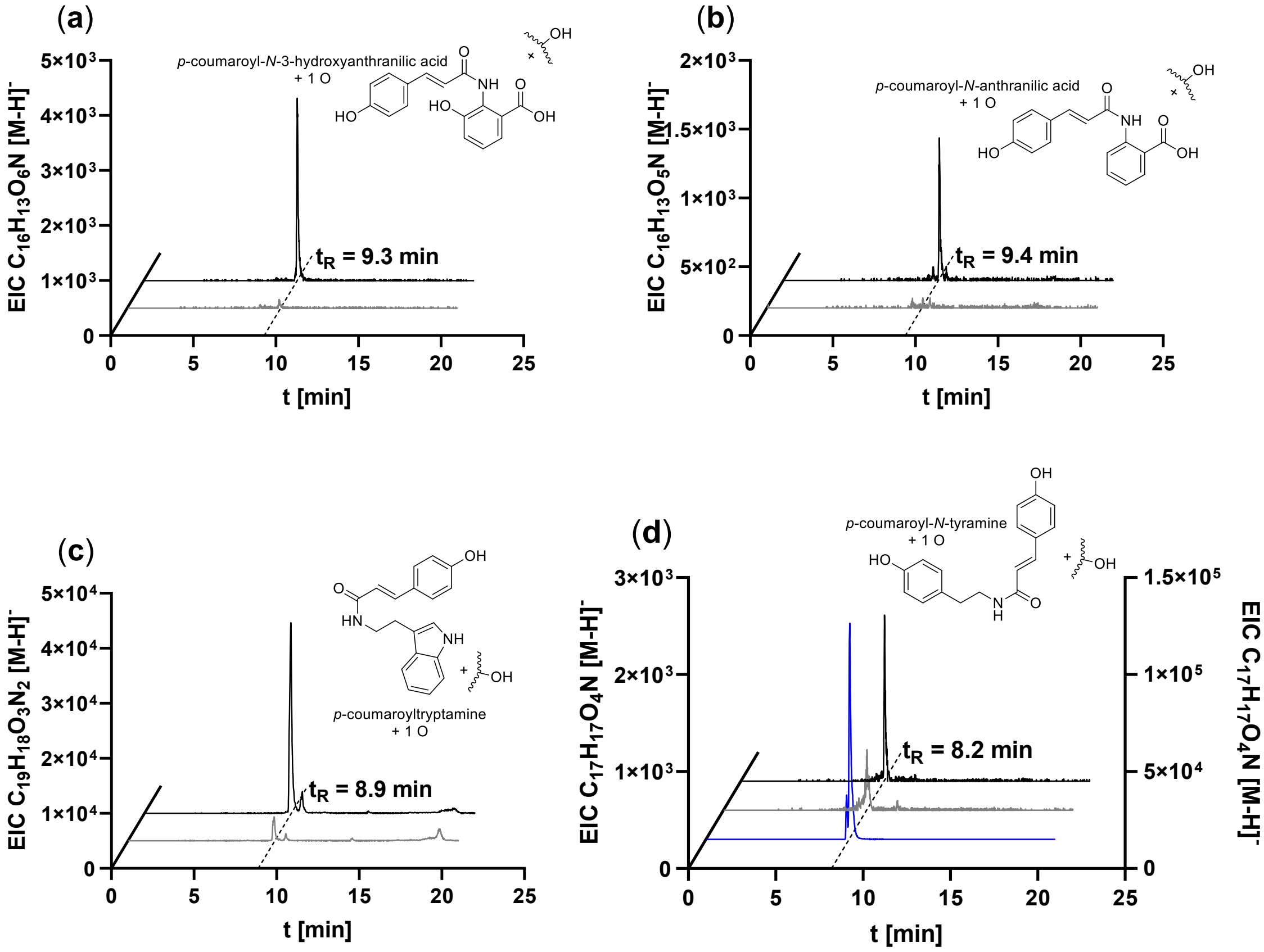

Fig. S19 Extracted ion chromatograms of *p*-coumaroyl amides transformed by NbCYP98A268. Blue line: reference substance, grey line: empty vector control, black line: incubation with NbHCT98A268. **(a)** Hydroxylation of *p*-coumaroyl-*N*-3-hydroxyanthranilic acid, **(b)** hydroxylation of *p*-coumaroyl-*N*-anthranilic acid, **(c)** hydroxylation of *p*-coumaroyltryptamine, **(d)** hydroxylation of *p*-coumaroyl-*N*-tyramine. Values plotted on the left ordinate except reference which is plotted on the right ordinate

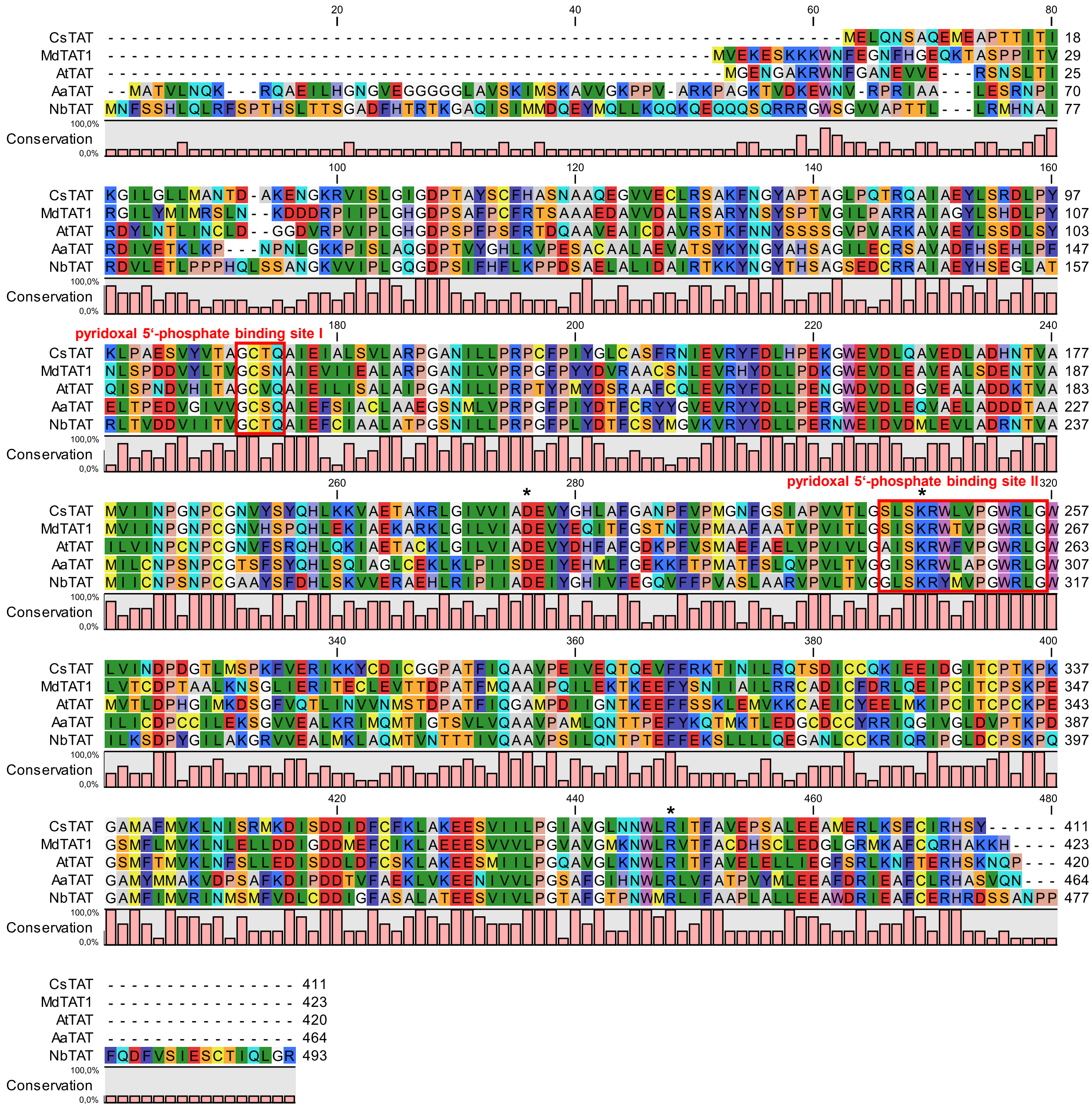

Fig. S20 Alignment of a putative sequence for tyrosine aminotransferase (TAT) from *Neoblechnum brasiliense* (NbTAT PZ166773) with other plant TAT sequences (*Coleus scutellarioides* (Q8GUE9), *Malus domestica* TAT1 (KY359351), *Arabidopsis thaliana* (Q9LVY1), *Anthoceros agrestis* (MN922307)). Conserved pyridoxal 5'-phosphate binding sites I and II are marked with red boxes (Busch and Petersen 2021). Amino acids important for catalytic activity are marked with a black asterisk. Alignment created with Clustal Omega (www.theclustal.com) and CLC sequence viewer 8.0.0

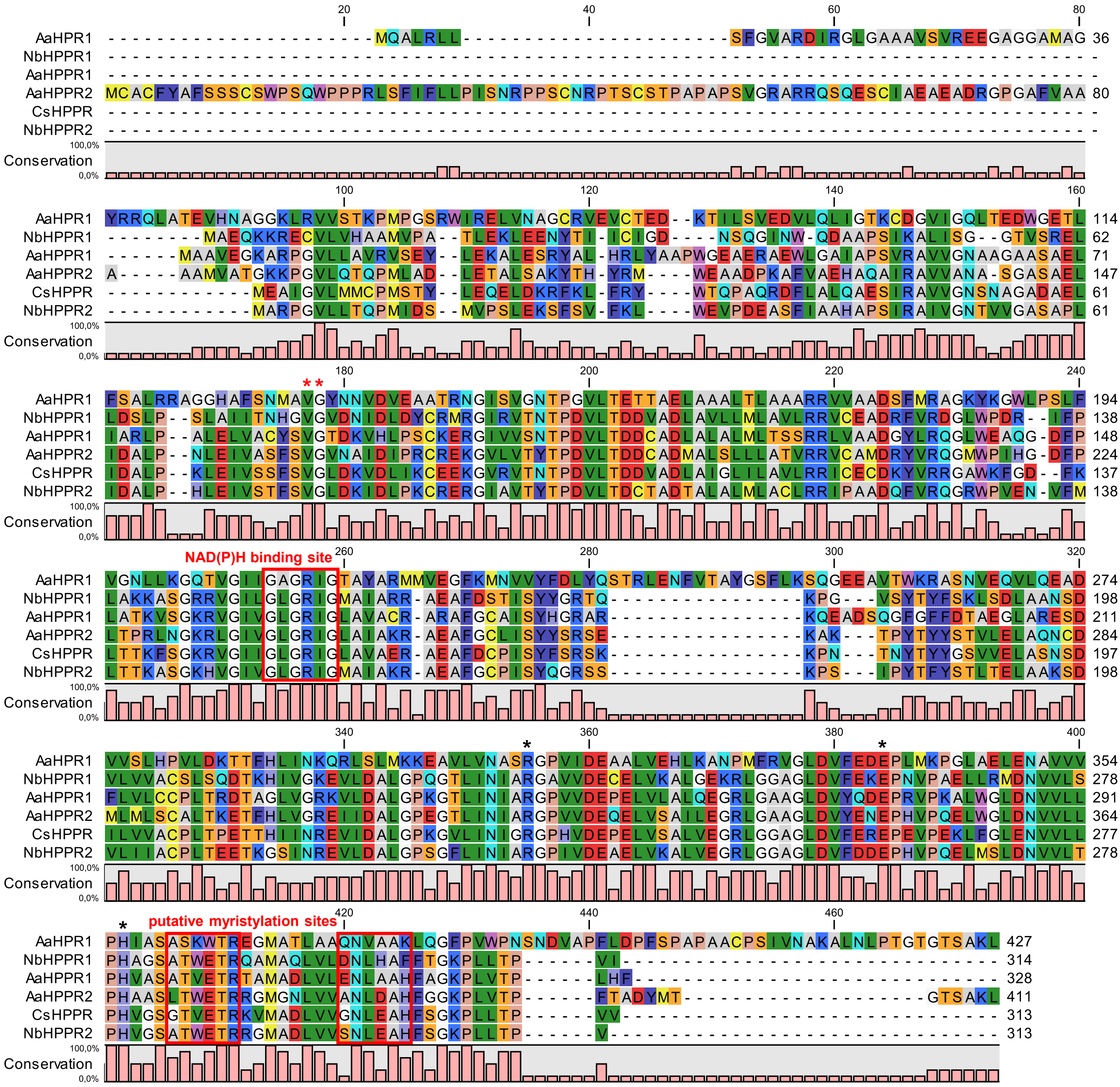

Fig. S21 Alignment of putative hydroxyphenylpyruvate reductase (HPPR) sequences from *Neoblechnum brasiliense* (NbHPPR1, PZ166774; NbHPPR2, PZ166775) with other plant HPPR sequences (*Anthoceros agrestis*, Busch 2022; *Coleus scutellarioides* (Q65CJ7)) and hydroxypyruvate reductase (HPR) from *Anthoceros agrestis*; Busch 2022). Catalytically important amino acids are marked with a black asterisk, amino acids involved in substrate binding are marked with red asterisks (Janiak et al. 2010). Catalytic sites are marked with red boxes (Wang et al. 2017). Alignment created with Clustal Omega (www.theclustal.com) and CLC sequence viewer 8.0.0

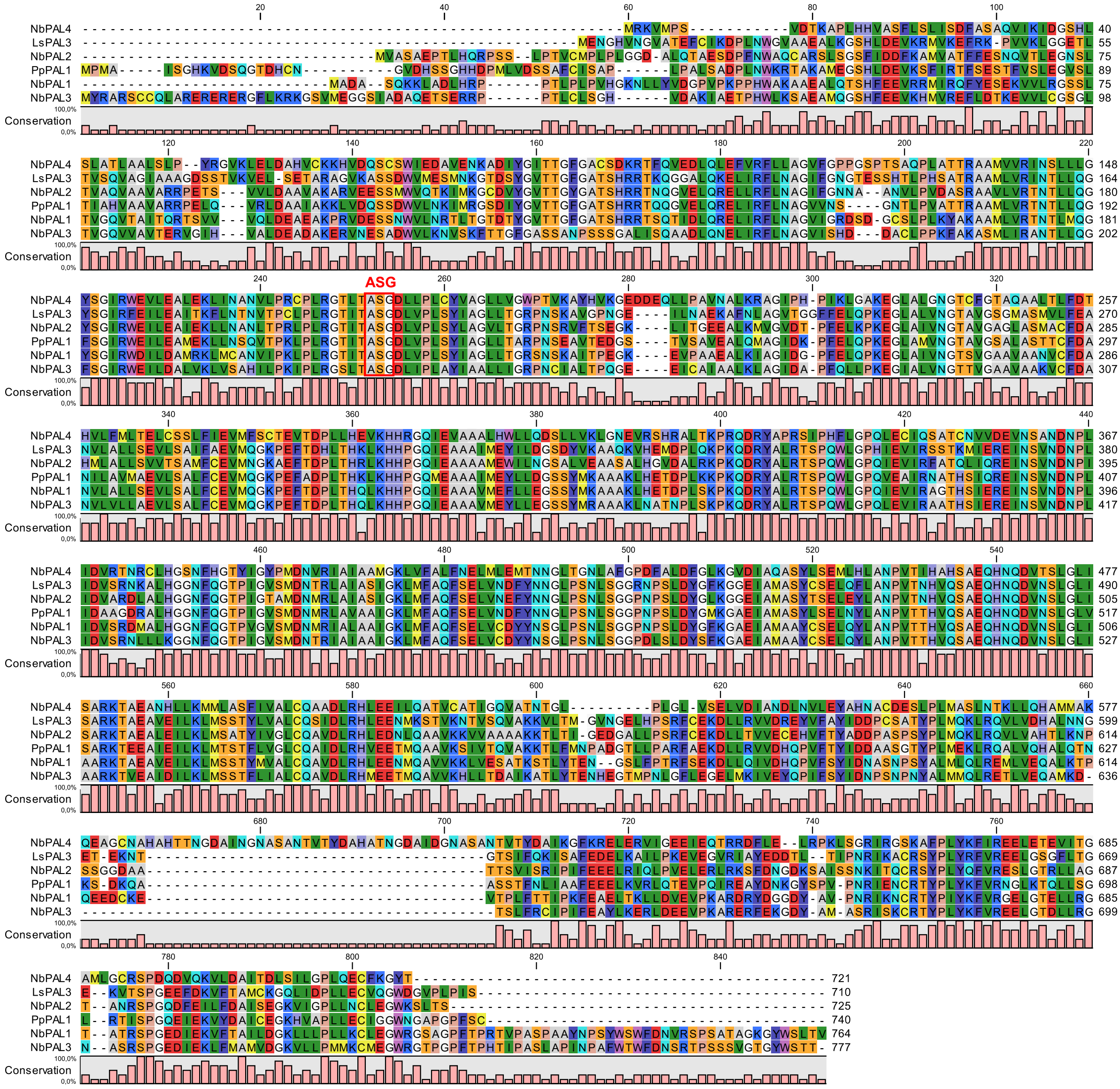

Fig. S22 Alignment of putative phenylalanine ammonia-lyase (PAL) sequences from *Neoblechnum brasiliense* (NbPAL1, PZ166758; NbPAL2, PZ166759; NbPAL3, PZ166760; NbPAL4, PZ166761) with other plant PAL sequences (*Physcomitrium patens* PAL1 (A0A2K1L8S9), *Lactuca sativa* PAL3 (XP_023767814.1)). The ASG motif (red box) forms a 3,5-dihydro-5-methylidene-4H-imidazol-4-one (MIO) group which is the reactive centre of PAL (Ritter and Schulz 2004). Alignment created with Clustal Omega (www.theclustal.com) and CLC sequence viewer 8.0.0

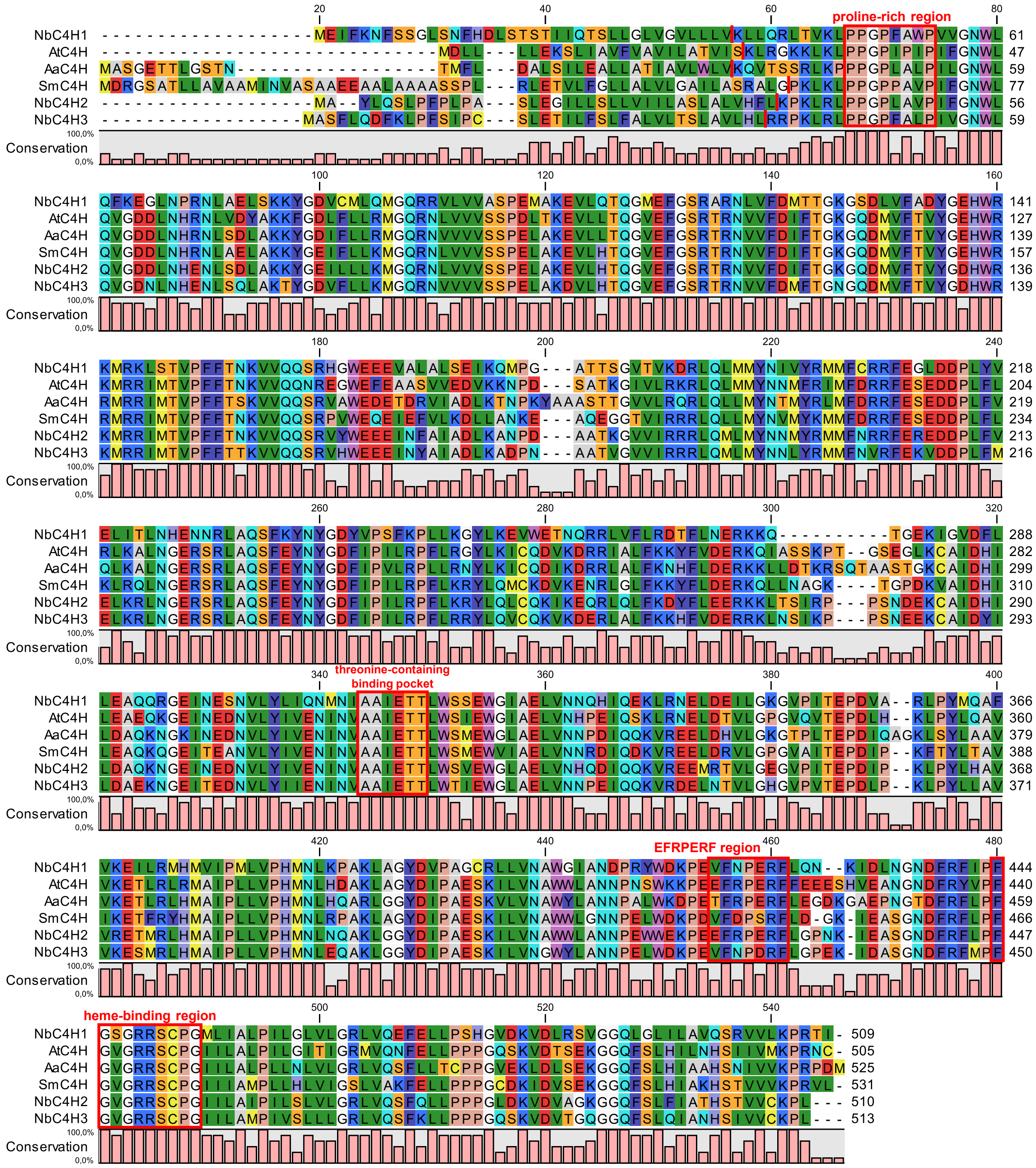

Fig. S23 Alignment of putative cinnamate 4-hydroxylase (C4H) sequences from *Neoblechnum brasiliense* (NbC4H1, PZ166762; NbC4H2, PZ166763; NbC4H3, PZ166764) with other plant C4H sequences (*Selaginella moellendorffii* (EFJ22128), *Anthoceros agrestis* (MK778366), *Arabidopsis thaliana* (CAP08840)). Conserved motifs are marked with red boxes. Trans-membrane domains were predicted with TMHMM 2.0 (Krogh et al. 2001) and are indicated by red vertical lines. Alignment created with Clustal Omega (www.theclustal.com) and CLC sequence viewer 8.0.0

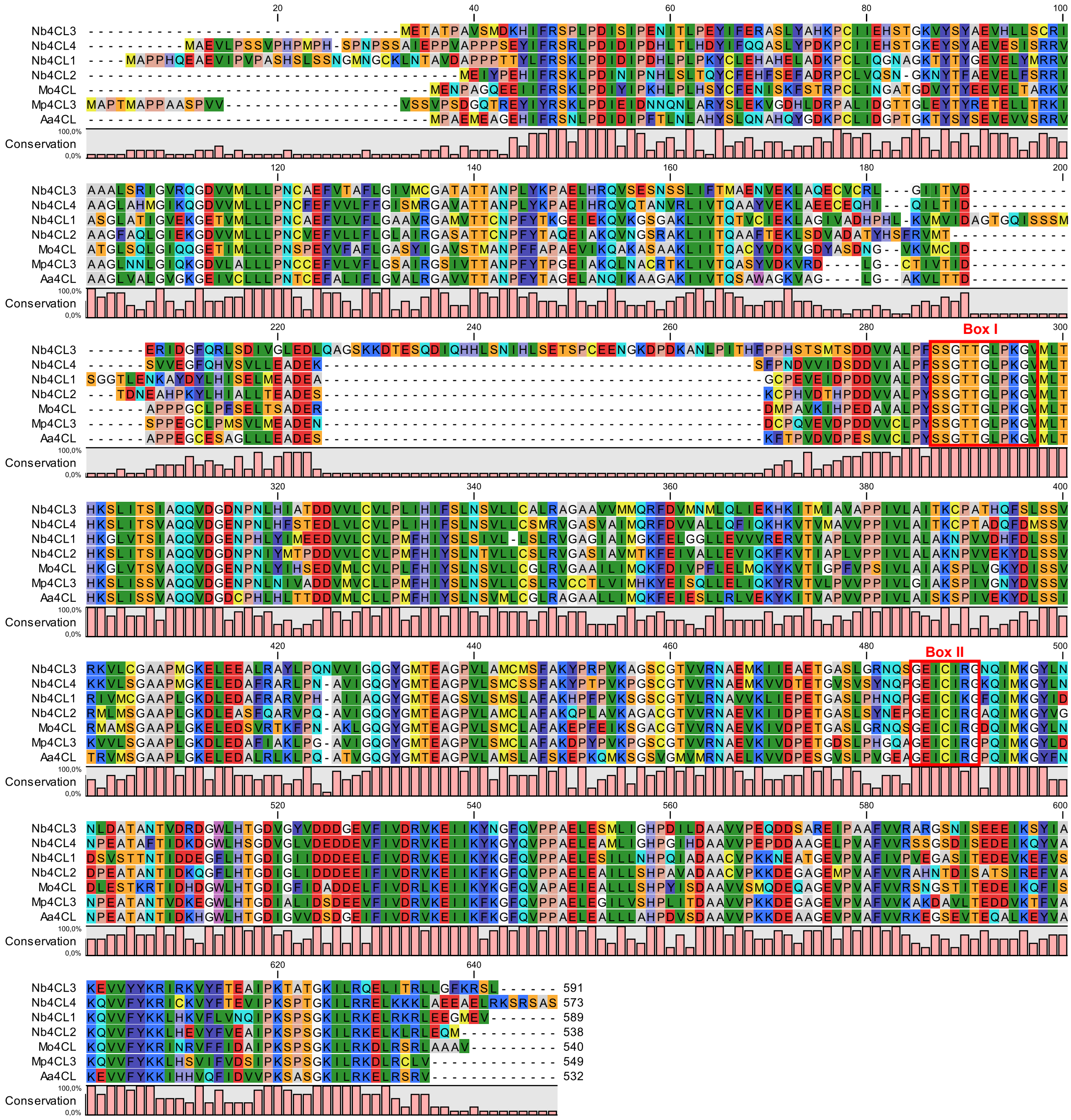

Fig. S24 Alignment of putative 4-coumarate coenzyme A-ligase (4CL) sequences from *Neoblechnum brasiliense* (Nb4CL1, PZ166765; Nb4CL2, PZ166766; Nb4CL3, PZ166767; Nb4CL4, PZ166768) with other plant 4CL sequences (*Marchantia polymorpha* 4CL3 (A0A2R6W0T8), *Anthoceros agrestis* (MN922305), *Melissa officinalis* (E1UYU5)). Conserved motifs are marked with red boxes (Schwarze and Petersen 2024). Alignment created with Clustal Omega (www.theclustal.com) and CLC sequence viewer 8.0.0

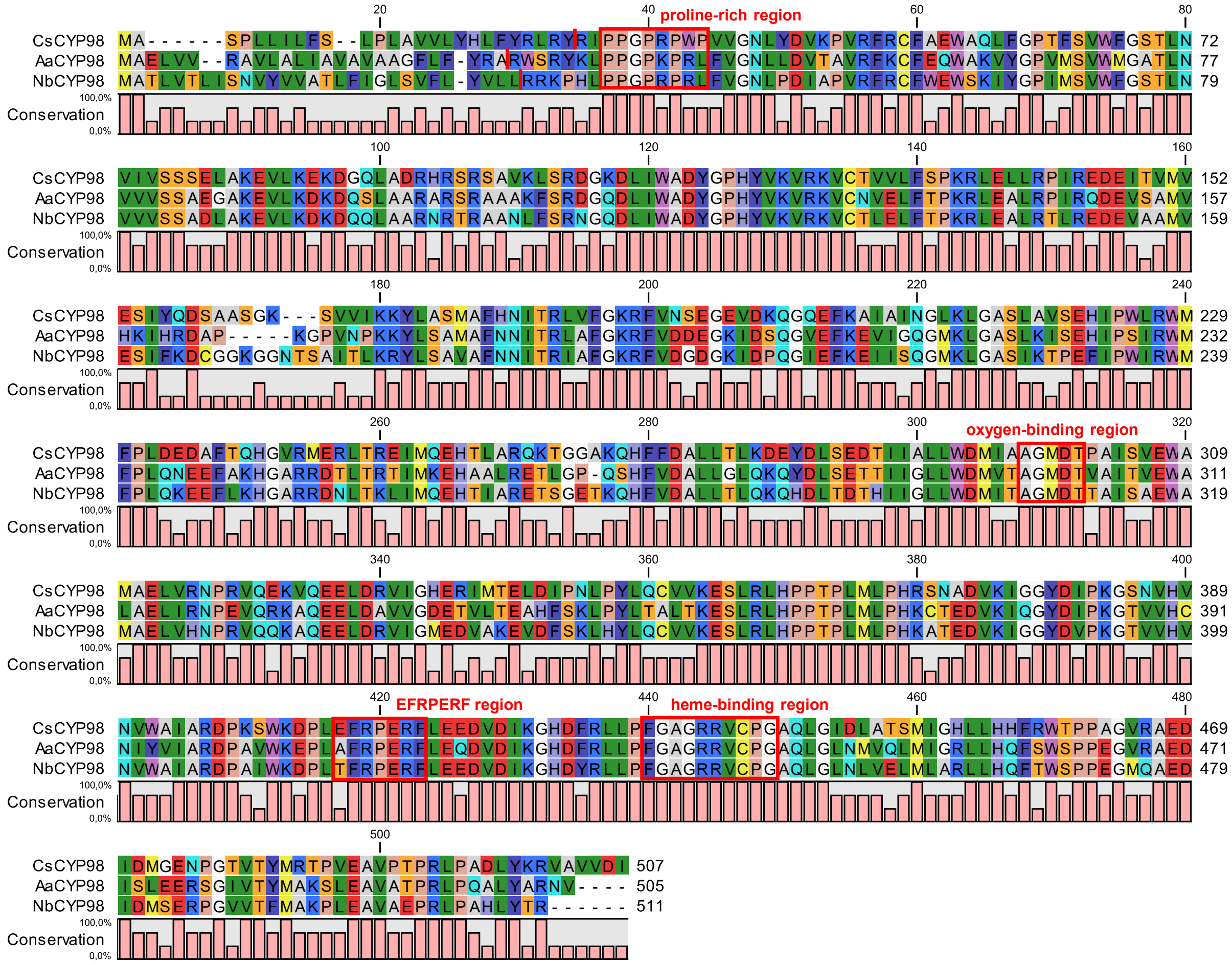

Fig. S25 Alignment of a putative 3-hydroxylase (CYP98) from *Neoblechnum brasiliense* (PZ166776) with other plant CYP98 sequences (*Coleus scutellarioides* (AJ427452), *Anthoceros agrestis* (MT119883)). Conserved motifs are marked with red boxes (Eberle et al. 2009). Transmembrane domains were predicted with TMHMM 2.0 (Krogh et al. 2001) and are indicated by red vertical lines. Alignment created with Clustal Omega (www.theclustal.com) and CLC sequence viewer 8.0.0

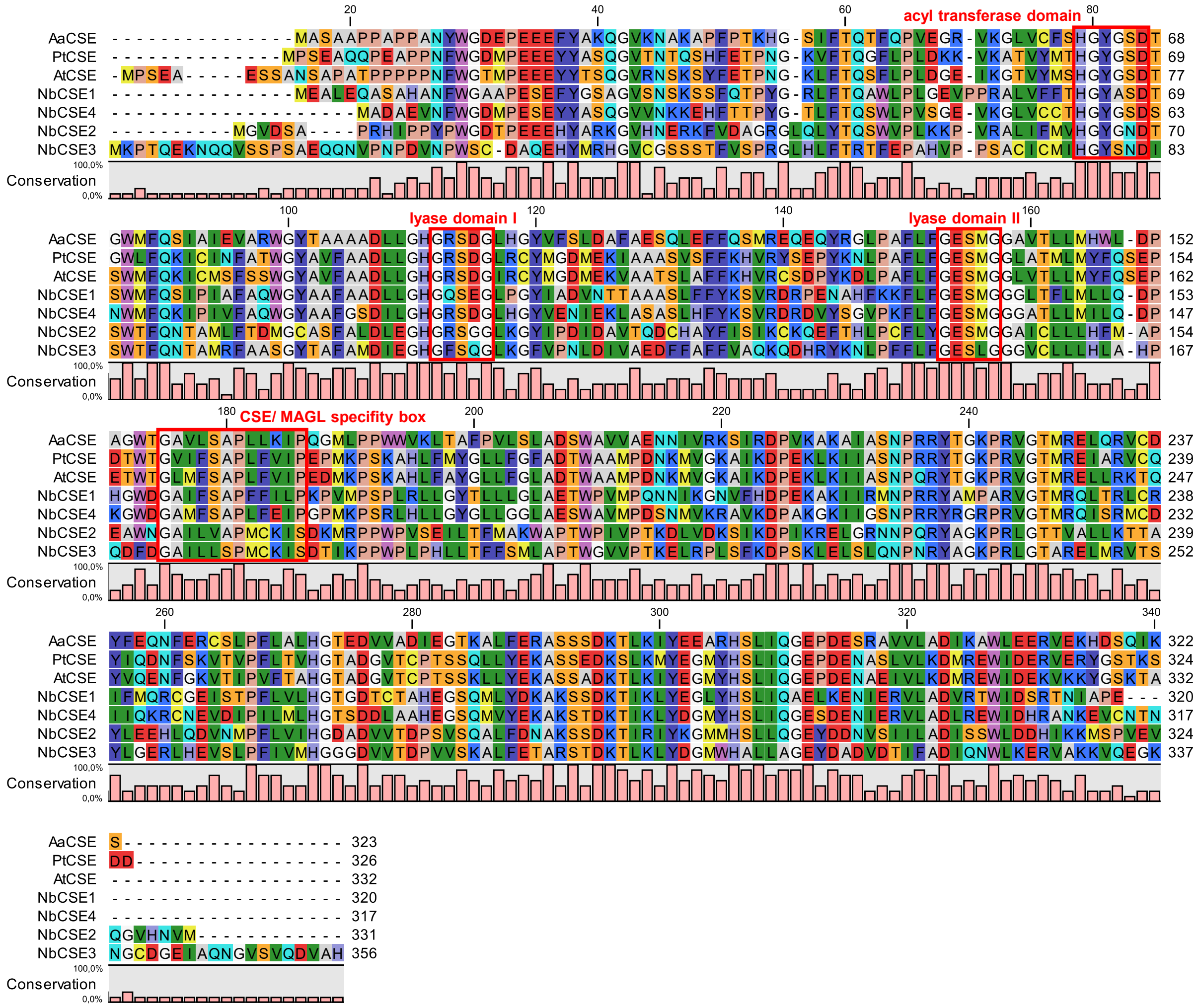

Fig. S26 Alignment of a putative caffeoylshikimate esterase (CSE) sequences from *Neoblechnum brasiliense* (NbCSE1, PZ166769; NbCSE2, PZ166770; NbCSE3, PZ166771; NbCSE4, PZ166772) with other plant CSE sequences (*Arabidopsis thaliana* (NP_175685.1), *Anthoceros agrestis* (PV962689), *Populus trichocarpa* (XP_002303266.1)). Conserved motifs are marked with red boxes (Wang et al. 2021). The CSE/MAGL specificity box indicates whether the enzyme has a putative MAGL (G A V/I L V/L A P M C K I A/S) or CSE (G L/A I/V/M F/L S A P L F/L/I V/K I/M P) activity (de Vries et al. 2021). Alignment created with Clustal Omega (www.theclustal.com) and CLC sequence viewer 8.0.0

Supplementary Tables

Table S1 Sequences used to create an HMM profile and in the phylogenetic tree (Fig. 3) in addition to the sequences retrieved from the novel transcriptome of *Neoblechnum brasiliense*. (**A**) Sequences described in Ufland and Petersen (2026), (**B**) further selected sequences, (**C**) sequences taken from the phylogenetic analysis of Kruse et al. (2022)

**Abbreviation**	**Genbank accession or source**	**Species**
(**A**) **characterised NbHCT sequences (Ufland and Petersen 2026)**		
NbHCT1	PV344582	*Neoblechnum brasiliense*
NbHCT2	PV344583	*Neoblechnum brasiliense*
NbHCT3	PV344584	*Neoblechnum brasiliense*
NbHCT4	PV344585	*Neoblechnum brasiliense*
NbHCT5	PV344586	*Neoblechnum brasiliense*
NbHCT6	PV344587	*Neoblechnum brasiliense*
NbHCT7	PV344588	*Neoblechnum brasiliense*
NbHCT8	PV344589	*Neoblechnum brasiliense*
NbHCT9	PV344590	*Neoblechnum brasiliense*
(**B**) **further selected sequences**		
Ecol_CAT3	CAT3_ECOLX	*Escherichia coli*
Ecol_CAT2	CAT2_ECOLX	*Escherichia coli*
Ecol_CAT1	CAT_ECOLX	*Escherichia coli*
GhRAS1	Sander 2010	*Glechoma hederacea*
GhRAS2	CDG56251.1	*Glechoma hederacea*
LaRAS	DQ886904	*Lavandula angustifolia*
MoRAS	G0LD36.1	*Melissa officinalis*
MlAT1	KAH6819558.1	*Mentha longifolia*
MlAT6	Zhou et al. 2024	*Mentha longifolia*
OtRAS	QGN67259.1	*Ocimum tenuiflorum*
PfRAS	KAH6771934.1	*Perilla frutescens* var. *frutescens*
PcRAS	QDF44407.1	*Phacelia campanularia*
CbRAS	CAK55166	*Coleus scutellarioides*
SmRAS	ADA60182.1	*Salvia miltiorrhiza*
SgRAS	PP449351	*Sarcandra glabra*
PP449349_SarglaHST	PP449349	*Sarcandra glabra*
PP449350_SarglaHQT1	PP449350	*Sarcandra glabra*
PP449352_SarglaHQT2	PP449352	*Sarcandra glabra*
(**C**) **sequences taken from the phylogenetic analysis of Kruse et al. (2022)**		
ArHPT1_QAA12830.1	QAA12830.1	*Actaea racemosa*
AsFMT_JA758320.1	JA758320.1	*Angelica sinensis*

G10_Aagr_000038l.49.1	Kruse et al. 2022	*Anthoceros agrestis*
At3AT1_NM_100275.3	NM_100275.3	*Arabidopsis thaliana*
At3AT2_NM_100232.5	NM_100232.5	*Arabidopsis thaliana*
AT3G47170_NP_190301.2	NP_190301.2	*Arabidopsis thaliana*
AT5G07080_OAO95042.1	OAO95042.1	*Arabidopsis thaliana*
At5Mat_NM_113880.2	NM_113880.2	*Arabidopsis thaliana*
AtACT_NM_125509.3	NM_125509.3	*Arabidopsis thaliana*
AtASFT_HHT1_AY062996.1	AY062996.1	*Arabidopsis thaliana*
AtBIA1_NP_193275.1_gen	NP_193275.1	*Arabidopsis thaliana*
AtCER2_AY087262_gen	AY087262	*Arabidopsis thaliana*
AtDCF_BT010448.1	BT010448.1	*Arabidopsis thaliana*
AtDCR_BT000770.1_gen	BT000770.1	*Arabidopsis thaliana*
AtEPS1_NM_126116.2	NM_126116.2	*Arabidopsis thaliana*
AtFACT_AY142537.1	AY142537.1	*Arabidopsis thaliana*
AtHCT_NM_124270.4	NM_124270.4	*Arabidopsis thaliana*
AtSCT_BT009686.1	BT009686.1	*Arabidopsis thaliana*
AtSDT_NM_127915.2	NM_127915.2	*Arabidopsis thaliana*
AtSHT_AT2G19070.1	AT2G19070.1	*Arabidopsis thaliana*
AtTHAA1_At5g47980	At5g47980	*Arabidopsis thaliana*
AtTHAA2_At5g47950	At5g47950	*Arabidopsis thaliana*
AsaHHT1_AB076980	AB076980	*Avena sativa*
BdPMT_HG421450.1_gen	HG421450.1	*Brachypodium distachyon*
CaPun1_NM_001324769_gen	NM_001324769	*Capsicum annuum*
CrDAT_AF053307	AF053307	*Catharanthus roseus*
CrMAT_AF253415	AF253415	*Catharanthus roseus*
CaAT20_MT277444	MT277444	*Celastrus angulatus*
Cbra_aGBG71897.1	GBG71897.1	*Chara braunii*
Cbra_bGBG71901.1	GBG71901.1	*Chara braunii*
Cbra_cGBG71902.1	GBG71902.1	*Chara braunii*
Cbra_dGBG76279.1	GBG76279.1	*Chara braunii*
G9_GBG76279.1_mod	GBG76279.1, Kruse et al. 2022	*Chara braunii*
CiHCT1_KT222891.1	KT222891.1	*Cichorium intybus*
CiHCT2_KT222892.1	KT222892.1	*Cichorium intybus*
CiHQT1_KT222893.1	KT222893.1	*Cichorium intybus*
CiHQT2_KT222894.1	KT222894.1	*Cichorium intybus*
CiHQT3_KT222895.1	KT222895.1	*Cichorium intybus*
CiSHT1_MG457243.1	MG457243.1	*Cichorium intybus*
CiSHT2_MG457244.1	MG457244.1	*Cichorium intybus*
CbBEAT_AF043464	AF043464	*Clarkia breweri*
CbBEBT_AF500200	AF500200	*Clarkia breweri*
CcaHCT_EF137954.1	EF137954.1	*Coffea canephora*

CcaHQT_EF153931.1	EF153931.1	*Coffea canephora*
CcAT1_AXB26761.1	AXB26761.1	*Crocosmia x crocosmiiflora*
CcAT2_AXB26762.1	AXB26761.1	*Crocosmia x crocosmiiflora*
CmAAT1_CAA94432.1	CAA94432.1	*Cucumis melo*
CmAAT3_AY859053	AY859053	*Cucumis melo*
CmAAT4_AY859054	AY859054	*Cucumis melo*
CsHCT_JN005932.1_gen	JN005932.1	*Cucumis sativus*
CcHQT1_AM690438.2	AM690438.2	*Cynara cardunculus*
CcHQT2_EU839580.1	EU839580.1	*Cynara cardunculus*
Dp3MAT_AF489108	AF489108	*Dahlia pinnata*
Dm3MAT1_AY298809	AY298809	*Dendranthema x morifolium*
Dm3MAT2_AY298810	AY298810	*Dendranthema x morifolium*
Dm3MAT3_BAF50706.1	BAF50706.1	*Dendranthema x morifolium*
DcHCBT2_Z84386	Z84386	*Dianthus caryophyllus*
EpHCT_MT936805	MT936805	*Echinacea purpurea*
EpHHT_MT936803	MT936803	*Echinacea purpurea*
EpHQT_MT936804	MT936804	*Echinacea purpurea*
EcBAHD8_KC140150.1	KC140150.1	*Erythroxylum coca*
EcCS_KC140149.1	KC140149.1	*Erythroxylum coca*
EcHQT_JQ413187.1	JQ413187.1	*Erythroxylum coca*
FaAAT_AF193789	AF193789	*Fragaria ananassa*
FvVAAT_AX025504	AX025504	*Fragaria vesca*
Gt5AT_AB010708	AB010708	*Gentiana triflora*
GmIF7MaT_NM_001250831.1	NM_001250831.1	*Glycine max*
GmIMaT1_KY399789.1	KY399789.1	*Glycine max*
GmIMaT3_KY399790.1	KY399790.1	*Glycine max*
GmMT7_EU192928.1	EU192928.1	*Glycine max*
HmACT_LC596950	LC596950	*Hordeum murinum*
HvACT1-1_BAF97626	BAF97626	*Hordeum vulgare*
HvACT1-2_BAF97627	BAF97627	*Hordeum vulgare*
HvACT2_BAK00935	BAK00935	*Hordeum vulgare*
HvACT_AY228552	AY228552	*Hordeum vulgare*
Ih3AT1_AB217625.1	AB217625.1	*Iris hollandica*
Ih3AT2_AB242298.1	AB242298.1	*Iris hollandica*
Kni_kfl00070_0280_v1.1	GAQ81091.1	*Klebsormidium nitens*
Kni_kfl00302_0150_v1.1	GAQ86655.1	*Klebsormidium nitens*
Kni_kfl00513_0110_v1.1	GAQ89351.1	*Klebsormidium nitens*
Lp3MAT1_AY500352	AY500352	*Lamium purpureum*
LanAAT1_DQ886904.1	DQ886904.1	*Lavandula angustifolia*
LanAAT2_DQ886905.1	DQ886905.1	*Lavandula angustifolia*
LeAAT1_LC520138	LC520138	*Lithospermum erythrorhizon*
LeSAT1_LC520137	LC520137	*Lithospermum erythrorhizon*

LaHMT_HLT_AB181292	HLT_AB181292	*Lupinus albus*
MdAAT1_AY707098	AY707098	*Malus domestica*
MdAAT2_AY517491.1_gen	AY517491.1	*Malus domestica*
MeHFT_MeHFT	MeHFT	*Marchantia emarginata*
G1_Mpol_Mapoly0005s0261.1.p	Mapoly0005s0261	*Marchantia polymorpha*
G8_MpolHCT_Mapoly0003s0277.1.p	Mapoly0003s0277, Kruse et al. 2022	*Marchantia polymorpha*
MpolFHT_Mapoly0157s0023.1.p	Mapoly0003s0277	*Marchantia polymorpha*
MpolHCT_Mapoly0003s0277.1.p	Mapoly0003s0277	*Marchantia polymorpha*
MtMaT1_EU272030.1	EU272030.1	*Medicago truncatula*
MtMaT2_EU272032.1	EU272032.1	*Medicago truncatula*
MtMaT3_EU272031.1	EU272031.1	*Medicago truncatula*
MoRAS_FR670523.1	FR670523.1	*Melissa officinales*
Mpus_123940	Kruse et al. 2022	*Micromonas pusilla*
MsAAT_AX025506	AX025506	*Musa sapientum*
NaAT1_JN390826.1	JN390826.1	*Nicotiana attenuata*
NaCV86_JN390825.1_gen	JN390825.1	*Nicotiana attenuata*
NaDH29_JN390824.1	JN390824.1	*Nicotiana attenuata*
NtBEBT_AF500202	AF500202	*Nicotiana tabacum*
NtHCT_AJ507825	AJ507825	*Nicotiana tabacum*
NtHQT_AJ582651	AJ582651	*Nicotiana tabacum*
NtMAT1_AB176525	AB176525	*Nicotiana tabacum*
NtSHT_MN787045	MN787045	*Nicotiana tabacum*
ObCAAT1_MN031888	MN031888	*Ocimum basilicum*
ObCAAT2_MN031889	MN031889	*Ocimum basilicum*
Os09g0544000_XP_015651357	XP_015651357	*Oryza sativa*
OsAHT1_ANQ47369	ANQ47369	*Oryza sativa*
OsHCT_XM_015786263.1	XM_015786263.1	*Oryza sativa*
OsPHT3_Os09g37180.1	Os09g37180.1	*Oryza sativa*
OsPHT4_Os09g37200	Os09g37200	*Oryza sativa*
OsPMT_XM_015765814.1	XM_015765814.1	*Oryza sativa*
OsSAT1_gen	Q2QRK9.1	*Oryza sativa*
OsSAT2_XM_015763244.1_gen	XM_015763244.1	*Oryza sativa*
OsTBT1_Os11g42290	Os11g42290	*Oryza sativa*
OsTBT2_Os11g42370	Os11g42370	*Oryza sativa*
OsTHT1_AK287765.1	AK287765.1	*Oryza sativa*
OsTHT2_XM_015757482.1	XM_015757482.1	*Oryza sativa*
Oluc_28702	Kruse et al. 2022	*Ostreococcus lucimarinus*
PvHCT1a_AB723827.1	AB723827.1	*Panicum virgatum*
PvHCT2a_KC696573.1	KC696573.1	*Panicum virgatum*
PvHCTLike1_AFY17066.1	AFY17066.1	*Panicum virgatum*
PsSALAT_AF339913	AF339913	*Papaver somniferum*

Pc3MAT_AY190121	AY190121	*Pericallis cruenta*
Pf3AT_AB029340	AB029340	*Perilla frutescens*
PaxASAT1_KT716258.1	KT716258.1	*Petunia axillaris*
PaxASAT2_KT716259.1	KT716259.1	*Petunia axillaris*
PaxASAT3_KT716260.1	KT716260.1	*Petunia axillaris*
PaxASAT4_KT716261.1	KT716261.1	*Petunia axillaris*
PaxCFAT_DQ767969.1	DQ767969.1	*Petunia axillaris*
PhBPBT_AY611496	AY611496	*Petunia x hybrida*
PvHHHT_KX443573.1	KX443573.1	*Phaseolus vulgaris*
G2_Ppat_Pp3c3_250V3.1.p	Kruse et al. 2022	*Physcomitrium patens*
G3_Ppat_Pp3c14_6190V3.1.p	Kruse et al. 2022	*Physcomitrium patens*
PpHCT1_KU513965.1	KU513965.1	*Physcomitrium patens*
PrHCT_EF121452.1	EF121452.1	*Pinus radiata*
PnPAS_MW354957	MW354956	*Piper nigrum*
PnPS_MW354956	MW354957	*Piper nigrum*
PsHCT2_FN647681.1	FN647681.1	*Plectranthus scutellarioides* (syn. *Coleus scutellarioides*)
PsRAS_AM283092.1	AM283092.1	*Plectranthus scutellarioides* (syn. *Coleus scutellarioides*)
PtHCT6_XM_006368430.2	XM_006368430.2	*Populus tremuloides*
PtBEBT_KP228019.1	KP228019.1	*Populus trichocarpa*
PtFHT1_JX515962.1	JX515962.1	*Populus trichocarpa*
PtHCT1_EU603313.1	EU603313.1	*Populus trichocarpa*
PtSABT_KP228018.1	KP228018.1	*Populus trichocarpa*
PpAAT1_MH700174	MH700174	*Prunus persica*
RsACT_AJ556780	AJ556780	*Rauvolfia serpentina*
RicSHT_MN787043	MN787043	*Ricinus communis*
RocSHT_MN787046	MN787046	*Rosa canina*
RhAAT1_AY850287	AY850287	*Rosa hybrida*
SsiASAT1_KY978746.1	KY978746.1	*Salpiglossis sinuata*
SsiASAT2_KY978747.1	KY978747.1	*Salpiglossis sinuata*
SsiASAT3_KY978748.1	KY978748.1	*Salpiglossis sinuata*
SsiASAT5_KY978749.1	KY978749.1	*Salpiglossis sinuata*
Ss5MAT1_AF405707	AF405707	*Salvia splendens*
Ss5MAT2_AY383734	AY383734	*Salvia splendens*
SmHCT1a_XM_002979015.2	XM_002979015.2	*Selaginella moellendorffii*
ShASAT2_KT359561.1	KT359561.1	*Solanum habrochaites*
SlAAT1_KM975322.1	KM975322.1	*Solanum lycopersicum*
SlACT1_Solyc11g071470.1	Solyc11g071470	*Solanum lycopersicum*
SlACT2_Solyc11g071480.1.1	Solyc11g071480	*Solanum lycopersicum*
SlASAT1_NM_001329403.1	NM_001329403.1	*Solanum lycopersicum*
SlASAT2_NM_001329392.1	NM_001329392.1	*Solanum lycopersicum*

SlASAT3	Solyc11g067270	*Solanum lycopersicum*
SlASAT4	Solyc01g105580	*Solanum lycopersicum*
SlHCT_Solyc03g117600.2.1	Solyc03g117600	*Solanum lycopersicum*
SlHQT_AJ582652.1	AJ582652.1	*Solanum lycopersicum*
SlHQT_Solyc07g005760.2.1	Solyc07g005760	*Solanum lycopersicum*
SlSHT_MN787044	MN787044	*Solanum lycopersicum*
SmSpmHT_AKP24035.1	AKP24035.1	*Solanum melongena*
SpAAT1_KM975321.1	KM975321.1	*Solanum pennellii*
SqTAITAT_MT024677	MT024677	*Solanum quitoense*
SrSpmHT_AMB21755.1	AMB21755.1	*Solanum richardii*
StFHT_NM_001288261.1	NM_001288261.1	*Solanum tuberosum*
SbHCT_XM_002452390.2	XM_002452390.2	*Sorghum bicolor*
G4_Sfal_Sphfalx02G197500.1.p	Sphfalx02G197500 Kruse et al. 2022	*Sphagnum fallax*
G5_Sfal_Sphfalx08G078400.1.p	Sphfalx08G078400 Kruse et al. 2022	*Sphagnum fallax*
G6_Sfal_Sphfalx09G052100.1.p	Sphfalx09G052100 Kruse et al. 2022	*Sphagnum fallax*
G7_Sfal_Sphfalx12G001300.1.p	Sphfalx12G001300 Kruse et al. 2022	*Sphagnum fallax*
SoSOAP10_KNA12459.1	KNA12459.1	*Spinacia oleracea*
TcBAPT_AY082804	AY082804	*Taxus cuspidata*
TcDBAT_AF193765	AF193765	*Taxus cuspidata*
TcDBBT_AF297618	AF297618	*Taxus cuspidata*
TcDBNTBT_AF466397	AF466397	*Taxus cuspidata*
TcTAT_AF190130	AF190130	*Taxus cuspidata*
TfHCT1A_EU861218.1	EU861218.1	*Trifolium pratense*
TfHCT1B_FJ151489.1	FJ151489.1	*Trifolium pratense*
TfHCT2_EU861219.1	EU861219.1	*Trifolium pratense*
TaACT1-1_LC596951	LC596951	*Triticum aestivum*
TaACT1-2_LC596952	LC596952	*Triticum aestivum*
Vh3MAT1_AY500350	AY500350	*Verbena hybrida*
VlAMAT_AY705388	AY705388	*Vitis labrusca*
Vv3AT_KM267559.1	KM267559.1	*Vitis vinifera*
VvSHT_MN787047	MN787047	*Vitis vinifera*
ZmAat1_NP_001148286.2	NP_001148286.2	*Zea mays*
ZmGlossy2_Q41809	Q41809	*Zea mays*
ZmpCAT_BT042717.1	BT042717.1	*Zea mays*

Table S2 PCR primers for full-length amplification. Due to gene renaming, the name of a primer may not always match the name of the gene it amplifies. The melting temperature is only given for the binding part of the primer. Underlined: non-binding part, blue: overhang with expression vector for homologous recombination, red: restriction site, yellow: added bases

Primer name	Sequence (5' → 3')	Length [bp]	T_m [°C]	Restriction enzyme
**Full-length PCR primers for *NbHCT10***				
NbHCT-J-HR-fw*	GCCGCGCGGCAGCCATATGGTGAAGGTGCACATTCTCTCCTCTGAG	46	68.1	NdeI
NbHCT-J-HR-rv*	TAGCAGCCGGATCCTCGAGCTAGTTGTGGTTAAGATGCAAAATGAGCTCTTTGAACTTAG	60	68.4	XhoI
**Full-length PCR primers for *NbHCT11***				
NbHCT-M-HR-fw*	GCCGCGCGGCAGCCATATGATATTGAGTGTGAGATCAGTAGCGTCCCAATTAGTAAA	57	68.4	NdeI
NbHCT-M-HR-rv*	TAGCAGCCGGATCCTCGAGTCATGTAGAATCAAGCAAGAGGATTTGGGAAAATTTATACATGTG	64	68.5	XhoI
**Full-length PCR primers for *NbHCT12***				
NbHCT3-HR-fw*	GCCGCGCGGCAGCCATATGATAATGACTGTGAGCTCAGTGGCTTCTCAATTAG	53	68.4	NdeI
NbHCT3-HR-rv*	TAGCAGCCGGATCCTCGAGTTAAACCCTTGTTAAGCGTGACATCTCAATATTGAGGAG	58	68.4	XhoI
**Full-length PCR primers for *NbHCT13***				
NbHCT-G-HR-fw*	GCCGCGCGGCAGCCATATGATACTCACTGTGAGGTCAGTGGCATCTCAATT	51	68.3	NdeI
NbHCT-G-HR-rv*	TAGCAGCCGGATCCTCGAGTCAAAAATCAAGCAAGAGATGGGAGAATTTTTGCATGTGG	59	68.4	XhoI
**Full-length PCR primers for *NbHCT14***				
NbHCT-27-HR-fv*	GCCGCGCGGCAGCCATATGCACATCATGCACAGGATCGAGGATACC	46	68.1	NdeI
NbHCT-27-HR-rv*	TAGCAGCCGGATCCTCGAGTTATGGCTTAATGAGGGCTTCAAATTGTACCATGTGC	56	68.4	XhoI
**Full-length PCR primers for *NbHCT15***				
NbHCT-41-HR-fv*	GCCGCGCGGCAGCCATATGCACATCCTACGCAAGTCACAGGAGATAATATAC	52	68.3	NdeI
NbHCT-41-HR-rv*	TAGCAGCCGGATCCTCGAGCTAGTACAAAGGTCTTTGTGACATGAGAGATTCAAGTTTATTC	62	68.5	XhoI
**Full-length PCR primers for *NbHCT16***				
NbHCT-28-HR-fv*	GCCGCGCGGCAGCCATATGGGGGCGGAAGACATAACTGTGCATTC	45	68.1	NdeI
NbHCT-28-HR-rv*	TAGCAGCCGGATCCTCGAGCTATGCATCAGTGGCCCTCAATTCAGGTAC	49	68.1	XhoI
**Full-length PCR primers for *NbHCT17***				
NbHCT-I-HR-fv*	GCCGCGCGGCAGCCATATGGGCTCAGCACCACCTCCCAGC	40	69.6	NdeI

Primer name	Sequence (5' → 3')	Length [bp]	T_m [°C]	Restriction enzyme
NbHCT-I-HR-rv*	TAGCAGCCGGATCCTCGAGTTAGAAGTTGTAGATGAGCTTTTCAAATTGTTGCATATGGGAAG	63	68.5	XhoI
**Full-length PCR primers for *NbHCT18***				
NbHCT-14-HR-fv*	GCCGCGCGGCAGCCATATGAGTACTGTCAATGGGCATGGAGGGC	44	68.1	NdeI
NbHCT-14-HR-rv*	TAGCAGCCGGATCCTCGAGCTAGTTATCATAAATGAGCTTCTCAAACTTGTCCATTTGATGAG	63	68.5	XhoI
**Full-length PCR primers for *NbHCT19***				
NbHCT-7-HR-fv*	GCCGCGCGGCAGCCATATGGGTGCAGAAGCCCCACACTCACAG	43	69.5	NdeI
NbHCT-7-HR-rv*	TAGCAGCCGGATCCTCGAGTTACATAAGCTCAGGCTGCAAGATCTCTTCAAACTTATG	58	68.4	XhoI
**Full-length PCR primers for *NbHCT20***				
NbHCT-8-HR-fv*	GCCGCGCGGCAGCCATATGGGTGCTGGAGCTGCTCCTCCG	40	69.6	NdeI
NbHCT-8-HR-rv*	TAGCAGCCGGATCCTCGAGCTAGAGTTGGGGTTGAATGAGCTGATCAAAGG	51	68.2	XhoI
**Full-length PCR primers for *NbHCT21***				
NbHCT-L-HR-fv*	GCCGCGCGGCAGCCATATGCTCACATTGGAGCCTGTGAGGATGGTGAATATG	52	70.6	NdeI
NbHCT-L-HR-rv*	TAGCAGCCGGATCCTCGAGCTAGCGTATGTACTCTGCAATTTCTTCTCGGAAG	53	68.3	XhoI
**Full-length PCR primers for *NbHCT22***				
NbHCT-K-HR-fv*	GCCGCGCGGCAGCCATATGCTCACATTAGAGCCTGTGAGGATGGTAAATATG	52	68.3	NdeI
NbHCT-K-HR-rv*	TAGCAGCCGGATCCTCGAGCTAGCTAATATACGCTGCAATTTCTTCTTGGAAGGAC	56	68.4	XhoI
**Full-length PCR primers for *NbHCT23***				
NbHCT-25-HR-fv*	GCCGCGCGGCAGCCATATGGAAGTATTTTGCGAGACTCCTATTTTCGTGAAGG	53	68.4	NdeI
NbHCT-25-HR-rv*	TAGCAGCCGGATCCTCGAGTCACAAATTAAGGGCATCTTGGAACTTTTGAAGCTCG	56	68.4	XhoI
**Full-length PCR primers for *NbHCT24***				
NbHCT-42-HR-fv*	GCCGCGCGGCAGCCATATGGAGAGAAAAGCAACGCCTATGTTGGTACAG	49	68.2	NdeI
NbHCT-42-HR-rv*	TAGCAGCCGGATCCTCGAGCTACATTCGGCTATCCTTGGACATGTAGGC	49	68.1	XhoI
**Full-length PCR primers for *NbHCT25***				
NbHCT-36-HR-fv*	GCCGCGCGGCAGCCATATGGATTCTTACAAAAGCCCCTATGAGCCCC	47	68.2	NdeI
NbHCT-36-HR-rv*	TAGCAGCCGGATCCTCGAGTCAAAGCCTACTATCAGAAAGGTGACGTGTAGG	52	68.2	XhoI
**Full-length PCR primers for *NbHCT26***				

Primer name	Sequence (5' → 3')	Length [bp]	T_m [°C]	Restriction enzyme
NbHCT-F-HR-fv*	GCCGCGCGGCAGCCATATGGCCAATTTTCATGTCAAAGA AGAGAACGTCTGC	52	68.3	NdeI
NbHCT-F-HR-rv*	TAGCAGCCGGATCCTCGAGTTAATCAAGAATATAATGAGC AAACTTCTCCATGTTGTGAGGC	62	68.5	XhoI
**Full-length PCR primers for *NbHCT27***				
NbHCT-P-HR-fv*	GCCGCGCGGCAGCCATATGGCGATCCATATCCAGGAGAC TTGTTTGG	47	68.2	NdeI
NbHCT-P-HR-rv*	TAGCAGCCGGATCCTCGAGTCAAGTGGAGATGTCGGAGA GAAGACACG	48	68.1	XhoI
**Full-length PCR primers for *NbHCT28***				
NbHCT-Q-HR-fv*	GCCGCGCGGCAGCCATATGGGAGACCTGCCGAATCACTC ACAG	43	68.0	NdeI
NbHCT-Q-HR-rv*	TAGCAGCCGGATCCTCGAGTCATGTGCAAAAAGGAAAAG AAGATTTGCAAAGGGGATC	58	68.4	XhoI
**Full-length PCR primers for *NbHCT29***				
NbHCT-R-HR-fv*	GCCGCGCGGCAGCCATATGCCATCTGCAAGCGTGCACAT CCTTG	44	68.1	NdeI
NbHCT-R-HR-rv*	TAGCAGCCGGATCCTCGAGTCATATGTCAACACAAGGGC ATGGGATGGAG	50	68.2	XhoI
**Full-length PCR primers for *NbHCT30***				
NbHCT-35-HR-fv*	GCCGCGCGGCAGCCATATGGGCATGGAAGAGGGAGTTA GTGTGG	44	68.1	NdeI
NbHCT-35-HR-rv*	TAGCAGCCGGATCCTCGAGTCAGCAAGCGAGGAAGGCA GGGTCG	44	69.5	XhoI
**Full-length PCR primers for *NbHCT31***				
NbHCT-24-HR-fv*	GCCGCGCGGCAGCCATATGGCAGCAGTAGATGCCATGA GCGAG	43	68.0	NdeI
NbHCT-24-HR-rv*	TAGCAGCCGGATCCTCGAGCTATCTTAGAAACAAAGCCAT GTTAAAAACGTAGGTGCTTG	60	68.4	XhoI
**Full-length PCR primers for *NbHCT32***				
NbHCT-A-HR-fw*	GCCGCGCGGCAGCCATATGGCAAAAGTAACGATTTTAGA GAGGCACATGGTG	52	68.3	NdeI
NbHCT-A-HR-rv*	TAGCAGCCGGATCCTCGAGCTAAGGGTTGATAAAAGCAT CGTCACTTGCAAGC	53	68.3	XhoI
**Full-length PCR primers for *NbHCT33***				
NbHCT-C-HR-fw*	GCCGCGCGGCAGCCATATGGAGAACGTAGAGATAGAAAT CACGCACACTTC	51	68.3	NdeI
NbHCT-C-HR-rv*	TAGCAGCCGGATCCTCGAGTCAATGTTGGTCAATATTTAC AAGAAAAGATGGATCACTTTTAAGCTG	67	68.6	XhoI
**Full-length PCR primers or *NbHCT34***				
NbHCT-B-HR-fw*	GCCGCGCGGCAGCCATATGGAGAACGTAGAAATAGACAT AATACACACTGCTCTC	55	68.4	NdeI

Primer name	Sequence (5' → 3')	Length [bp]	T_m [°C]	Restriction enzyme
NbHCT-B-HR-rv*	TAGCAGCCGGATCCTCGAGTTAACTTAGATCCAAGAGAAACGATGGGTCACTTTCAAG	58	68.4	XhoI
**Full-length PCR primers r for *NbHCT35***				
NbHCT-D-HR-fv*	GCCGCGCGGCAGCCATATGGCATCATGGGCGCCCGCAATCACTACAG	47	72.1	NdeI
NbHCT-D-HR-rv*	TAGCAGCCGGATCCTCGAGTCAGAAGGATAAGAAGCCTTCATCTTGTTCCAATCTTTCCATGACATC	67	72.0	XhoI
**Full-length PCR primers for *NbHCT36***				
NbHCT-19-HR-fv*	GCCGCGCGGCAGCCATATGGCATCATGGGCGCCCGCAATCACTACGC	47	73.5	NdeI
NbHCT-19-HR-rv*	TAGCAGCCGGATCCTCGAGTTAGAAGGATAAGAAGCCTTCATCTTGTTCCAATCTTTTCATGACAGG	67	71.1	XhoI
**Full-length PCR primers for *NbHCT37***				
NbHCT-30-HR-fv*	GCCGCGCGGCAGCCATATGGCTGATTCAAGGGTATGCGAAGGAGG	45	68.1	NdeI
NbHCT-30-HR-rv*	TAGCAGCCGGATCCTCGAGTTATTCATAGTCAAAGGGATTACAAGGGGGTATCCC	55	68.3	XhoI
**Full-length PCR primers for *NbHCT38***				
NbHCT-34-HR-fv*	GCCGCGCGGCAGCCATATGGCTAATTCAAGGGTATGTGAAGTAGGGGAG	49	68.2	NdeI
NbHCT-34-HR-rv*	TAGCAGCCGGATCCTCGAGCTAATCCTCAAAGGGATTACAAGGGGGTTTCC	51	68.2	XhoI
**Full-length PCR primers for *NbHCT39***				
NbHCT-37-HR-fv*	GCCGCGCGGCAGCCATATGGGTAATGTAAGGGTACGTGAAGGAGGG	46	68.1	NdeI
NbHCT-37-HR-rv*	TAGCAGCCGGATCCTCGAGTTAAGGGAGTTTCCCTAAAAATGAGGGATCTTCAAAAAGATG	61	68.5	XhoI
**Full-length PCR primers for *NbHCT40***				
NbHCT-N-HR-fv*	GCCGCGCGGCAGCCATATGGCTGATTCAATTAGGCAGGTGAGCGTG	46	68.1	NdeI
NbHCT-N-HR-rv*	TAGCAGCCGGATCCTCGAGCTAGTCATTAGCCAAAGGACTAGAAGGGGG	49	68.1	XhoI
**Full-length PCR primers for *NbHCT41***				
NbHCT-O-HR-fv*	GCCGCGCGGCAGCCATATGGGTGGTTCAATTAGGCAGGTGAGCG	44	68.1	NdeI
NbHCT-O-HR-rv*	TAGCAGCCGGATCCTCGAGCTAGTCATTAGCCAGAGGAATAGAAGGGGG	49	68.1	XhoI
**Full-length PCR primers for *NbHCT42***				
NbHCT-E-HR-fv*	GCCGCGCGGCAGCCATATGGGCGATACCGAAGGGCATGTGAG	42	68.0	NdeI
NbHCT-E-HR-rv*	TAGCAGCCGGATCCTCGAGCTATTTGCTAGCAAAAGGACTAGAAGGCGGTTTAG	54	68.3	XhoI
**Full-length PCR primers for *NbHCT43***				

Primer name	Sequence (5' → 3')	Length [bp]	T_m [°C]	Restriction enzyme
NbHCT-16-HR-fv*	GCCGCGCGGCAGCCATATGGGGGAATTGGGCGAGCAAG TGAAG	43	68.0	NdeI
NbHCT-16-HR-rv*	TAGCAGCCGGATCCTCGAGCTATTCGTTAGCAAAAGGACT ACAAGGCGGTTTAG	54	68.3	XhoI
**Full-length PCR primers for *NbHCT44***				
NbHCT-31-HR-fv*	GCCGCGCGGCAGCCATATGCTTGGAAAATTGGGCTATGA TGAGGCGC	47	68.2	NdeI
NbHCT-31-HR-rv*	TAGCAGCCGGATCCTCGAGCTAAAGTGAAATCTTAGATGT AGCTAATGGAGGATGAGC	58	68.4	XhoI
**Full-length PCR primers for *NbHCT45***				
NbHCT-20-HR-fv*	GCCGCGCGGCAGCCATATGGTCACCATAGTGAAGACAAG CACAATAGTACC	51	68.3	NdeI
NbHCT-20-HR-rv*	TAGCAGCCGGATCCTCGAGCTAGCATCCATTGCTAGGGA AAAGGAAGAGG	50	68.2	XhoI
**Full-length PCR primers for *NbHCT46***				
NbHCT-22-HR-fv*	GCCGCGCGGCAGCCATATGTCAATTGAAAGGGTGACCAT GCTAAAGACGAG	51	68.3	NdeI
NbHCT-22-HR-rv*	TAGCAGCCGGATCCTCGAGTTAAGGATTGAGAAACTTAG GATCATCAATCAAGTGTTGGAG	61	68.5	XhoI
**Full-length PCR primers for *NbHCT47***				
NbHCT-29-HR-fv*	GCCGCGCGGCAGCCATATGGATGAGAGCGATGAGGTTAC CATTGTTGAG	49	68.2	NdeI
NbHCT-29-HR-rv*	TAGCAGCCGGATCCTCGAGTCATGAGATGCATGGAGAGG GGTCCTTG	47	68.1	XhoI
**Full-length PCR primer for *NbCYP98A268***				
BbCYP98-VLf*	TAGAATTCATGGCAACACTTGTTACATTGATCTC	34	60.1	EcoR I
BbCYP98-VLr*	TAGCGGCCGCCCTTGTATATAGATGGGCAGGC	32	60.3	NotI
NbCYP98co-HR-fw*	TTTTTGAAAATTCGAATTCGCAATAATGGCTACATTGGTTA CATTAATCTCTAACG	56	68.5	EcoR I
NbCYP98co-HR-rv*	TCGATACTAGTGCGGCCGCTCTAGTATACAAATGTGCTG	39	68.1	NotI

Table S3 Substrates applied in BAHD acyltransferase assays, concentration of the stock solutions and preparation methods for LC/ESI-MS/MS analysis. The term "aliphatic or aromatic alcohol/amine" refers to the carbon atom that bears an alcohol or amine group. Adapted from (Ufland and Petersen 2026)

**Substrate**	**Stock solution used in assays**	**Method of preparation for LC/ESI-MS/MS**	**Ionization**
Acyl acceptors – shikimic acid pathway derivatives			
quinic acid	1 M pH 7.0	method A	negative mode
shikimic acid	1 M pH 7.0	method A	negative mode
chlorogenic acid + quinic acid	25 mM<br>+ 1 M pH 7.0	method A	negative mode
Acyl acceptors – 4-hydroxyphenyllactic acid derivatives and precursors			
(*R*/*S*)-3-phenyllactic acid	1 M pH 7.0	method A	negative mode
(*RS*/*S*)-4-hydroxyphenyllactic acid	100 mM	method A	negative mode
(*RS*)-3,4-dihydroxyphenyllactic acid	100 mM	method A	negative mode
D/L-phenylalanine	100 mM	method A	positive mode
D/L-tyrosine	100 mM	method B	positive mode
DL-DOPA	100 mM	method B	positive mode
piscidic acid	38 mM	method A	negative mode
Acyl acceptors – aliphatic alcohols			
glycerol	100 mM	method A	positive mode
methanol	100 mM	method A	positive mode
ethanol	100 mM	method A	positive mode
iso-propanol	100 mM	method A	positive mode
n-propanol	100 mM	method A	positive mode
butanol	100 mM	method A	positive mode
ω-hydroxypalmitic acid	50 mM pH 7.3<br>10 % ethanol	Method A	negative mode
coniferyl alcohol	100 mM	method A	positive mode
benzyl alcohol	1 M pH 7.0	method A	positive mode
4-hydroxybenzyl alcohol	100 mM	method A	positive mode
4-isopropylbenzyl alcohol	100 mM	method A	positive mode
1-phenylethanol	100 mM	method A	positive mode
2-phenylethanol	undiluted	method A	positive mode
2-(4-hydroxyphenyl)-ethanol	100 mM	method A	positive mode
3-phenylpropanol	pure	method A	positive mode
3-(4-hydroxyphenyl)-propanol	100 mM	method A	positive mode

Substrate	Stock solution used in assays	Method of preparation for LC/ESI-MS/MS	Ionization
4-phenylbutanol	pure	method A	positive mode
D-glucose	1 M pH 7.0	method A	positive mode
D-galactose	1 M pH 7.0	method A	positive mode
D/L-threonic acid	10 mM	method A	negative mode
mucic acid	10 mM	method A	negative mode
D-glucaric acid	100 mM	method A	negative mode
D/L-tartaric acid	100 mM	method A	negative mode
meso-tartaric acid	100 mM	method A	negative mode
D/L-malic acid	100 mM	method A	negative mode
Acyl acceptors – aromatic alcohols and amines			
2-hydroxybenzoic acid	100 mM	method A	negative mode
3-hydroxybenzoic acid	100 mM	method A	negative mode
4-hydroxybenzoic acid	100 mM	method A	negative mode
2-aminobenzoic acid	100 mM	method A	negative mode
3-aminobenzoic acid	100 mM	method A	negative mode
4-aminobenzoic acid	100 mM	method A	negative mode
2,3-dihydroxybenzoic acid	100 mM	method A	negative mode
2,4-dihydroxybenzoic acid	100 mM	method A	negative mode
2,5-dihydroxybenzoic acid	100 mM	method A	negative mode
3,4-dihydroxybenzoic acid	100 mM	method A	negative mode
3-hydroxyanthranilic acid	75 mM pH 7.0	method A	negative mode
5-hydroxyanthranilic acid	75 mM pH 7.0	method A	negative mode
3-aminosalicylic acid	100 mM	method A	negative mode
vanillic acid	100 mM	method A	negative mode
homogentisic acid	100 mM	method A	negative mode
4-hydroxyphenylacetic acid	100 mM	method A	negative mode
Acyl acceptors – aliphatic amines			
putrescine	96 mM pH 7.0	method B	positive mode
spermidine	63.5 mM pH 7.0	method B	positive mode
spermine	9.19 mM pH 7.0	method B	positive mode
agmatine	96.4 mM pH 7.0	method B	positive mode
2-phenylethylamine	20 mM	method B	positive mode
tyramine	9.5 mM pH 7.0	method B	positive mode
dopamine	100 mM	method B	positive mode
tryptamine	97.5 mM pH 7.0	method B	positive mode

Substrate	Stock solution used in assays	Method of preparation for LC/ESI-MS/MS	Ionization
serotonin	95 mM pH 7.0	method B	positive mode
D/L-tryptophan	40 mM	method B	positive mode
L-glutamine	50 mM	method B	positive mode
Acyl donors			
cinnamoyl-CoA	2.5 mM	depending on acyl acceptor	
p-coumaroyl-CoA	2.5 mM	depending on acyl acceptor	
caffeoyl-CoA	2.5 mM	depending on acyl acceptor	
feruloyl-CoA	2.5 mM	depending on acyl acceptor	
sinapoyl-CoA	2.5 mM	depending on acyl acceptor	
benzoyl-CoA	2.5 mM	depending on acyl acceptor	
acetyl-CoA	2.5 mM	depending on acyl acceptor	

Table S4 Conditions, compositions and detection parameters for the assays for pH and temperature optimum of NbHCT21

Parameter	Condition	Composition	Detection parameter
pH-optimum (KP_i-buffer)	ϑ = 25 °C<br>t = 30 min,<br>negative control:<br>t = 0 min *n* = 3	52 mM KP_i-buffer, pH 5.40-7.80<br>50 µM *p*-coumaroyl-CoA<br>60 mM (*SR*)-3-phenyl-lactic acid pH 7.0<br>2 µg NbHCT21	Injection volume: 50 µL<br>DAD-detector: λ = 312 nm<br>run time: t = 20 min<br>mobile phase: 55 % methanol, 0.01 % *o*-phosphoric acid<br>flow rate: 1 mL/min<br>stationary phase: Hypersil ODS C18 5 µm, 250 x 4 mm<br>column temperature: 35 °C
pH-optimum (Britton-Robinson-buffer)	ϑ = 25 °C<br>t = 30 min,<br>negative control:<br>t = 0 min *n* = 3	36 % (v/v) Britton-Robinson-buffer, pH 6.70-9.41<br>50 µM *p*-coumaroyl-CoA<br>60 mM (*SR*)-3-phenyl-lactic acid pH 7.0<br>2 µg NbHCT21	
temperature optimum	ϑ = 1.0-71.2 °C<br>t = 15 min,<br>negative control:<br>t = 0 min *n* = 3	52 mM KP_i-buffer, pH 7.90<br>50 µM *p*-coumaroyl-CoA<br>60 mM (*SR*)-3-phenyl-lactic acid pH 7.0<br>2 µg NbHCT21	

Table S5 Condition, composition and detection parameters for the assays for the substrate-saturation curves of NbHCT21

K_m for	with [corresponds to x times K_m-value]	Condition	Composition	Detection parameter
p-coumaroyl-CoA	(*R*)-3-phenyllactic acid [7.1]	ϑ = 25 °C<br>t = 30 min,<br>negative control:<br>t = 0 min<br>*n* = 3 x 3	32 mM KP_i-buffer, pH 7.0<br>1-100 µM *p*-coumaroyl-CoA<br>80 mM (*RS*)-3-phenyllactic acid<br>2 µg NbHCT21	Injection volume: 50 µL<br>DAD-detector: λ = 312 nm<br>run time: t = 20 min<br>mobile phase: 55 % methanol, 0.01 % *o*-phosphoric acid<br>flow rate: 1 mL/min<br>stationary phase: Hypersil ODS C18 5 µm, 250 x 4 mm<br>column temperature: ϑ = 35 °C
(*R*)-3-phenyl-lactic acid	*p*-coumaroyl-CoA [11]	ϑ = 25 °C<br>t = 30 min,<br>negative control:<br>t = 0 min<br>*n* = 3 x 3	32 mM KP_i-buffer, pH 7.0<br>100 µM *p*-coumaroyl-CoA<br>0.4-80 mM (*RS*)-3-phenyllactic acid<br>2 µg NbHCT21	
caffeoyl-CoA	(*R*)-3-phenyllactic acid [not applicable]	ϑ = 25 °C<br>t = 30 min,<br>negative control:<br>t = 0 min<br>*n* = 3 x 3	32 mM KP_i-buffer, pH 7.0<br>1-100 µM caffeoyl-CoA<br>80 mM (*RS*)-3-phenyllactic acid<br>2 µg NbHCT21	Injection volume: 50 µL<br>DAD-detector: λ = 333 nm<br>run time: t = 10 min<br>mobile phase: 55 % methanol, 0.01 % *o*-phosphoric acid<br>flow rate: 1 mL/min<br>stationary phase: Hypersil ODS C18 5 µm, 250 x 4 mm<br>column temperature: ϑ = 35 °C
(*R*)-4-hydroxy-phenyl-lactic acid	*p*-coumaroyl-CoA [not applicable]	ϑ = 25 °C<br>t = 45 min,<br>negative control:<br>t = 0 min<br>*n* = 3 x 3	32 mM KP_i-buffer, pH 7.0<br>100 µM *p*-coumaroyl-CoA<br>0.2-80 mM (*RS*)-4-hydroxyphenyl-lactic acid<br>3.9 µg NbHCT21	Injection volume: 50 µL<br>DAD-detector: λ = 312 nm<br>run time: t = 40 min<br>mobile phase: 40 % methanol, 0.01 % *o*-phosphoric acid<br>flow rate: 1 mL/min<br>stationary phase: Hypersil ODS C18 5 µm, 250 x 4 mm<br>column temperature: ϑ = 35 °C

Table S6 Predicted physicochemical properties of BAHD acyltransferases identified in this study (Expasy Protparam; web.expasy.org/protparam)

Protein	GenBank accession	Length [amino acid residues]	Native mole-cular mass [Da]	Calculated isoelectric point
NbHCT10	PX548088	442	49478.20	6.98
NbHCT11	PX548089	438	48484.10	6.14
NbHCT12	PX548090	435	48256.05	8.55
NbHCT13	PX548091	436	48398.13	6.98
NbHCT14	PX548092	431	48904.50	6.27
NbHCT15	PX548093	458	51537.06	6.29
NbHCT16	PX548094	452	50043.21	5.55
NbHCT17	PX548095	449	49034.69	6.56
NbHCT18	PX548096	473	51331.67	5.85
NbHCT19	PX548097	439	48325.82	5.65
NbHCT20	PX548098	436	48089.35	5.55
NbHCT21	PX548099	479	52815.29	5.88
NbHCT22	PX548100	471	51845.36	5.88
NbHCT23	PX548101	443	49167.63	6.88
NbHCT24	PX548102	521	57583.99	6.09
NbHCT25	PX548103	508	56554.69	6.13
NbHCT26	PX548104	450	50329.37	6.52
NbHCT27	PX548105	455	49647.94	7.19
NbHCT28	PX548106	471	52366.08	5.52
NbHCT29	PX548107	442	48911.87	5.62
NbHCT30	PX548108	544	60670.65	5.98
NbHCT31	PX548109	493	56127.20	5.84
NbHCT32	PX548110	441	49608.85	6.19
NbHCT33	PX548111	440	48639.38	6.35
NbHCT34	PX548112	439	49232.21	6.12
NbHCT35	PX548113	444	49858.31	6.17
NbHCT36	PX548114	445	49916.51	6.22
NbHCT37	PX548115	497	54518.00	5.66
NbHCT38	PX548116	511	56139.63	5.05
NbHCT39	PX548117	498	54712.77	5.38
NbHCT40	PX548118	482	53305.57	6.31
NbHCT41	PX548119	483	53318.46	5.55
NbHCT42	PX548120	579	62474.43	5.98
NbHCT43	PX548121	468	51607.92	5.98
NbHCT44	PX548122	524	57858.71	5.87
NbHCT45	PX548123	445	50156.55	6.33
NbHCT46	PX548124	444	50010.29	5.78
NbHCT47	PX548125	462	51572.96	6.52

Table S7 Summary of identified products formed in NbHCT10 assays and resulting LC/ESI-MS and LC/ESI-MS/MS data (negative ionization mode), *m/z* calculated with Compass DataAnalysis, products with less than $1.0 \cdot 10^3$ counts are considered traces and not listed, only fragments >10 % base peak are listed. [1)] Compared with external authentic standard

Detected product	Molecular formula	Retention time [min]	Calculated *m/z* $[M-H]^-$	Measured *m/z* $[M-H]^-$	Peak intensity m/z $[M-H]^-$ [counts]	LC/ESI-MS/MS *m/z* (% base peak)
cinnamoyl-5-*O*-shikimic acid	$C_{16}H_{16}O_6$	9.0	303.0863	303.1213	$8.3 \cdot 10^4$	MS2[303]: 147 (93), 137 (100), 111 (17)
p-coumaroyl-4-*O*-shikimic acid	$C_{16}H_{16}O_7$	7.7	319.0812	319.1173	$8.9 \cdot 10^4$	MS2[319]: 163 (14), 145 (100), 119 (21), 117 (25)
p-coumaroyl-5-*O*-shikimic acid [1)]	$C_{16}H_{16}O_7$	7.8	319.0812	319.1180	$1.9 \cdot 10^5$	MS2[319]: 163 (66), 145 (36), 137 (18), 119 (100), 117 (21)
caffeoyl-4-*O*-shikimic acid [1)]	$C_{16}H_{16}O_8$	7.2	335.0761	335.1110	$5.4 \cdot 10^4$	MS2[335]: 179 (22), 161 (100), 135 (13), 133 (11)
caffeoyl-5-*O*-shikimic acid [1)]	$C_{16}H_{16}O_8$	7.3	335.0761	335.1127	$1.4 \cdot 10^5$	MS2[335]: 179 (98), 161 (52), 135 (100)
feruloyl-5-*O*-shikimic acid	$C_{17}H_{18}O_8$	7.9	349.0918	349.1294	$1.1 \cdot 10^5$	MS2[349]: 193 (100), 178 (55), 175 (34), 160 (30), 149 (21), 137 (22), 134 (93)
sinapoyl-5-*O*-shikimic acid	$C_{18}H_{20}O_9$	7.9	379.1024	379.1407	$2.8 \cdot 10^4$	MS2[379]: 223 (95), 208 (86), 205 (30), 193 (15), 190 (27), 179 (19), 165 (12), 164 (100), 149 (30), 137 (26), 121 (19)
p-coumaroyl-5-*O*-quinic acid [1)]	$C_{16}H_{18}O_8$	7.2	337.0918	337.1358	$4.6 \cdot 10^4$	MS2[337]: 191 (100), 163 (16)
ester of *p*-coumaric acid and 2,5-dihydroxy-benzoic acid	$C_{16}H_{12}O_6$	10.1	299.0550	299.0933	$1.1 \cdot 10^3$	no MS2 event

Table S8 Summary of identified products formed in NbHCT11 assays and resulting LC/ESI-MS and LC/ESI-MS/MS data (negative ionization mode), *m/z* calculated with Compass DataAnalysis, products with less than $1.0 \cdot 10^3$ counts are considered traces and not listed, only fragments >10 % base peak are listed. [1)] Compared with external authentic standard

**Detected product**	**Molecular formula**	**Retention time [min]**	**Calculated *m/z* [M-H]⁻**	**Measured *m/z* [M-H]⁻**	**Peak intensity m/z [M-H]⁻ [counts]**	**LC/ESI-MS/MS *m/z* (% base peak)**
cinnamoyl-5-*O*-quinic acid	$C_{16}H_{18}O_7$	8.5	321.0969	321.1338	$4.1 \cdot 10^5$	MS2[321]: 173 (75), 147 (100)
p-coumaroyl-5-*O*-quinic acid [1)]	$C_{16}H_{18}O_8$	7.3	337.0918	337.1292	$2.2 \cdot 10^5$	MS2[337]: 191 (100), 163 (15)
caffeoyl-5-*O*-quinic acid [1)]	$C_{16}H_{18}O_9$	6.7	353.0867	353.1250	$1.3 \cdot 10^5$	MS2[335]: 191 (100)
feruloyl-5-*O*-quinic acid	$C_{17}H_{20}O_9$	7.4	367.1024	367.1412	$1.7 \cdot 10^5$	MS2[349]: 193 (20), 191 (100), 173 (12)
sinapoyl-5-*O*-quinic acid	$C_{18}H_{22}O_{10}$	7.3	397.1129	397.1532	$7.9 \cdot 10^4$	MS2[397]: 223 (21), 191 (100), 173 (13), 164 (11)
benzoyl-5-*O*-quinic acid	$C_{14}H_{16}O_7$	7.6	295.0812	295.1145	$6.5 \cdot 10^3$	MS2[295]: 177 (24), 173 (28), 121 (100)
p-coumaroyl-4-*O*-shikimic acid	$C_{16}H_{16}O_7$	7.6	319.0812	319.1222	$3.0 \cdot 10^3$	no MS2 event
p-coumaroyl-5-*O*-shikimic acid [1)]	$C_{16}H_{16}O_7$	7.7	319.0812	319.1217	$2.5 \cdot 10^3$	no MS2 event

Table S9 Summary of identified products formed in NbHCT12 assays and resulting LC/ESI-MS and LC/ESI-MS/MS data (negative ionization mode), *m/z* calculated with Compass DataAnalysis, products with less than $1.0 \cdot 10^3$ counts are considered traces and not listed, only fragments >10 % base peak are listed. 1) Compared with external authentic standard

Detected product	Molecular formula	Retention time [min]	Calculated *m/z* $[M-H]^-$	Measured *m/z* $[M-H]^-$	Peak intensity m/z $[M-H]^-$ [counts]	LC/ESI-MS/MS *m/z* (% base peak)
cinnamoyl-5-*O*-quinic acid	$C_{16}H_{18}O_7$	8.5	321.0969	321.1341	$3.2 \cdot 10^5$	MS2[321]: 173 (69), 147 (100)
p-coumaroyl-5-*O*-quinic acid 1)	$C_{16}H_{18}O_8$	7.3	337.0918	337.1294	$1.6 \cdot 10^5$	MS2[337]: 191 (100), 163 (14)
caffeoyl-5-*O*-quinic acid 1)	$C_{16}H_{18}O_9$	6.7	353.0867	353.1244	$5.4 \cdot 10^4$	MS2[335]: 191 (100)
feruloyl-5-*O*-quinic acid	$C_{17}H_{20}O_9$	7.4	367.1024	367.1405	$3.8 \cdot 10^4$	MS2[349]: 193 (17), 191 (100), 173 (11)
sinapoyl-5-*O*-quinic acid	$C_{18}H_{22}O_{10}$	7.3	397.1129	397.1527	$9.8 \cdot 10^3$	MS2[397]: 223 (21), 191 (100), 177 (16)
benzoyl-5-*O*-quinic acid	$C_{14}H_{16}O_7$	7.6	295.0812	295.1150	$6.1 \cdot 10^3$	MS2[295]: 173 (56), 121 (100)
acetyl-5-*O*-quinic acid	$C_9H_{14}O_7$	8.1	233.0656	233.0735	$4.5 \cdot 10^3$	MS2[233]: 179 (11), 165 (15), 148 (28), 147 (70), 120 (14), 119 (100)
p-coumaroyl-4-*O*-shikimic acid	$C_{16}H_{16}O_7$	7.6	319.0812	319.1212	$2.8 \cdot 10^3$	MS2[319]: 227 (19), 163 (13), 145 (100)
p-coumaroyl-5-*O*-shikimic acid 1)	$C_{16}H_{16}O_7$	7.7	319.0812	319.1212	$7.0 \cdot 10^3$	MS2[319]: 227 (16), 163 (48), 145 (37), 137 (22), 119 (100), 117 (38)
ester of *p*-coumaric acid and 2,5-dihydroxy-benzoic acid	$C_{16}H_{12}O_6$	10.1	299.0550	299.0929	$7.8 \cdot 10^2$	no MS2 event

Table S10 Summary of identified products formed in NbHCT13 assays and resulting LC/ESI-MS and LC/ESI-MS/MS data (negative ionization mode), *m/z* calculated with Compass DataAnalysis, products with less than $1.0 \cdot 10^3$ counts are considered traces and not listed, only fragments >10 % base peak are listed. 1) Compared with external authentic standard

Detected product	Molecular formula	Retention time [min]	Calculated *m/z* $[M-H]^-$	Measured *m/z* $[M-H]^-$	Peak intensity m/z $[M-H]^-$ [counts]	LC/ESI-MS/MS *m/z* (% base peak)
cinnamoyl-5-*O*-quinic acid	$C_{16}H_{18}O_7$	8.5	321.0969	321.1341	$3.9 \cdot 10^5$	MS2[321]: 173 (78), 147 (100)
p-coumaroyl-5-*O*-quinic acid 1)	$C_{16}H_{18}O_8$	7.3	337.0918	337.1295	$2.2 \cdot 10^5$	MS2[337]: 191 (100), 163 (18)
caffeoyl-5-*O*-quinic acid 1)	$C_{16}H_{18}O_9$	6.7	353.0867	353.1254	$1.3 \cdot 10^5$	MS2[335]: 191 (100)
feruloyl-5-*O*-quinic acid	$C_{17}H_{20}O_9$	7.4	367.1024	367.1415	$1.8 \cdot 10^5$	MS2[349]: 193 (23), 191 (100), 173 (20), 134 (10)
sinapoyl-5-*O*-quinic acid	$C_{18}H_{22}O_{10}$	7.3	397.1129	397.1538	$7.9 \cdot 10^4$	MS2[397]: 223 (22), 191 (100), 173 (15)
benzoyl-5-*O*-quinic acid	$C_{14}H_{16}O_7$	7.6	295.0812	295.1168	$1.2 \cdot 10^5$	MS2[295]: 173 (35), 121 (100), 111 (16)
p-coumaroyl-4-*O*-shikimic acid	$C_{16}H_{16}O_7$	7.6	319.0812	319.1230	$5.3 \cdot 10^4$	MS2[319]: 227 (19), 163 (13), 145 (100)
p-coumaroyl-5-*O*-shikimic acid 1)	$C_{16}H_{16}O_7$	7.7	319.0812	319.1228	$4.5 \cdot 10^4$	MS2[319]: 163 (60), 145 (45), 137 (18), 119 (100), 117 (20)
ester of *p*-coumaric acid and 2,5-dihydroxybenzoic acid	$C_{16}H_{12}O_6$	10.1	299.0550	299.0947	$3.1 \cdot 10^3$	no MS2 event

Table S11 Summary of identified products formed in NbHCT19 assays and resulting LC/ESI-MS and LC/ESI-MS/MS data (positive ionization mode), *m/z* calculated with Compass DataAnalysis, products with less than $1.0 \cdot 10^3$ counts are considered traces and not listed, only fragments >10 % base peak are listed

Detected product	Molecular formula	Retention time [min]	Calculated *m/z* [M+H]+	Measured *m/z* [M+H]+	Peak intensity m/z [M+H]+ [counts]	LC/ESI-MS/MS *m/z* (% base peak)
p-coumaroyl-ω-hydroxypalmitic acid	$C_{25}H_{38}O_5$	15.3	417.2636	417.3046	$1.8 \cdot 10^4$	MS2[417]: 271 (34), 163 (15), 145 (100), 117 (27)
caffeoyl-ω-hydroxypalmitic acid	$C_{25}H_{38}O_6$	14.4	433.2585	433.2981	$3.5 \cdot 10^3$	MS2[433]: 271 (19), 161 (100), 133 (16)
feruloyl-ω-hydroxypalmitic acid	$C_{26}H_{40}O_6$	15.5	447.2741	447.3163	$2.8 \cdot 10^4$	MS2[447]: 271 (19), 175 (100)
sinapoyl-ω-hydroxypalmitic acid	$C_{27}H_{42}O_7$	15.3	477.2847	477.3286	$1.2 \cdot 10^4$	MS2[477]: 462 (36), 271 (15), 205 (100)

Table S12 Summary of identified products formed in NbHCT20 assays and resulting LC/ESI-MS and LC/ESI-MS/MS data (positive ionization mode), *m/z* calculated with Compass DataAnalysis, products with less than $1.0 \cdot 10^3$ counts are considered traces and not listed, only fragments >10 % base peak are listed

Detected product	Molecular formula	Retention time [min]	Calculated *m/z* [M+H]+	Measured *m/z* [M+H]+	Peak intensity m/z [M+H]+ [counts]	LC/ESI-MS/MS *m/z* (% base peak)
p-coumaroyl-ω-hydroxypalmitic acid	$C_{25}H_{38}O_5$	15.3	417.2636	417.3054	$4.6 \cdot 10^4$	MS2[417]: 271 (34), 163 (10), 145 (100), 117 (25)
caffeoyl-ω-hydroxypalmitic acid	$C_{25}H_{38}O_6$	14.4	433.2585	433.3029	$7.0 \cdot 10^5$	MS2[433]: 271 (18), 161 (100), 133 (15)
feruloyl-ω-hydroxypalmitic acid	$C_{26}H_{40}O_6$	15.5	447.2741	447.3176	$2.4 \cdot 10^4$	MS2[447]: 271 (19), 175 (100)
sinapoyl-ω-hydroxypalmitic acid	$C_{27}H_{42}O_7$	15.3	477.2847	477.3221	$2.1 \cdot 10^4$	MS2[477]: 462 (36), 271 (15), 205 (100)

Table S13 Summary of identified products formed in NbHCT21 assays and resulting LC/ESI-MS and LC/ESI-MS/MS data (negative ionization mode), *m/z* calculated with Compass DataAnalysis, products with less than $1.0\cdot10^3$ counts are considered traces and not listed, only fragments >10 % base peak are listed. [1)] Compared with external authentic standard

Detected product	Molecular formula	Retention time [min]	Calculated *m/z* $[M-H]^-$	Measured *m/z* $[M-H]^-$	Peak intensity m/z $[M-H]^-$ [counts]	LC/ESI-MS/MS *m/z* (% base peak)
cinnamoyl-(*R*)-phenyllactic acid	$C_{18}H_{16}O_4$	11.9	295.0965	295.1345	$2.6\cdot10^5$	MS2[295]: 147 (100)
p-coumaroyl-(*R*)-phenyllactic acid	$C_{18}H_{16}O_5$	10.4	311.0914	311.1303	$2.2\cdot10^5$	MS2[311]: 165 (82), 147 (69), 145 (100), 119 (19), 117 (34)
caffeoyl-(*R*)-phenyllactic acid	$C_{18}H_{16}O_6$	9.9	327.0863	327.1263	$1.0\cdot10^5$	MS2[327]: 165 (39), 161 (100), 147 (28), 133 (19)
feruloyl-(*R*)-phenyllactic acid	$C_{19}H_{18}O_6$	10.5	341.1020	341.1429	$2.1\cdot10^5$	MS2[341]: 193 (11), 175 (77), 165 (78), 160 (100), 147 (55), 134 (18), 119 (12)
benzoyl-(*R*)-phenyllactic acid	$C_{16}H_{14}O_4$	11.3	269.0808	269.1160	$7.0\cdot10^4$	MS2[269]: 122 (13), 121 (100)
cinnamoyl-(*R*)-4'-hydroxyphenyllactic acid	$C_{18}H_{16}O_5$	10.4	311.0914	311.1289	$3.9\cdot10^4$	MS2[311]: 147 (11), 119 (100)
p-coumaroyl-(*R*)-4'-hydroxyphenyllactic acid [1)]	$C_{18}H_{16}O_6$	9.3	327.0863	327.1239	$2.6\cdot10^4$	MS2[327]: 181 (84), 163 (70), 145 (100), 135 (21), 119 (13), 117 (19)
caffeoyl-(*R*)-4'-hydroxyphenyllactic acid [1)]	$C_{18}H_{16}O_7$	8.9	343.0812	343.1206	$1.0\cdot10^4$	MS2[343]: 181 (74), 163 (30), 162 (10), 161 (100), 135 (16), 133 (22)
feruloyl-(*R*)-4'-hydroxyphenyllactic acid	$C_{19}H_{18}O_7$	9.4	357.0969	357.1374	$3.9\cdot10^4$	MS2[357]: 182 (11), 181 (100), 175 (79), 163 (49), 160 (69), 135 (20), 134 (11), 119 (10)
cinnamoyl-(*R*)-3',4'-dihydroxyphenyllactic acid	$C_{18}H_{16}O_6$	9.8	327.0863	327.1231	$8.6\cdot10^3$	MS2 [327]: 135 (100)

p-coumaroyl-(*R*)-3',4'-dihydroxyphenyllactic acid [1)]	$C_{18}H_{16}O_7$	8.9	343.0812	343.1188	$9.0 \cdot 10^3$	MS2[343]: 197 (29), 179 (38), 145 (100), 135 (31)
caffeoyl-(*R*)-3',4'-dihydroxyphenyllactic acid [1)] (rosmarinic acid)	$C_{18}H_{16}O_8$	8.4	359.0761	359.1136	$3.9 \cdot 10^3$	MS2[359]: 197 (22), 179 (19), 161 (100)
feruloyl-(*R*)-3',4'-dihydroxyphenyllactic acid	$C_{19}H_{18}O_8$	8.9	373.0918	373.1309	$1.5 \cdot 10^4$	MS2[373]: 197 (44), 179 (56), 175 (100), 160 (67), 135 (73), 123 (20)
ester of *p*-coumaric acid and D-glucaric acid	$C_{15}H_{16}O_{10}$	6.3	355.0660	355.1055	$5.1 \cdot 10^3$	MS2[355]: 209 (100), 193 (10), 191 (52), 165 (47), 163 (14), 147 (27)
ester of *p*-coumaric acid and mucic acid	$C_{15}H_{16}O_{10}$	6.2	355.0660	355.1099	$1.1 \cdot 10^3$	no MS2 event

Table S14 Summary of identified products formed in NbHCT22 assays and resulting LC/ESI-MS and LC/ESI-MS/MS data (positive ionization mode), *m/z* calculated with Compass DataAnalysis, products with less than $1.0 \cdot 10^3$ counts are considered traces and not listed, only fragments >10 % base peak are listed

Detected product	Molecular formula	Retention time [min]	Calculated *m/z* $[M+H]^+$	Measured *m/z* $[M+H]^+$	Peak intensity m/z $[M+H]^+$ [counts]	LC/ESI-MS/MS *m/z* (% base peak)
cinnamoyl-L-tryptophan	$C_{20}H_{18}O_3N_2$	10.3	335.1390	335.1442	$9.4 \cdot 10^4$	MS2[335]: 159 (29), 131 (100)
p-coumaroyl-L-tryptophan	$C_{20}H_{18}O_4N_2$	9.2	351.1339	351.1340	$7.3 \cdot 10^4$	MS2[351]: 147 (100), 119 (10)
p-coumaroyl-D-tryptophan	$C_{20}H_{18}O_4N_2$	9.2	351.1339	351.1336	$3.0 \cdot 10^3$	no MS2 event
caffeoyl-L-tryptophan	$C_{20}H_{18}O_5N_2$	8.9	367.1288	367.1299	$1.9 \cdot 10^5$	MS2 [367]: 205 (12), 188 (13), 163 (100), 135 (100)
feruloyl-L-tryptophan	$C_{21}H_{20}O_5N_2$	9.4	381.1445	381.1458	$9.4 \cdot 10^4$	MS2[381]: 178 (10), 177 (100), 145 (18)
sinapoyl-L-tryptophan	$C_{22}H_{22}O_6N_2$	9.3	411.1551	411.1541	$1.5 \cdot 10^4$	MS2[411]: 207 (100), 175 (21), 147 (11)

Table S15 Summary of identified products formed in NbCYP98A268 assays and resulting LC/ESI-MS and LC/ESI-MS/MS data (negative ionization mode), *m/z* calculated with Compass DataAnalysis, products with less than $1.0 \cdot 10^3$ counts are considered traces and not listed, only fragments >10 % base peak are listed. [1)] Compared with external authentic standard

Detected product	Molecular formula	Retention time product (substrate) [min]	Calculated *m/z* $[M\text{-}H]^-$	Measured *m/z* $[M\text{-}H]^-$	Peak intensity m/z $[M\text{-}H]^-$ [counts]	LC/ESI-MS/MS *m/z* (% base peak)
p-coumaroyl-*N*-3-hydroxyanthranilic acid + 1 O	$C_{16}H_{13}O_6N$	9.3 (9.9)	314.0659	314.1058	$3.3 \cdot 10^3$	MS2[314]: 161 (100), 146 (31), 135 (67), 134 (34), 133 (49), 132 (12), 108 (35)
p-coumaroyl-*N*-anthranilic acid + 1 O	$C_{16}H_{13}O_5N$	9.4 (10.0)	298.0710	298.1118	$1.0 \cdot 10^3$	no MS2 event
p-coumaroyl-tryptamine + 1 O	$C_{19}H_{18}O_3N_2$	8.9 (9.9)	321.1234	321.1656	$4.5 \cdot 10^4$	MS2[321]: 175 (18), 119 (100)
p-coumaroyl-*N*-tyramine + 1 O [1)]	$C_{17}H_{17}O_4N$	8.2 (8.7)	298.1074	298.1475	$1.7 \cdot 10^3$	no MS2 event

References

Busch T (2022) Investigations on the origin of the 3,4-dihydroxyphenyllactic acid moiety of rosmarinic acid in *Anthoceros agrestis*. PhD thesis, Philipps-Universität Marburg. https://doi.org/10.17192/z2023.0078

Busch T, Petersen M (2021) Identification and biochemical characterisation of tyrosine aminotransferase from *Anthoceros agrestis* unveils the conceivable entry point into rosmarinic acid biosynthesis in hornworts. Planta 253:98. https://doi.org/10.1007/s00425-021-03623-2

de Vries S, Fürst-Jansen JMR, Irisarri I, Dhabalia Ashok A, Ischebeck T, Feussner K, Abreu IN, Petersen M, Feussner I, de Vries J (2021) The evolution of the phenylpropanoid pathway entailed pronounced radiations and divergences of enzyme families. Plant J 107:975–1002. https://doi.org/10.1111/tpj.15387

Eberle D, Ullmann P, Werck-Reichhart D, Petersen M (2009) cDNA cloning and functional characterisation of CYP98A14 and NADPH: cytochrome P450 reductase from *Coleus blumei* involved in rosmarinic acid biosynthesis. Plant Mol Biol 69:239–253. https://doi.org/10.1007/s11103-008-9420-7

Janiak V, Petersen M, Zentgraf M, Klebe G, Heine A (2010) Structure and substrate docking of a hydroxy(phenyl)pyruvate reductase from the higher plant *Coleus blumei* Benth. Acta Cryst D66:593–603. https://doi.org/10.1107/S0907444910006360

Krogh A, Larsson B, Heijne G von, Sonnhammer EL (2001) Predicting transmembrane protein topology with a hidden Markov model: application to complete genomes. J Mol Biol 305:567–580. https://doi.org/10.1006/jmbi.2000.4315

Kruse LH, Weigle AT, Irfan M, Martínez-Gómez J, Chobirko JD, Schaffer JE, Bennett AA, Specht CD, Jez JM, Shukla D, Moghe GD (2022) Orthology-based analysis helps map

evolutionary diversification and predict substrate class use of BAHD acyltransferases. Plant J 111:1453–1468. https://doi.org/10.1111/tpj.15902

Ritter H, Schulz GE (2004) Structural basis for the entrance into the phenylpropanoid metabolism catalyzed by phenylalanine ammonia-lyase. Plant Cell 16:3426–3436. https://doi.org/10.1105/tpc.104.025288

Sander M (2010) Molekularbiologische und biochemische Untersuchungen von Hydroxycinnamoyltransferasen aus *Coleus blumei* und *Glechoma hederacea*. Dissertation, Philipps-Universität Marburg. https://doi.org/10.17192/z2010.0168

Schwarze CM, Petersen M (2024) Phenylalanine ammonia-lyases and 4-coumaric acid coenzyme A ligases in *Chara braunii*, *Marchantia polymorpha*, and *Physcomitrium patens* as extant model organisms for plant terrestrialization. Plant J 119:2797–2815. https://doi.org/10.1111/tpj.16950

Ufland M, Petersen M (2026) Identification and characterization of BAHD hydroxycinnamoyltransferases in the fern *Neoblechnum brasiliense*. Plant J 126:e70837. https://doi.org/10.1111/tpj.70837

Wang GQ, Chen JF, Yi B, Tan HX, Zhang L, Chen WS (2017) HPPR encodes the hydroxyphenylpyruvate reductase required for the biosynthesis of hydrophilic phenolic acids in *Salvia miltiorrhiza*. Chin J Nat Medicines 15:917–927. https://doi.org/10.1016/S1875-5364(18)30008-6

Wang X, Chao N, Zhang A, Kang J, Jiang X, Gai Y (2021) Systematic Analysis and Biochemical Characterization of the Caffeoyl Shikimate Esterase Gene Family in Poplar. Int J Mol Sci 22:13366. https://doi.org/10.3390/ijms222413366

Zhou J, Zou X, Deng Z, Duan L (2024) Analysing a Group of Homologous BAHD Enzymes Provides Insights into the Evolutionary Transition of Rosmarinic Acid Synthases from Hydroxycinnamoyl-CoA:Shikimate/Quinate Hydroxycinnamoyl Transferases. Plants 13:512. https://doi.org/10.3390/plants13040512
